# Analysis of Biosynthetic Gene Clusters, Secretory, and Antimicrobial Peptides Reveals Environmental Suitability of *Exiguobacterium profundum* PHM11

**DOI:** 10.3389/fmicb.2021.785458

**Published:** 2022-02-03

**Authors:** Alok Kumar Srivastava, Ruchi Srivastava, Akhilendra Pratap Bharati, Alok Kumar Singh, Anjney Sharma, Sudipta Das, Praveen Kumar Tiwari, Anchal Kumar Srivastava, Hillol Chakdar, Prem Lal Kashyap, Anil Kumar Saxena

**Affiliations:** ^1^Indian Council of Agricultural Research-National Bureau of Agriculturally Important Microorganisms, Maunath Bhanjan, India; ^2^Indian Council of Agricultural Research-Indian Institute of Wheat and Barley Research, Karnal, India

**Keywords:** antimicrobial peptides, biosynthetic gene clusters, *Exiguobacterium profundum*, plant growth promotion, signal peptides

## Abstract

Halotolerant bacteria produce a wide range of bioactive compounds with important applications in agriculture for abiotic stress amelioration and plant growth promotion. In the present study, 17 biosynthetic gene clusters (BGCs) were identified in *Exiguobacterium profundum* PHM11 belonging to saccharides, desmotamide, pseudaminic acid, dipeptide aldehydes, and terpene biosynthetic pathways representing approximately one-sixth of genomes. The terpene biosynthetic pathway was conserved in *Exiguobacterium* spp. while the *E. profundum* PHM11 genome confirms the presence of the 1-deoxy-d-xylulose 5-phosphate (DXP) pathway for the isopentenyl diphosphate (IPP) synthesis. Further, 2,877 signal peptides (SPs) were identified using the PrediSi server, out of which 592 proteins were prophesied for the secretion having a transmembrane helix (TMH). In addition, antimicrobial peptides (AMPs) were also identified using BAGEL4. The transcriptome analysis of PHM11 under salt stress reveals the differential expression of putative secretion and transporter genes having SPs and TMH. Priming of the rice, wheat and maize seeds with PHM11 under salt stress led to improvement in the root length, root diameters, surface area, number of links and forks, and shoot length. The study shows that the presence of BGCs, SPs, and secretion proteins constituting TMH and AMPs provides superior competitiveness in the environment and make *E. profundum* PHM11 a suitable candidate for plant growth promotion under salt stress.

## Introduction

The studies of extremophiles like *Exiguobacterium* are of high importance to address the potential of agricultural growth and production under abiotic stresses using these microorganisms for plant growth promotion (PGP) and stress amelioration (Hayat et al., 2012; Srivastava et al., 2019a,b). The genus *Exiguobacterium* possesses a variety of traits to combat environmental challenges and have been isolated from diverse environments, including permafrost, hot springs, oceans, the rhizosphere of plants, and food-processing plants as well as from alimentary canals (Vishnivetskaya et al., 2009). The presence of antimicrobial peptides (AMPs), secondary metabolite production, non-ribosomal peptide synthesis (NRPS), and polyketide (PK) synthesis provides extra-environmental traits to make it a suitable candidate for application in a variety of pharmaceutical, agricultural, and industrial applications (Kulshreshtha et al., 2013; Singh et al., 2013; Dastager et al., 2015; Meng et al., 2020). The comparative genome studies and advancements in many technologies have been exploited to identify NRPS, PK, antibiotic resistance, and salt tolerance in addition to PGP activities (Hugenholtz and Tyson, 2008; Menzel et al., 2016; Patel et al., 2018; Bharati et al., 2020).

In the last few decades, many secondary metabolite pathways have been identified in the microbes, which contribute toward its potential and environmental suitability (Pichersky et al., 2006; Dickschat, 2016). With the development of bioinformatics and biotechnologies, many secondary metabolite biosynthetic gene clusters (BGCs) have been characterized in bacteria. These clusters commonly contain a gene encoding one of several key signature enzymes (Yamada et al., 2015). The finding of BGCs in bacteria shows that the secondary metabolites are also widely distributed in microbes (Pichersky et al., 2006; Dickschat, 2016). These secondary metabolites are not necessary for the growth or development of the host but sometimes confer pathogenicity, virulence, and adaptation to the environment and play an important role in defense mechanisms (Osbourn, 2010; Wang and Lu, 2017).

The genome of *Exiguobacterium* sp. 8-11-1 has 134 genes for metabolism and inorganic ion transport, including five NhaC-type Na^+^/H^+^ antiporters and one MnhB-type Na^+^/H^+^ antiporter which allow bacteria to survive and grow at alkaline pH (Jiang et al., 2013). Most of the transporters have been identified with the signal peptides (SPs) with a secretory nature. These SPs have a role in the targeting of the protein, but the mechanism is still unknown (Galán et al., 2014). These secretion proteins possess transmembrane helices (TMHs) which have a role in the transportation of the substrate (Krampen et al., 2018). Further studies have shown the presence of PGP and biocontrol genes for auxin biosynthesis and siderophore and iron acquisition in different *Exiguobacterium* spp. (Tang et al., 2013; White et al., 2013). *E. chiriqhucha* N139 isolated from cold lakes is a recently reported species, and its genome was sequenced due to its stress defense ability, like growth in high concentrations of different metals especially arsenic, high ultraviolet B radiation, and salinity (Gutiérrez-Preciado et al., 2017). The genome of N139 contains arsenite efflux pump, ArsB, and ATPase. An ATPase that provides energy to ArsB for extrusion of arsenite and antimonite, ArsA, co-transcribed with ArsD, an arsenic chaperone for the ArsAB pump (Páez-Espino et al., 2009).

In previous studies, we have described the halotolerance potential of *E. profundum* PHM11 and its pan genome comparison with other genomes of *Exiguobacterium*. PHM11 exhibits a gene for shape determination (MreB), mannitol/L-proline pathways, carotenoid biosynthesis, and shikimate pathways (Patel et al., 2018). These studies have also described the impact of salinity on the expression of these genes. Further, we reported the presence of several pathways and its comparison with the already sequenced genome of *Exiguobacterium* (Srivastava et al., 2020). In the present study, different BGCs, secretory peptides (SPs), TMHs, and AMPs were mined from the genome of PHM11 with the aim to decipher its environmental suitability for PGP applications under salt stress. Transcriptome analysis and validation of different PGP traits in a pot trial were also conducted in rice, maize, and wheat.

## Materials and Methods

### Phylogenetic Tree Analysis

The 16S rRNA gene sequences of *Exiguobacterium* sp. were obtained from the NCBI, and alignment was implemented by ClustalW. The aligned file was subsequently used in neighbor joining tree construction by MEGA-X (Tamura et al., 2011). For the pairwise alignment of *E. profundum*, the sequence was aligned using the ClustalW server^1^.

### Comparative Genome Analysis Using the Genome-to-Genome Calculator

Genomes of all type strains of *Exiguobacterium* spp. were retrieved from NCBI. Only 12 complete or partial whole-genome sequences of the *Exiguobacterium* were found in the database. The genome of *E. profundum* PHM 11 was compared with eleven other type strains of *Exiguobacterium* using the genome-to-genome distance (GGD) distance, dDDH prediction, and model confidence interval between pairs of entirely or partially sequenced genomes. The values were calculated *in silico* by using the genome-to-genome distance calculator (GGDC) tool^2^. GGDC was performed for type strains of *Exiguobacterium* strains considering *E. profundum* PHM11 as a standard reference. GGDC functions on the basis of the distances from the set of HSPs or MUMs obtained by comparing each pair of genomes, transformed to values analogous to DDH.

### Datasets and Secondary Metabolite Biosynthetic Gene Clusters Finding

We obtained all finished *Exiguobacterium* genome sequences from EBI at http://www.ebi.ac.uk/genomes/archaea.html. The number and types of secondary metabolite BGCs in these sequences were identified by antiSMASH version 5.1.2, and the integrated ClusterFinder algorithm, a hidden Markov model-based probabilistic algorithm, was used to detect BGC-like regions in genomes (Cimermancic et al., 2014; Weber et al., 2015; Blin et al., 2017). The characterized and unknown secondary metabolite biosynthesis gene clusters in the *E. profundum* PHM11 were identified. Additionally, both the Known Cluster Blast and Cluster Blast modules were selected to identify similar clusters in sequenced genomes by genome comparisons. Further, domain functions and genetic similarities with known BGCs in studied gene clusters were predicted and annotated using antiSMASH 5.1.2.

### Signal Peptides and Transmembrane Prediction

The SP and its prediction for secretion were calculated using the PrediSi server^3^. All the protein sequences present in PHM11 were given as input file in FASTA format. The outputs represent a single numeric score, predicted cleavage site, and Boolean flag denoting CTT (co-translationally translocated) SP presence or absence. PrediSi is designed for extremely fast analysis and is well suited for high-throughput processing (Hiller et al., 2004). The transmembrane domain prediction was done using the TMHMM server^4^. The FASTA format of all the proteins present in PHM11 was uploaded on the server which uses cyclic hidden Markov models to predict membrane helices from the proteins.

### Mining of Antimicrobial Peptides From the Genome of *Exiguobacterium profundum* PHM 11

BAGEL 4 was used for the analysis of AMPs in genomic data. BAGEL is a web server that identifies putative bacteriocin ORFs in a DNA sequence using novel, knowledge-based bacteriocin databases and motif databases. Gene clusters of interest are discovered using the core-peptide database and/or through HMM motifs that are present in associated context genes.

### Validation for Antimicrobial Activity and Environmental Fitness of *Exiguobacterium profundum*

Validation for antimicrobial activity and environmental fitness of *E. profundum* PHM11 was done by an antagonism experiment against 63 bacterial and 31 fungal strains isolated from different environmental samples (Supplementary Table S1). A loop full of bacterial culture was inoculated in nutrient broth (NB) and incubated at 28°C for 72 h. Cell-free supernatant from NB was collected by centrifugation at 10,000 *g* for 10 min. The supernatant was sterilized by filtration through a 0.45-μm syringe filter. The extract obtained was screened for antibacterial activity by the well diffusion method. The test bacterial cultures were spread on a set nutrient agar plate, and immediately four wells of 5 mm were punched with a sterile borer, and the wells were filled with 20 μl of cell-free extract or NB (2 wells each). The plates were then incubated at 28–30°C for 18 h in the upright position. Inhibition was detected by a zone of clearing around the extract well (Baskaran et al., 2012). Effects of volatile organic compounds (VOCs) of *E. profundum* PHM11 on growth inhibition of thirty-one selected fungi were recorded. Seventy-two hour-old NA plates of *E. profundum* PHM11 and 48-h-old inoculated plates of test fungal isolates were used for inhibition assay. The sandwich plates were made under laminar airflow, and edges were sealed with parafilm to avoid any loss of VOCs. The plates were incubated at 28°C for 5 days keeping the fungal plate upward, and inhibition was recorded. Un-inoculated NA plates were taken as control (Ebadzadsahrai et al., 2020).

### Transcriptome Analysis of *Exiguobacterium profundum* PHM11

To investigate the transcriptional response of *E. profundum* PHM11 to osmotic stress, two RNA-seq libraries were generated in two different conditions, *viz.*, 0 mM NaCl and 100 mM NaCl. Cell samples were collected during the exponential phase when cultures reached enough biomass to assure the isolation of 100–500 ng of mRNA for RNA-seq library construction. Total RNA was isolated using the manufacturer’s protocol of the GeneJET RNA Purification Kit with slight modification, as described earlier (Bharati et al., 2016). Two biological replicates were used for transcriptome analysis. The *de novo* transcriptome sequencing was performed using the Illumina HiSeq platform in the paired-end module (Liu et al., 2014).

The raw fastq files were processed before performing assembly. Prior to the assembly, base trimming, removal of adapter sequences, and filtering out of reads with an average quality score less than 30 were performed in every paired end read. Further, the rRNAs were removed from the generated sequences based on the reference ribosomal RNA sequences available in the SILVA database (Quast et al., 2013). The cleaned reads were aligned (length ≥ 200 bp) using the Bowtie2 program (Langmead and Salzberg, 2012), normalized, and finally subjected to *de novo* transcriptome assembly using Trinity (Grabherr et al., 2011; Huang et al., 2016). Normalization was performed using the variance analysis package EdgeR program (Robinson et al., 2010). The assembled transcripts were annotated using BLASTX against the non-redundant nucleotide databases. The gene ontology (GO) annotation for Molecular Function (MF), Cellular Component (CC), and Biological Process (BP) for upregulated transcripts was performed using the gene ontology tool (Ashburner et al., 2000). DESeq was used for the differential gene expression analysis (Anders and Huber, 2010). The gene considered differentially expressed if the *p*-value for differential expression was <0.05 and the absolute log (base 2)-fold change was ≥1. The final figure was represented in the form of heatmap.

### Evaluation of Plant Growth-Promoting Attributes of *Exiguobacterium profundum* PHM 11

In order to evaluate PGP attributes of PHM11, a pot experiment was designed using rice (cv. Rajendra sweta), maize (cv. Bio9637), and wheat (cv. HD2967). The seeds were surface sterilized with 0.5% (v/v) NaOCl for 10 min and were subsequently washed with sterile deionized water. Seeds were air dried and coated with active culture (10^8^ cfu ml^–1^) of *E. profundum* PHM11 suspended in 1% CMC. Approximately 15 seeds were sown per pot, and 90% germination was observed. Plant growth characteristics like total root length, root diameter, projected area, surface area, number of forks, and number of links were determined using the root scanner (Epson Expression 12000XL scanner with WinRHIZO Pro software) for the treated (+) and non-treated (–) in both conditions of salt absence (–) and presence (+) (100 mM NaCl) after 28 days of sowing. The stem length and total chlorophyll content (using a chlorophyll meter, Apogee Instruments, Logan, UT, United States) were also measured. All the laboratory and greenhouse experiments were executed in three replicates.

## Results and Discussion

### Phylogenetic Affiliation of *Exiguobacterium profundum* PHM11

**FIGURE 1:**
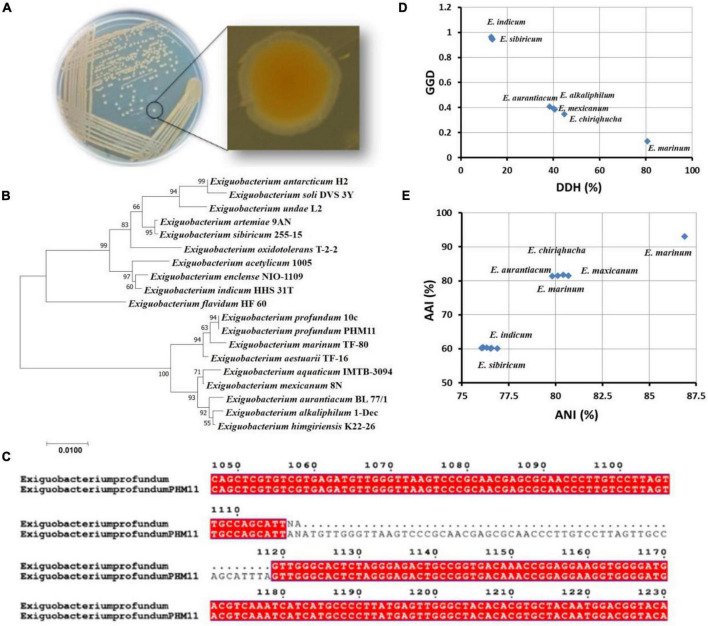
Genome comparison of type strains of *Exiguobacterium* spp. **(A)** Colony shape and pigmentation of the strain. **(B)** Phylogenetic tree analysis of type strains of *Exiguobacterium* spp. using MEGA-X using the 16S rRNA sequence. The sequences were aligned by ClustalW, and the neighbor-joining method was used for the phylogenetic tree preparation. The stars represent branching of the strains in two major groups. **(C)** The pairwise sequence alignment of the 16S rRNA sequence of *E. profundum* type strain (10C) and the *E. profundum* PHM11. The alignment was done using ClustalW, then the alignment file was submitted to ESPript, and a final figure was generated. **(D)** Represents the graph between GGD and DDH of twelve type strains of *Exiguobacterium* species with reference to the *E. profundum* PHM11, and **(E)** represents AAI vs. ANI graph of 12 type strains of *Exiguobacterium* species with reference to the *E. profundum* PHM11.

The identity of PHM11 isolated from the soil collected from Mau, Uttar Pradesh, was established as *Exiguobacterium profundum* on the basis of 16S rRNA sequencing followed by whole-genome sequencing (NCBI accession no. MRSV00000000) (Patel et al., 2018). *E. profundum* PHM11 was characterized as the bacilli forming a light-orange colony with optimal growth at 37°C (Figure 1A). PHM11 was tested positive for catalase and oxidase enzymatic activities. The PGP activities like phosphate solubilization and zinc solubilization as well as siderophore, ammonia, and HCN production were checked and found positive (Table 1). Similar PGP activities have been already reported in many *Exiguobacterium* spp. (Tang et al., 2013; White et al., 2013). The comparison of PHM11 with other eighteen type species of *Exiguobacterium* on the basis of the 16S rRNA sequence put it with the *E. profundum* (Figure 1B). Phylogenetic tree analysis showed the two major branching (Figure 1B) and the two branches further divided into two clades. The two branches have an intermediate species, *E. flavidum*. *E. profundum* shares a clade with *E. marinum* and *E. aestuarii*. The 16S rRNA sequence similarity between the PHM11 and the *E. profundum* (10C) type species by pairwise sequence alignment (Figure 1C) revealed that 58 nucleotides were missing (gap) in the type species.

**TABLE 1:** General and PGP traits of *Exiguobacterium profundum* PHM11.

Phenotypic characters	*Exiguobacterium profundum* PHM11
Colony morphology (shape)	Round
Colony color Growth temperature	Light orange 28–37°C
Gram test	(Mesophile) +ve
Catalase test Oxidase test	+ve +ve
Motility test Phosphate solubilization	+ve +ve
Siderophore production Ammonia production	+ve +ve
Zinc solubilization HCN production	+ve +ve

Furthermore, out of 18 type species of *Exiguobacterium*, the whole genomes of only 12 species were available in the NCBI database. Comparison of these twelve genomes with *E. profundum* PHM11 on the basis of the GGD and DDH showed the closeness of *E. marinum* with PHM11 having the 80.6% DDH value, 0.1296 GGD, and model CI value [76.6 – 84%] (Figure 1D and Table 2). *E*. *chiriqhucha*, *E. aurantiacum*, *E. mexicanum*, and *E. alkaliphilum* were found the second closest to *E. profundum* PHM11 which showed ∼40% DDH value, ∼0.4 GGD, and model CI value [10.7 – 16.8%]. The rest of the strains *E. sibricum*, *E. undae*, *E. antarcticum*, *E. indicum*, *E. oxidotolerans*, *E. acetylicum*, and *E. enclense* showed less than 14% DDH, more than 0.9 GGD, and a model CI value in the range of 10–17%. The result was further confirmed with the comparison of the ANI value and AAI value with reference to PHM11 (Figure 1E). The graph between ANI and AAI showed a similar pattern to that of the DDH and GGD graph. The graph indicated the genomic differences between the species as well. Because of the genomic differences, they possess a variety of habitat, PGP traits, and environmental suitability (Pichersky et al., 2006; Vishnivetskaya et al., 2009; Dickschat, 2016).

**TABLE 2:** List of some bacteriocin identified in the PHM 11 genome.

Bacteriocin	No.	Bacteriocin	No.
Putative_bacteriocin	11	Lactococcin_MMFII	2
Cerecidin	9	Leucocin_A_(LeucocinA-UAL187)	2
Propionicin_SM1	5	Mutacin_IV	2
Putative_lantibiotic	5	Mutacin1140_(Mutacin_III)	2
UviB	5	Plantaricin_C19	2
BlpM	4	Plantaricin_E	2
Enterocin_X_chain_beta	4	Plantaricin_NC8-alpha	2
Lissoclinamide2/3_patE3	4	Plantaricin_W	2
Prochlorosin	4	Protease-activated antimicrobial protein(PAMP)	2
Propionicin-F	4	Pumilarin	2
Acidocin_LF221B(GassericinK7B)	3	Ruminococcin_A_	2
Bacteriocin_LS2chaina	3	Sakacin_G_skgA2	2
Bottromycin	3	Sakacin_P	2
Bovicin_255_variant	3	Sakacin_P_(Sakacin674)	2
Geobacillin_II	3	Salivaricin_A4	2
Staphylococcins_C55a_SacaA	3	Salivaricin_A5	2
AcidocinA	2	Salivaricin_G32	2
AFP-1	2	SalivaricinA	2
Anacyclamide_A20P_AcyE_(Anacycla mide_A20PP_AcyE)	2	Enterocin_B	2
Arthrospiramides_A_ArtE3	2	Enterocin_Nkr-5-3B	2
Arthrospiramides_B_ArtE5	2	Epicidin280	2
Aureocin_A53	2	FrEUN1f_DRAFT_0188_putative_Linari din	2
Auto_Inducing_Peptide_I	2	Colicin V	1
Bacteriocin_J46	2	Boticin_B	1
Butyrivibriocin	2	Bottromycin_D	1
Carnocin_CP52	2	Bovicin_255_peptide	1
Carnolysins	2	Butyrivibriocin_AR10	1
Cyanothecamide_A_ThcE2	2	Carnobacteriocin_BM1_(Carnobacterioci nB1)	1
Cyanothecamide_B_ThcE2	2	Carnocyclin-A	1
EJ97enterocin	2	Caulonodin_II	1
Enterocin	2	Caulosegnin_III	1
Enterocin_1071B	2	Cinnamycin_B	1
Enterocin_AS-48	2	Circularin_A	1
Ipomicin	2	Coagulin_CoaA	1
Halocin-C8	2	CoagulinA	1

### Metabolite Analysis of PHM11 Identified Putative Biosynthetic Gene Clusters

**FIGURE 2:**
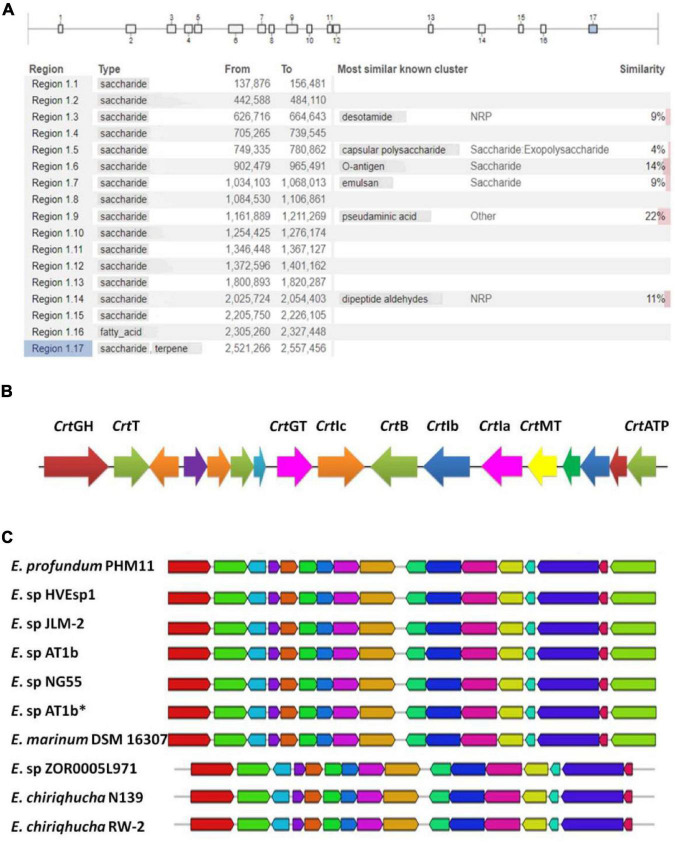
Identification of putative biosynthetic gene clusters (BGCs) using anti-SMASH. **(A)** Represents the presence of the 17 BGCs across the *Exiguobacterium profundum* PHM11 genome. **(B)** Represents the 17th cluster of the BGC representing the phytoene synthase (*crtB*), phytoene desaturase (*crtI*), glycoside hydrolase (*crtGH*), MATE family efflux transporter (*crtT*), glycosyltransferase (*crtGT*), phytoene desaturase (*crtIa*, *crtIb*, *crtIc*), phytoene synthase (*crtB*), class I SAM-dependent methyltransferase (*crtMT*), and ATP-binding cassette domain-containing protein (*crtATP*). **(C)** Represents the conservation of the clusters in different *Exiguobacterium* spp. ‘*’ is used to differentiate *Exiguobacterium* sp. AT1b submitted with accession number MOEL01 (Assembly GCA_001908175.1) in the NCBI database.

Our earlier reports identified *E. profundum* PHM11 a rich source of the carotenoids, and the production was enhanced under salt stress (Patel et al., 2018). This led us to mine additional secondary metabolite pathways. In order to mine the secondary metabolite pathways, the *E. profundum* PHM11 genome was analyzed through antiSMASH version 5.1.2 and identified seventeen regions in the genome having putative BGCs with 514 genes (Figure 2A and Supplementary Table S2). Most of the BGCs (15) corresponded to saccharide type; one BGC corresponded to each fatty acid and terpene biosynthesis. Regions 1.3, 1.5, 1.6, 1.7, 1.9, and 1.14 belonged to most similar known clusters of desotamide, capsular polysaccharides, O-antigen, emulsan, pseudaminic acid, and dipeptide aldehydes, respectively. All these BGCs comprised approximately 16.6% of the genome. Region 1.6 is the largest one with ∼63 kb having more than 157 genes which belonged to the *o*-antigen and polysaccharide production, while region 1.1 was the smallest BGC with ∼19 kb nucleotides only having 19 genes in the cluster.

Regions 1.3, 1.9, and 1.14 represented the presence of the clusters of gene for the biosynthesis of desotamides, dipeptide aldehydes, PK, and NRPS. Desotamides are a natural cyclohexapeptide antibiotic which has a role in the inhibition of certain microbes (Chen et al., 2018). Dipeptide aldehydes and the non-ribosomal peptides are a diverse family of natural products that fall into the class of secondary metabolites with diverse properties as toxins, siderophores, pigments, antibiotics, immune-suppressants, and anticancer agents (Bushley and Turgeon, 2010; Wang et al., 2014; Martínez-Núñez and López, 2016). Similarly, PKs are polycyclic aromatic natural products having anticancerous, antibacterial, antifungal, antiviral, antiparasitic, and other medicinally significant properties synthesized by PK synthases (PKSs) in soil-borne bacteria (Das and Khosla, 2009).

In PHM11, we observed a number of traits for PK as well as non-ribosomal protein synthesis. Other closely related Firmicutes are also known to abundantly produce various NRPS and PKs. Genome mining studies have indicated that 31% of the Firmicutes harbor NRPS and PK secondary metabolite gene clusters (Wang et al., 2014). Various members of the genus *Bacillus* like *B. subtilis*, *B. thuringiensis*, *B. cereus*, *B. velezensis*, and *B. licheniformis* are well known for their ability to produce an array of non-ribosomal and PK antimicrobial compounds. It has been reported that almost 10% of the genome of *B. velezensis* FZB42 is for the synthesis of different antimicrobial compounds (Borriss et al., 2019). Non-ribosomal peptides (NRPs) are synthesized through enzyme-mediated condensation of amino acid residues where > 300 different precursor molecules help in the assemblage of NRPs (Saxena et al., 2020). A number of researchers indicated that cyclic lipopeptides are one of the most abundant NRPs in *Bacillus* (Mora et al., 2015; Solanki et al., 2015; Saxena et al., 2017, 2020). The genomes of *B. subtilis*, *B. thuringiensis*, *B. velezensis*, and *B. cereus* present high abundance of NRP gene clusters (Zhao and Kuipers, 2016). Among the lipopeptides, iturin and surfactin are the most abundant in *Bacillus* (Mora et al., 2011; Kushwaha et al., 2020; Saxena et al., 2020). Bacillaene, Difficidin, and Macrolactin are some of the important PK antimicrobial compounds produced by various species of *Bacillus* (Chen et al., 2006; Wang et al., 2015). *B. amyloliquefaciens* and *B. subtilis* were identified as prolific producers of PKs. However, other species like *B. atrophaeus* and *B. mojavensis* are also known to produce PKs (Wang et al., 2015). The genome (3.92 Mb) of *B. amyloliquefaciens* FZB42 dedicated ∼200 kb toward production of PKs. The genomic and transcriptomic data of *E. profundum* PHM11 presented here provide insight for the need of quantitative shotgun proteomics to further validate and improve the protein identification and integration to their functional context. It has been suggested that changes in gene expression do not necessarily correlate with the adaptive manifestations, while proteins are rather more directly related (Evans, 2015). Further, proteomic analysis are capable of estimating absolute protein amounts and help in the analysis of low-abundance and modified proteins which can be of high biological relevance (Semanjski and Macek, 2016).

### *Exiguobacterium profundum* PHM11’s Biosynthetic Gene Clusters Contain Genes Related to Environmental Fitness

The third cluster (Region 1.3) contained 42 genes in ∼38-kb size (Figure 2A). This cluster contained genes for the desotamides and NRPs, which includes diguanylate cyclase, sugar ABC transporter ATP-binding protein, tryptophan 2,3-dioxygenase, and kynureninase (Supplementary Table S2). The NRPs generally contain amino acids like ornithine or amino acids, and their structures are macrocyclic, or branched macrocyclic, dimers or trimers of identical structural elements (Singh et al., 2012). The presence of arginine–ornithine antiporter participates in the direct supply of the ornithine, while the presence of arginine deiminase and ornithine carbamoyltransferase genes supports the biosynthesis (Bushley and Turgeon, 2010; Wang et al., 2014). Another enzyme UDP-*N*-acetylmuramoyl-L-alanyl-D-glutamate-2,6-diaminopimelate ligase (MurE) was also present which catalyzed a reaction in the cytoplasmic step of peptidoglycan biosynthesis by adding the third amino acid residue to the peptide stem (McGroty et al., 2013). The 9^th^ cluster (Region 1.9) contained 59 genes in 49 kb of stretch along with the pseudaminic acid synthase (Figure 2A). Pseudaminic acid synthase catalyzes the condensation of phosphoenolpyruvate (PEP) with the hexose, 2,4-diacetamido-2,4,6-trideoxy-L-altrose, to form pseudaminic acid and phosphate (Chou et al., 2005). The isomers of the pseudaminic acid are part of the lipopolysaccharide, and their production is important for the invasiveness of pathogenic organisms (Knirel et al., 1997).

Besides these proteins, this cluster contained genes for antibiotic biosynthesis monooxygenase (ABM) and multidrug resistance efflux transporter family protein which participated in the drug metabolism and transport (Supplementary Table S2). The ABM has the fidelity control during aromatic PK biosynthesis (Qin et al., 2019). The 14^th^ cluster (Region 1.14) contained 30 genes spanning ∼29 kb (Figure 2A). These genes included asparagine–tRNA ligase, pyridoxal phosphate-dependent aminotransferase, aspartate 1-decarboxylase, pantoate-beta-alanine ligase, CCA tRNA nucleotidyltransferase, *N*-acetyl-alpha-D-glucosaminyl L-malate synthase (BshA), bacillithiol biosynthesis deacetylase (BshB1), tetratricopeptide repeat protein, and 3-dehydroquinate synthase which participated in the PK and NRP synthesis (Supplementary Table S2). The cluster also possessed *N*-acetyl-alpha-D-glucosaminyl L-malate synthase (BshA) and bacillithiol biosynthesis deacetylase (BshB1) which had key roles in the biosynthesis of bacillithiol (BSH) and low-molecular-weight (LMW) thiols (Gaballa et al., 2010). LMW thiols play critical roles in cell physiology during oxidative stress. In most cells, the cytosol is reducing and protein thiols are largely in the reduced state (Dalle-Donne et al., 2009). During the oxidative stress, the disulfide bond formation between cysteine thiols provides stability and determines the structure of extra-cytoplasmic proteins (Meyer et al., 2009). Thus, the BSH provides protection to the cell against oxidative stress. Furthermore, this cluster possessed 3-dehydroquinate synthase which catalyzed the second step in the shikimate pathway, which was essential for the production of aromatic amino acids in bacteria, plants, and fungi, but not mammals (Liu et al., 2008). This makes it an ideal target for new antimicrobial agents, antiparasitic agents, and herbicides. Previously, our group reported that the genes related to the shikimate pathway get upregulated upon the salinity stress (Patel et al., 2018).

In order to further investigate the AMPs, BAGEL 4 was used for the analysis of AMPs in genomic data. A total 181 bacteriocins were identified in the *E. profundum* PHM11 genome out of which eleven putative bacteriocins and nine cerecidins were identified (Table 2). We also observed the presence of cerecidin which was a lantibiotic, ribosomally synthesized and posttranslationally modified AMPs (Table 2). Validation for antimicrobial activity and environmental fitness of *E. profundum* PHM11 was done by antagonism experiment against a wide range of fungal and bacterial strains isolated from environmental samples. Out of 94 test organisms (63 bacteria and 31 fungi), *E. profundum* PHM11 was found highly competitive against 43 bacteria and 26 fungi (Supplementary Table S1 and Supplementary Figure S1).

### Terpene Biosynthesis Cluster Is Conserved in *Exiguobacterium* spp.

The 17^th^ BGC represented the genes belonging to the saccharide as well as terpene biosynthesis. This region was covered between 2,521,526 and 2,557,456 bp having 31 genes (Supplementary Table S2). The terpene cluster was present between 2,536,720 and 2,556,936 bp having 17 genes (Figure 2B and Table 3). These 17 genes could be categorized in the core biosynthetic gene, additional biosynthetic genes, transport-related genes, regulatory genes, and other genes. Squalene/phytoene synthase (*crt*B) is the core biosynthetic gene which is responsible for the converting phytoene into lycopene, an important stage in carotene biosynthesis (Nakashima et al., 1995; Pan et al., 2015). Some additional biosynthetic genes, namely, glycoside hydrolase family 13 protein (*crt*GH), enoyl-CoA hydratase/isomerase family protein, glycotransferase (*crt*GT), phytoene desaturase (*crt*Ia, *crt*Ib, and *crt*Ic, three copies at different locations), and class I SAM-dependent methyltransferase (*crt*MT), were also present. Two transport-related genes, namely, MATE family efflux transporter (*crt*T) and ATP-binding cassette domain-containing proteins, were present (*crt*ATP). Two regulatory proteins, GAF domain-containing protein and some other genes, *cys-t-RNA* (Pro) deacylase, CDP-diacylglycerol–serine *O*-phosphatidyl transferase, carotenoid biosynthesis protein, and 1-acyl-sn-glycerol-3-phosphate acyltransferase gene, were present in this cluster (Figure 2B). This cluster had 100% similarity with that of the other *Exiguobacterium* spp. JLM2, HVEsp1, AT1b, ZOR0005, and NG55; *E. marinum* DSM 16307; and *E. chiriqhucha* RW-2 (Figure 2C). The results indicated that all the genes present in this cluster are ∼100% similar in the *Exiguobacterium* species which has been characterized for the isolates closely related with *E. profundum* PHM11 in our previous studies (Srivastava et al., 2020).

**TABLE 3:** List of identified genes in 17th BGCs (Region 1.17) of the terpene synthesis pathway.

Table of genes	Locations From	To	Strands	Annotations of query cluster
BBB58_RS13090	2536720	2538505	+	Glycoside hydrolase family 13 protein
BBB58_RS13095	2538664	2540023	+	MATE family efflux transporter
BBB58_RS13100	2540046	2540781	–	Enoyl-CoA hydratase/isomerase family protein
BBB58_RS13105	2540922	2541396	+	Cys-tRNA(Pro) deacylase
BBB58_RS13110	2541415	2542114	+	CDP-diacylglycerol–serine O-phosphatidyltransferase
BBB58_RS13115	2542203	2542953	+	Carotenoid biosynthesis protein
BBB58_RS13120	2542918	2543617	+	1-Acyl-sn-glycerol-3-phosphate acyltransferase
BBB58_RS13125	2543613	2544681	+	Glycosyltransferase
BBB58_RS13130	2544707	2546174	+	Phytoene desaturase
BBB58_RS13135	2546616	2547456	–	Squalene/phytoene synthase family protein
BBB58_RS13140	2547424	2548903	–	Phytoene desaturase
BBB58_RS13145	2548899	2550393	–	Phytoene desaturase
BBB58_RS13150	2550462	2551494	–	Class I SAM-dependent methyltransferase
BBB58_RS13155	2551594	2551978	–	YisL family protein
BBB58_RS13160	2552091	2554650	–	GAF domain-containing protein
BBB58_RS13165	2554646	2555009	–	Response regulator
BBB58_RS13170	2555142	2556936	–	ATP-binding cassette domain-containing protein

### ***Exiguobacterium*** *profundum* PHM11 Possesses the 1-Deoxy-D-xylulose 5-phosphate Pathway for IIP and Carotene Biosynthesis

With the rapid development of biocatalysis, β-carotene production through biosynthetic methods has become an active field, and several studies regarding genetic modification to enhance microbial production of β-carotene or carotenoids have been reported (Yang and Guo, 2014). *E. profundum* PHM11 showed an increased total carotenoid concentration in 100-mM salinity-affected cells (Patel et al., 2018). Cluster 17 shows high similarity to terpene BGCs. Carotenoids are terpenoids found in all photosynthetic organisms and also in some non-phototrophic organisms. The intermediate units for the carotenoids are the IPP units. We observed the presence of the 1-deoxy-d-xylulose 5-phosphate (DXP) pathway and the presence of enzymes responsible for the catalysis of each step of the production of IPP and DAMPP units (Table 4). The first step of the synthesis of the IPP units is the condensation and decarboxylation reaction between the D-glyceraldehyde phosphate and the pyruvate carried out by DXPS (1-deoxy-D-xylulose-5-phosphate synthase) and the production of DXP. The formation of DXP has been observed in many bacteria, and due to the formation of DXP, the pathway is also known as DXP pathway (Rohmer, 1999). These are responsible for the synthesis of the carotenoids as well. In the second step, DXP is converted to MEP by the enzyme DXR (1-deoxy-D-xylulose 5-phosphate reductoisomerase). The third step represents the formation of 4-diphosphocytidyl-2-*C*-methyl-D-erythritol from MEP, which is catalyzed by ispD (2-*C*-methyl-D-erythritol 4-phosphate cytidylyltransferase). Similarly, the 4^th^, 5^th^, and 6^th^ steps are catalyzed by ispE, ispF, and ispH, respectively, and after the 6^th^ step HMBPP was produced which is the substrate for the formation of IPP and DAMPP by the enzyme ispH (4-hydroxy-3-methylbut-2-enyl diphosphate-reductase). Cluster 17 represented the presence of the squalene/phytoene synthase (*crt*B) and phytoene desaturase (*crt*I) which is responsible for the conversion of the phytoene to lycopene and then lycopene into carotene, respectively (Figure 2B). We were unable to trace the enzyme geranylgeranyl diphosphate synthase (*crt*E) in genomes as well as in cluster analysis which is responsible for the formation of GGDP from the condensation IIP and DMAPP (Nakashima et al., 1995; Pan et al., 2015). Furthermore, we observed the presence of 1-hydroxy-2-methyl-2-(*E*)-butenyl 4-diphosphate synthase (*crt*G) enzyme which could catalyze the efficient conversion from β-carotene to 2,2-dihydroxy β-carotene (Table 4).

**TABLE 4:** List of enzymes participating in the DPX pathway in *E. profundum* PHM11.

S no	From	To	Frame	Operan name	Enzyme
1	485940	486821	+	ispE	4-diphosphocytidyl-2-*C*-methyl-D-erythritol kinase
2	523853	524533	+	ispD	2-*C*-methyl-D-erythritol 4-phosphate cytidylyltransferase
3	524530	525003	+	ispF	2-*C*-methyl-D-erythritol 2,4-cyclodiphosphate synthase
4	1527288	1526389	–	ispH	4-hydroxy-3-methylbut-2-enyl diphosphate reductase
5	1590002	1591903	+	dxps	1-deoxy-D-xylulose 5-phosphate synthase
6	2109450	2108293	–	dxr	1-deoxy-D-xylulose 5-phosphate reductoisomerase
7	1543472	1542351	–	crtG	1-hydroxy-2-methyl-2-(*E*)-butenyl 4-diphosphate synthase

### Identification of Signal Peptides and Transmembrane Helix Supports the Targeted Substrate Transport

**FIGURE 3:**
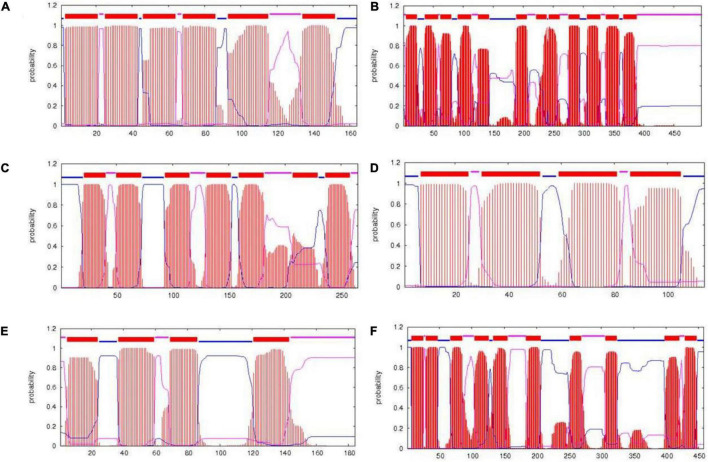
Transmembrane structure predicted by the TMHMM server. The transmembrane domains of rod-shaped-determining protein MreD **(A)**, potassium/proton antiporter **(B)**, ABC transporter permease subunit **(C)**, multidrug efflux SMR transporter **(D)**, isoprenylcysteine carboxyl methyltransferase family protein **(E),** and Na^+^/H^+^ antiporter NhaC **(F)**. Red color represents the transmembrane part, blue color represents the part of protein inside the cell, and pink color represents the part outside the cell.

PrediSi predicted 2,877 proteins with SPs having a cutoff score of 0.5. Out of these, 592 were predicted for the secretion and among them, 160 were hypothetical proteins (Supplementary Table S3). The major secretory proteins with the highest scores were rod-shaped-determining protein (*MreD*), Na/H^+^ antiporter, alpha-amylase, nitrous oxidase accessory protein, iron-siderophore ABC transporter substrate-binding protein, and transporter SBD (substrate binding domain-containing) protein with a score more than 0.9 (Table 5). Along with these, the multidrug efflux SMR transporter, peptidoglycan glycosyltransferase, type I pullulanase, zinc metalloprotease HtpX, zinc ABC transporter solute-binding protein, flagellar type III secretion system pore protein (FliP), and type IV secretory system conjugative DNA transfer family protein were predicted for SPs with secretion having TMH. The result indicated that most of the transporters related to the PGP activity and heavy metal resistance were predicted to have TMH for secretion (Diepold et al., 2015; Krampen et al., 2018). The transmembrane domain of some of the top score-predicted secretion protein was shown (Figure 3). *MreD* had six TMHs (Figure 3A), the potassium/proton antiporter had twelve TMHs (Figure 3B), ABC transporter permease subunit had seven TMHs (Figure 3C), multidrug efflux SMR transporter had four TMHs (Figure 3D), isoprenylcysteine carboxyl methyltransferase family protein had four TMHs (Figure 3E) and Na+/H+ antiporter NhaC had ten TMHs (Figure 3F).

**TABLE 5:** List of proteins with signal peptide prediction using PrediSi.

FASTA ID	Protein name	Score	Cleavage position	Peptide sequence
WP_04975998 2.1	Rod-shape-determining protein MreD	1	18	MKVFIALFLLFLIEGTWA*SIFSWHYATPFLLLTLIGLMFVSLYGRWETA LGFGLVFGLLYDIVYTDLIGI
WP_07403554 5.1	Potassium/proton antiporter	1	25	MPVPVISTDVLILLFGLLLVAGVMA*TRISTRFGLPALILFMGIGMIMGS DITGLIFFDDSNLAQLIGVAA
WP_07403763 8.1	Alpha-amylase	1	21	MRRGVVLVLLSLLLFPTVVGA*KEAATWEQERMYFIMVDRFVDGNP DNNEQVDKDDPKAFHGGDIRGIIEK
WP_07403775 9.1	Nitrous oxidase accessory-like protein	1	19	MKRILSCILFFCCFTQVEA*EEHSIPLTEPIVIESNEHVDGDGKTYTSC GHPAFIMRGTGAVLERVSVQQC
WP_07403481	Iron-siderophore ABC	0.975	18	MKKWMLALLTLCLTVVLA*ACGGSDDTENDTSSETATRSIEHAMGT ADVPENPERVVVLTNEGTE
5.1	Transporter substrate-	2		ALLALG
	Binding protein			
WP_01272654	Transporter substrate-	0.969	21	MKKGFLALCLAGLAAFSVACG*ADVNEGGSTSTDGEDDKVIVMGTS ADYFPYEFVDTANGDEIVG
9.1	Binding domain-	7		FDIDIA
	Containing protein			
WP_07403775	ABC transporter	0.916	35	MSLWRMEWARSLRQRENYVFLVIWILTLVLLGGLG*QALPVAADYTN VSATLITVLGLLLPLFILL
7.1	Permease subunit	3		TTALH
WP_07403506	Molybdate ABC	0.842	22	MKRVSEVIVGVTVALLLSQVWS*SSDSEDTVTILAAASLGPALEAVER QLEAEFDGIDVRVVTNGS
1.1	Transporter substrate-	3		GALRA
	Binding protein			
WP_07403517	ABC transporter	0.842	16	MKKLMALLASLTLVLA*ACGNDTETQTEQANEPQTLKVASLIPPMTD MLEIAKEQLAEENIELEIVV
0.1	Substrate-binding	2		LGDN
	Protein			
WP_07403778	Multidrug efflux SMR	0.786	22	MSKEKSAWISLLFAGLLEIVWA*TTMKLSEGFTILGPTLWTIVLLILSFG LLAKAFRTLPAGTGYAVF
5.1	Transporter	2		TGI
WP_07403716	Peptidoglycan	0.712	52	MERSRVARRQDSTKKKRKKTGKQPKTKSKKPMWKRIFLLGFMLFIA AVIAFG*AYTVYAIATAPEL
4.1	Glycosyltransferase	8		DEDKL
WP_07403777	Isoprenylcysteine	0.688	21	MNWMDVLLIVVSFFWLVETWR*FRNRKEATDGAVERKSFYFVATTMI GVFVLSMISSLVFESQPST
6.1	Carboxyl methyltransfer	9		VQRII
	ase family protein			
WP_07403608	Type I pullulanase	0.684	29	MVKKQWKPVWAVVLTFALILSMFPMSTSA*LDKGKSTLVVVHYQEA PDNEKDWNLWLWANGPD
1.1				YFPNKGFT
WP_07403496 0.1	Na+/H+ antiporter NhaC	0.596 9	28	MQMKGKEAVFVVLMSGITLLALMFLAKA*SPHMAIFGTMLIVGGYAY YRSRDFKQIETAMINGIRE AIMPV
WP_07403765 0.1	Phytoene desaturase	0.568	21	MKQIVVIGAGLGGLSAAVTLA*ARGYDVTVIEKNQHVGGKLMPIITDG HRFDFGPNTITMPDVFRS VIRNS
WP_14318027 9.1	Type IV secretory system	0.562 7	27	MRTQSLSPRFPIRLIFPALFCLLVVPA*GIFYLLNGVYNIIRQIMTPIVQD GLLAGTTIPTLSPSLFLEVS

*Star in the peptide sequence represents the cleavage site.*

Apart from PGP traits, some proteins with the secondary metabolite pathway were also identified which were predicted to have SPs. These include isoprenylcysteine carboxyl methyltransferase family protein and phytoene desaturase (Table 5). The former is involved in the methyl-transferase activity of isoprenylcysteine (Clarke et al., 1988; Winter-Vann et al., 2005), and the latter one is involved in the terpene biosynthesis pathways by converting phytoene to carotene by desaturase activity (Cunningham and Gantt, 1998; Grünewald et al., 2000). Beside the secondary metabolite proteins, the pullulanase type I enzyme with predicted secretion was also identified (Table 5). Pullulanase type I (PulA) is a debranching enzyme that specifically cleaves α-1,6-glycosidic linkages in pullulan. Pullulan has not only diverse applications in the food industry but also immune-stimulatory effects on B and T cells and is found to enhance the production of various anti-inflammatory cytokines in human (Coltelli et al., 2020).

### Differential Expression of Gene Analysis Validates the Presence of Secretary and Some Unique Hypothetical Proteins

**FIGURE 4:**
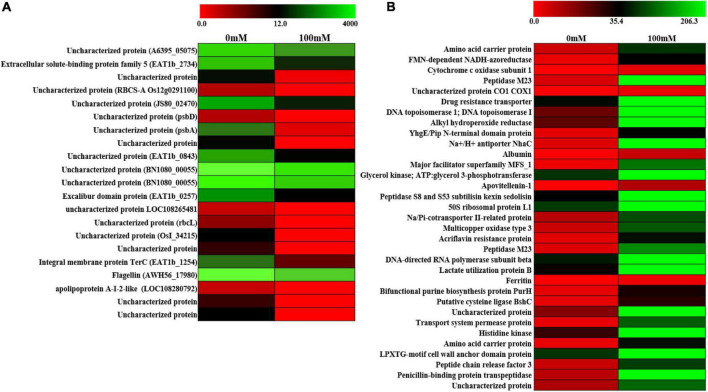
Differential expression of gene (DEG) analysis of PHM11. **(A)** Heatmap of the downregulated genes and **(B)** heatmap of the upregulated genes. Red color indicates no expression while green indicates highest expression.

In order to further look into the transcriptomic changes upon 100-mM salinity stress in PHM11, whole-cell transcriptomics was performed. A total of 2,320 transcripts were found, out of which 21 were downregulated (Figure 4A) and 33 were upregulated (Figure 4B and Supplementary Table S5). Most of the downregulated genes belonged to hypothetical genes whose functions could not be assigned. Some of the proteins identified belonged to extracellular solute-binding protein family 5, flagelline, and apolipoprotein A-I-2-like proteins. The upregulated gene belonged to amino acid carrier protein, cytochrome c oxidase, drug resistance transporter, Na^+^/H^+^ antiporter (NhaC), multicopper oxidase, acriflavin resistance protein, ferritin, bifunctional purine biosynthesis protein PurH, transport system permease protein, and penicillin-binding protein transpeptidase. Two hypothetical genes were also identified. The drug resistance transporter belonged to the region 9 of the biosynthetic cluster genes belonging to pseudaminic acid biosynthesis pathways (Knirel et al., 1997; Chou et al., 2005).

The upregulated genes were further classified through GO analysis for their functions (Supplementary Table S4). The amino acid carrier protein was predicted with SP with a 0.72 score (WP_074037007.1, Supplementary Table S3) which had four TMHs spanning to the plasma membrane (Supplementary Figure S2). The cytochrome c oxidase was related to the region 14 of the BGCs related to dipeptide aldehyde biosynthesis. These genes were related to the stress response (Dalle-Donne et al., 2009; Meyer et al., 2009; Gaballa et al., 2010). The Na^+^/H^+^ antiporter (NhaC) was also predicted with the SP having 10 TMH spanning regions (Figure 3F). The TMHs of the antiporter have a role in substrate transport (Diepold et al., 2015; Krampen et al., 2018).

### *Exiguobacterium profundum* PHM11 Inoculation Promotes the Plant Growth

**FIGURE 5:**
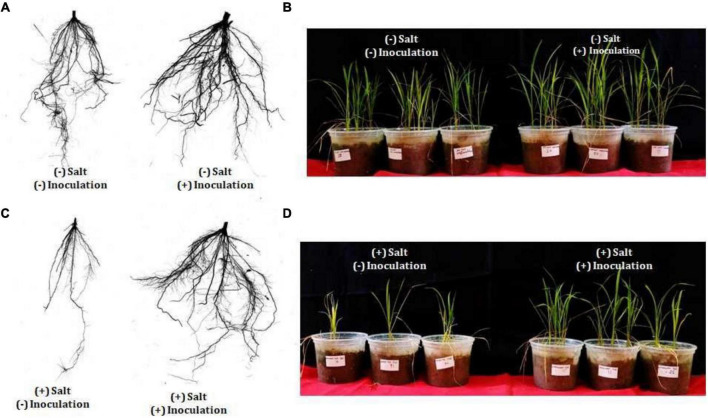
Physical appearance of the plant (rice) after 28 days of showing. **(A)** The left side of the figure represents the root of rice plant grown till 28 days in normal soil (no salt) grown from the untreated seed while the right side represents the root of wheat plant grown till 28 days in normal soil (no salt) grown from the treated seed (PMH11). **(B)** Represents the appearance of the whole plant shoot grown till 28 days in normal soil (no salt) grown from non-treated (left side) and treated (right side) seed. Similarly, **(C)** the left side of the figure represents the root of rice plant grown till 28 days in soil supplemented with 100 mM NaCl grown from the untreated seed while the right side represents the root of rice plant grown till 28 days in soil supplemented with 100 mM NaCl grown from the treated seed. **(D)** Represents the appearance of the whole plant shoot grown till 28 days in soil supplemented with 100 mM NaCl grown from non-treated (left side) and treated (right side) seed.

*Exiguobacterium profundum* was found positive for phosphate solubilization and zinc solubilization as well as siderophore, ammonia, and HCN production (Páez-Espino et al., 2009; Tang et al., 2013; White et al., 2013; Gutiérrez-Preciado et al., 2017). In addition to these PGP traits, we also investigated the presence of PK, dipeptide aldehydes, and NRP providing the environment suitability to the organism (Das and Khosla, 2009; Bushley and Turgeon, 2010; Wang et al., 2014; Martínez-Núñez and López, 2016; Chen et al., 2018). Because of the presence of all these traits, PHM11 was thought to promote plant growth. Its effect on the growth of paddy (*Oryza sativa* var. Rajendra sweta) was studied in pot trials under the influence of 100 mM salt (NaCl) (Table 6). The growth parameters were recorded after 28 days of sowing (Table 6 and Figure 5). The results showed that the roots of the treated plants had multiple branching and were healthier as compared to the non-treated plants (Figure 5A). The plant height was also improved in treated seeds (Figure 5B). Similar observations were found when the plants were grown in the soil having 100 mM of salt (NaCl). The plants without treatment had the compromised appearance of root (Figure 5C), plant height, and appearance (Figure 5D) as compared to that treated with PHM11. Furthermore, in absence of the salt when the seeds were pretreated with PHM11, the root length, root diameter, projected area, surface area, number of forks, number of links, and total chlorophyll content increased significantly (Table 6).

**TABLE 6:** Effect of *E. profundum* PHM11 inoculation on plant growth promotion and physiological attributes of rice.

Treatments Shoot length	Total root length (cm)	Projected area (cm^2^)	Surface area (cm^2^)	Average diameter (mm)	Root vol. (cm^3^)	No. of forks (m)	No. of links	Chlorophyll content
T1 a19.2 ± 3.0	^a^202.68 ± 5.57	^a^9.6 ± 0.28	^a^30.27 ± 1.04	^a^.465 ± 0.02	^a^0.33 ± 0.01	^a^1212 ± 25.45	^a^2510 ± 18.36	^a^11.93 ± 0.15
T2 ^a^15.93 ± 1.8	^b^171.40 ± 2.64	^c^6.08 ± 0.26	^c^20.53 ± 1.18	^b^.345 ± 0.03	^c^0.195 ± 0.02	^c^581 ± 22.62	^c^1274.5 ± 36.06	^c^8.43 ± 0.41
T3 a17.56 ± 0.58	^a^205.94 ± 8.23	^b^7.45 ± 0.47	^b^24.13 ± 0.49	^b^.38 ± 0.014	^b^0.255 ± 0.02	^b^833 ± 52.32	^b^2045 ± 62.22	^b^9.43 ± 0.41
T4 b12.33 ± 0.75	^c^57.82 ± 3.24	^d^2.21 ± 0.24	^d^6.74 ± 0.45	^b^.35 ± 0.021	^d^0.070 ± 0.01	^d^197 ± 5.65	^d^455 ± 18.38	^d^4.36 ± 0.37

*T1, plants treated with E. profundum PHM11 + 0 mM NaCl; T2, plants without treatment with E. profundum PHM11 + 0 mM NaCl; T3, plants treated with E. profundum PHM11 + 100 mM NaCl; and T4, plants without treatment with E. profundum PHM11 + 100 mM NaCl.*

*Means having the same letter within each variety do not differ significantly at the probability level 0.05 by Tukey (HSD).*

**FIGURE 6:**
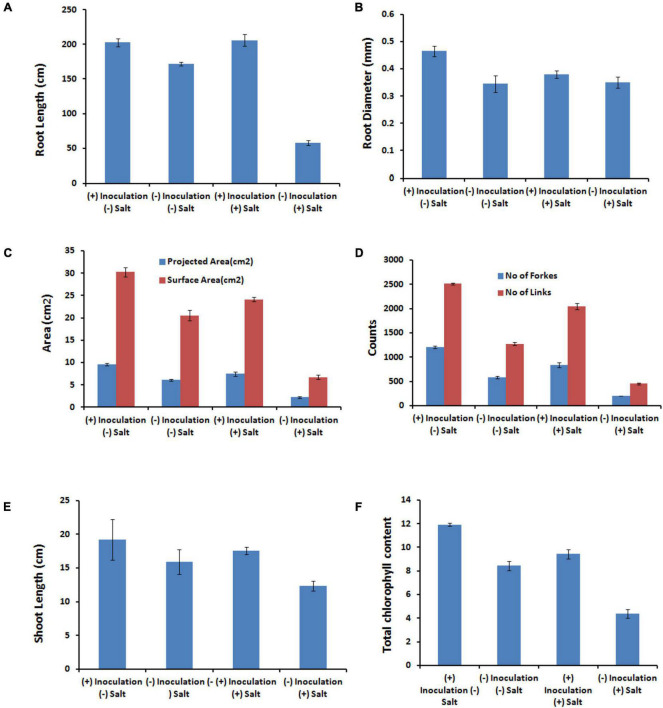
Statistical analysis of the several parameters. **(A)** Represents the root length, **(B)** represents the root diameters, **(C)** represents the projected and surface area, **(D)** represents the number of forks and links, **(E)** represents the shoot length, and **(F)** represents the total chlorophyll content in different conditions as represented in the figure like in the presence (100 mM NaCl) and absence of salt either treated with PHM11 or untreated.

The presence of salt leads to the compromised growth in the absence of PHM11 treatment, which was represented by the quantitative reduction in all measured parameters. Furthermore, the pretreatment of seeds with PHM11 was grown in 100 mM NaCl condition, PHM11 significantly enhanced the root length (Figure 6A), root diameter (Figure 6B), projected area (Figure 6C), surface area (Figure 6C), number of forks (Figure 6D), number of links (Figure 6D), shoot length (Figure 6E) and chlorophyll content (Figure 6F). Similar experiments were performed with wheat (Table 7) and maize (Table 8) as well. In case of wheat, we observed similar results. The growth of root and shoot was enhanced upon PHM11 treatment whether in the absence (Table 7 and Supplementary Figure S3A) or in the presence (Supplementary Figures S3A,B) of salt. Statistical analyses showed similar results for the root length, root diameter, projected area, surface area, number of forks, shoot length, and total chlorophyll content (Supplementary Figure S4). Similar results were obtained in the case of maize as well (Table 8 and Supplementary Figures S5, S6).

**TABLE 7:** Effect of *E. profundum* PHM11 inoculation on plant growth promotion and physiological attributes of wheat.

Treatments Shoot length	Total root length (cm)	Projected area (cm^2^)	Surface area (cm^2^)	Average root diameter (mm)	No. of forks (cm)	No. of links	Chlorophyll content
T1 a20.2 ± 1.9	^a^43.99 ± 0.26	^a^2.30 ± 0.42	^a^7.93 ± 0.33	^a^0.614 ± 0.04	^b^50.5 ± 0.70	^b^88.5 ± 0.70	^a^1.343 ± 0.07
T2 a,b15.66 ± 2.4	^c^28.74 ± 0.48	^a^1.28 ± 0.53	^c^5.05 ± 0.23	^a^0.495 ± 0.13	^d^5.5 ± 0.70	^d^20.5 ± 0.70	^b^1.096 ± 0.17
T3 a,b15.86 ± 0.98	^b^35.35 ± 0.64	^a^2.16 ± 0.28	^b^7.15 ± 0.19	^a^0.540 ± 0.04	^a^93.5 ± 3.5	^a^180.5 ± 0.70	^a,b^1.2 ± 0.01
T4 b 14.06 ± 3.8	^d^25.06 ± 0.79	^a^1.19 ± 0.24	^d^4.12 ± 0.20	^a^0.480 ± 0.07	^c^43 ± 1.40	^c^74. ± 1.40	^b^1.096 ± 0.075

*T1, plants treated with E. profundum PHM11 + 0 mM NaCl; T2, plants without treatment with E. profundum PHM11 + 0 mM NaCl; T3, plants treated with E. profundum PHM11 + 100 mM NaCl; and T4, plants without treatment with E. profundum PHM11 + 100 mM NaCl.*

*Means having the same letter within each variety do not differ significantly at the probability level 0.05 by Tukey (HSD).*

**TABLE 8:** Effect of *E. profundum* PHM11 inoculation on plant growth promotion and physiological attributes of maize.

Treatments	Shoot length	Total Root length(cm)	Projected Area(cm^2^)	Surface Area(cm^2^)	Average root diameter(mm)	No of Forkes (Cm)	No of Links	Chlorophyll content
T1	^a^35.6 ± 2.53	^a^173.2 ± 8.53	^a^14.65 ± 0.59	^a^45.705 ± 1.42	^a^1.20 ± 0.02	^a^665.5 ± 7.77	^a^1262 ± 55.15	^a^11.2233 ± 0.42
T2	^a^28.77 ± 11.1	^b^65.02 ± 5.51	^c^7.79 ± 0.46	^c^23.78 ± 0.48	^c^.87 ± 0.02	^b^234.5 ± 30.4	^c^437 ± 36.06	^b^8.2800 ± 0.14
T3	^a^35.16 ± 3.68	^a^157.45 ± 4.12	^b^13.04 ± 0.08	^b^42.33 ± 1.64	^b^1.12 ± 0.01	^a^610.5 ± 28.9	^b^1088 ± 9.89	^b^8.3267 ± 0.42
T4	^a^22.16 ± 7.38	^b^51.13 ± 3.89	^d^5.635 ± 0.33	^d^17.405 ± 0.6	^c^.88 ± 0.042	^d^52.5 ± 12.02	^d^90.5 ± .70	^c^7.0600 ± 0.13

*T1, plants treated with E. profundum PHM11 + 0 mM NaCl; T2, plants without treatment with E. profundum PHM11 + 0 mM NaCl; T3, plants treated with E. profundum PHM11 + 100 mM NaCl; and T4, plants without treatment with E. profundum PHM11 + 100 mM NaCl.*

*Means having the same letter within each variety do not differ significantly at the probability level 0.05 by Tukey (HSD).*

## Conclusion

The study exhibits the presence of many BGCs in the genome of *E. profundum* PHM11 which contributes toward environmental suitability through different metabolic pathways. PHM11 possesses biosynthetic pathways for several secondary metabolites like NRP, PK, dipeptide aldehydes, AMP, and carotenoid. Some putative bacteriocins with antibacterial activities were also identified. Besides, these varieties of proteins with the SPs as well as TMH which have a role in the transport and signaling were identified in the genome. The analysis of differential expression of genes (DEGs) showed the upregulation of some transporters like Na^+^/H^+^ antiporter (NhaC). These transporters and antiporters help them to sustain the high salt conditions. These transporters possess the TMH which form the channel through the membrane to transport the solutes across the membrane. In addition to the environmental suitability, the strain has PGP traits that remain functional under salt stress and make the strain a potential agent for the formulation for crops under salt-affected soil.

## Data Availability Statement

The datasets presented in this study can be found in online repositories. The names of the repository/repositories and accession number(s) can be found below: https://www.ncbi.nlm.nih.gov/, MRSV01000001.

## Author Contributions

AlSr participated in conceptualization, data curation, analysis, investigation, methodology, data validation, and writing of the original draft. RS and AB have done the formal analysis, validation of the data, and writing of the draft. AlSi performed the antagonistic activity of the strain. AS isolated the strain and performed genomic DNA isolation and basic characterization. SD performed the growth promotion experiments. PT and AnSr performed the preliminary analysis of results. PK, HC, and AnSa gave critical inputs. All authors contributed to the article and approved the submitted version.

## Conflict of Interest

The authors declare that the research was conducted in the absence of any commercial or financial relationships that could be construed as a potential conflict of interest.

## Publisher’s Note

All claims expressed in this article are solely those of the authors and do not necessarily represent those of their affiliated organizations, or those of the publisher, the editors and the reviewers. Any product that may be evaluated in this article, or claim that may be made by its manufacturer, is not guaranteed or endorsed by the publisher.

## Footnotes

1
https://www.ebi.ac.uk/Tools/msa/clustalo/

2
http://ggdc.gbdp.org

3
http://www.predisi.de/

4
http://www.cbs.dtu.dk/services/TMHMM/
