# Supplementary material for: Analysis of Biosynthetic Gene Clusters, Secretory, and Antimicrobial Peptides Reveals Environmental Suitability of Exiguobacterium profundum PHM11
Source: Front Microbiol. 2022 Feb 3;12:785458. doi: 10.3389/fmicb.2021.785458 (PMC8851196; doi:10.3389/fmicb.2021.785458)
Supplement: Supplementary file 1 [file Table_1.DOC]

**Supplementary information**

**Supplementary Table 1: Antagonistic activity of the *Exiguobacterium profundum* PHM11**

| **S.no** | **Microorganisms** | **Strain** | **NCBI Accession No** | ***Growth inhibition** |
| --- | --- | --- | --- | --- |
|  | **Bacteria** |  |  |  |
| 1 | *Bacillus pumilus* | Sikkm2127_10 | MZ359963 | + |
| 2 | *Paenibacillus terrae* | Sikkm2127_ 13 | MZ359966 | + |
| 3 | *Microbacterium foliorum* | Sikkim2127_15 | MZ359967 | +++ |
| 4 | *Priestia megaterium* | Sikkm2121_30 | MZ359970 | - |
| 5 | *Bacillus australimaris* | Sikkim2119_43 | MZ359973 | +++ |
| 6 | *Bacillus altitudinis* | Sikkim2127_88 | MZ359974 | - |
| 7 | *Bacillus megaterium* | Sikkim2101_95 | MZ359975 | ++ |
| 8 | *Burkholderia plantarii* | Sikkim2102_111 | MZ359977 | + |
| 9 | *Burkholderia gladioli* | Sikkim2102_113 | MZ359978 | ++ |
| 10 | *Alcaligenes faecalis* | Sikkim2101_63 | MZ411678 | + |
| 11 | *Paenibacillus polymyxa* | Sikkim2113_48 | MZ008227 | - |
| 12 | *Bacillus xiamenensis* | Sikkim2101_55 | MZ008232 | - |
| 13 | *Stenotrophomonas maltophilia* | Sikkim2101_59 | MZ008234 | + |
| 14 | *Bacillus wiedmannii* | Sikkim2101_62 | MZ008235 | + |
| 15 | *Bacillus wiedmannii* | Sikkim2101_68 | MZ008240 | - |
| 16 | *Arthrobacter pascens* | Sikkim2101_71 | MZ008242 | - |
| 17 | *Bacillus aryabhattai* | Sikkim2101_73 | MZ008244 | + |
| 18 | *Pseudomonas jessenii* | Sikkim2119_75 | MZ008245 | + |
| 19 | *Bacillus megaterium* | Sikkim2119_77 | MZ411502 | + |
| 20 | *Bacillus aryabhattai* | Sikkim2119_ 83 | MZ411503 | +++ |
| 21 | *Aerococcus viridans* | Sikkim2118_ 87 | MZ411504 | + |
| 22 | *Staphylococcus sp.* | Siikim2127_91 | MZ411506 | + |
| 23 | *Serratia marcescens* | Sikkim2102_93 | MZ411507 | + |
| 24 | *Burkholderia gladioli* | Sikkim2102_96 | MZ411508 | + |
| 25 | *Serratia marcescens* | Sikkim2102_98 | MZ411509 | - |
| 26 | *Serratia marcescens* | Sikkim2113_45 | MZ411512 | - |
| 27 | *Serratia marcescens* | Sikkim2102_104 | MZ411686 | - |
| 28 | *Microbacterium trichothecenolyticum* | Sikkim2119_84 | MZ411684 | + |
| 29 | *Bacillus thuringiensis* | Sikkim2102_100 | MZ411685 | + |
| 30 | *Alcaligenes faecalis* | Sikkim2102_114 | MZ411679 | ++ |
| 31 | *Bacillus aerius* | Sikkim2119_92 | MZ411681 | + |
| 32 | *Serratia marcescens* | Sikkim2102_105 | MZ411682 | - |
| 33 | *Bacillus altitudinis* | WBESNJ-4 | MT083903 | + |
| 34 | *Bacillus aerius* | WBERMJ-33 | MT089922 | + |
| 35 | *Ensifer adhaerens* | AS7B15 | MT640312 | ++ |
| 36 | *Paenibacillus alvei* | AS7B8 | MT640310 | + |
| 37 | *Pseudomonas paralactis* | RPB10 | MT772190 | + |
| 38 | *Pseudomonas aeruginosa* | RPB21 | MT772196 | ++ |
| 39 | *Pdeudomonas sp* | KC31 | KF733016 | ++ |
| 40 | *Bacillus megaterium* | SK7-B-9 | OK217264 | + |
| 41 | *Ochrobactrum anthropi* | OR31-B-43 | OK217265 | + |
| 42 | *Staphylococcus arlettae* | SK7-B-4 | OK217266 | + |
| 43 | *Pseudomonas stutzeri* | OR31-B-9 | OK217267 | + |
| 44 | *Bacillus aryabhattai* | OR32-B-10 | OK217268 | + |
| 45 | *Bacillus licheniformis* | SK7-B-10 | OK217269 | + |
| 46 | *Bacillus licheniformis* | OR31-B-53 | OK217270 | + |
| 47 | *Bacillus aryabhattai* | OR32-B-7 | OK217271 | +++ |
| 48 | *Alcaligenes faecalis* | OR31-B-41 | OK217272 | + |
| 49 | *Bacillus pumilus* | OR31-B-52 | OK217273 | + |
| 50 | *Arthrobacter citreus* | SK6L-B-1 | OK217274 | + |
| 51 | *odococcus qingshengii* | SK6L-B-8 | OK217275 | + |
| 52 | *Microbacterium arthrosphaerae* | OR31-B-50 | OK217276 | + |
| 53 | *Brevibacillus agri* | SK6L-B-2 | OK217277 | + |
| 54 | *Rhodococcus oryzae* | Sikkim2101_51 | MZ008229 | - |
| 55 | *Arthrobacter globiformis* | Sikkim2101_53 | MZ008230 | - |
| 56 | *Achromobacter kerstersii* | Sikkim 2101_65 | MZ008237 | - |
| 57 | *Arthrobacter gandavensis* | Sikkim 2101_50 | MZ008228 | - |
| 58 | *Rhodococcus hoagii* | Sikkim2121_29 | MZ359969 | - |
| 59 | *Bacillus subtilis* | Sikkim2127_11 | MZ359965 | - |
| 60 | *Bacillus amyloliquefaciens* | Sikkim2127_23 | MZ359968 | - |
| 61 | *Bacillus safensis* | Sikkim2127_5 | MZ411501 | - |
| 62 | *Lysinibacillus meyeri* | Sikkim2127_90 | MZ411510 | - |
| 63 | *Ochrobactrum ciceri* | Sikkim2102_99 | MZ411511 | - |
|  | **Fungus** |  |  |  |
| 1 | *Fusarium* sp. | Sikkim2118_1F | OK135779 | ++ |
| 2 | *Fusarium* sp. | Sikkim2118_1F-2 | OK135780 | + |
| 3 | *Fusarium* sp. | Sikkim2118_7F | OK135781 | + |
| 4 | *Fusarium* sp. | Sikkim2118_8F | OK135782 | + |
| 5 | *Fusarium* sp. | Sikkim2119_2F | OK135783 | + |
| 6 | *Fusarium* solani | Sikkim2119_4F | OK135784 | ++ |
| 7 | *Fusarium* solani | Sikkim2121_17F | OK135785 | ++ |
| 8 | *Fusarium* sp. | Sikkim2124_1F | OK135786 | + |
| 9 | *Fusarium* sp. | Sikkim2124_2F | OK135787 | + |
| 10 | *Aspergillus fumigatus* | Sikkim2124_3F | OK135788 | +++ |
| 11 | *Cunninghamella elegans* | Sikkim2128_2F | OK135789 | ++ |
| 12 | *Aspergillus ustus* | Sikkim2132_1F | OK135790 | + |
| 13 | *Fusarium solani* | Sikkim2132_2F | OK135791 | + |
| 14 | *Fusarium solani* | Sikkim2127_1F | OK135792 | + |
| 15 | *Aspergillus fumigatus* | Sikkim2127_4F | OK135793 | + |
| 16 | *Fusarium oxysporum* | Sikkim2132_F3 | OK135794 | + |
| 17 | *Fusarium solani* | Sikkim2119_3F | OK135795 | ++ |
| 18 | *Fusarium sp.* | Sikkim2128_1F | OK135796 | + |
| 19 | *Fusarium oxysporum* | Sikkim2129_1F | OK135797 | + |
| 20 | *Mortierella alpina* | NL1-F-3 | MT653331 | + |
| 21 | *Microdochium sp.* | UPOF1 / (NS2 (1) | OK300083 | + |
| 22 | *Fusarium fujikuroi* | UPGF1 / AS3 (1) | MZ733736 | + |
| 23 | *Orbillia foliicola* | (UPOF2) / NS1 (5) | OK300084 | ++ |
| 24 | *Curvularia lycopersici* | TNCL2 / TN8 (2) | OK300092 | ++ |
| 25 | *Cordyceps memorabilis* | UPCM1 / UV1 | OK300085 | ++ |
| 26 | *Fusarium oxysporum* | TNFO1 / TN1 (2) | OK300087 | + |
| 27 | *Fusarium oxysporum f.sp. lycopersici* | F-00889 | - | - |
| 28 | *Rhizoctonia solani* | PURS1 | - | - |
| 29 | *Macrophomina phaseolina* | MP2 | - | - |
| 30 | *Sclerotium rolfsii* | F-03053 | - | - |
| 31 | *Fusarium udum* | F-02845 | NIFK00000000 | - |

*against *E. profundum* PHM 11 by Well diffusion for bacteria and Volatile compound assay for fungi

+ (zone of inhibition upto 5mm in bacteria, Percentage growth inhibition (PGI) 10% to 30% in fungus); ++ (zone of inhibition 6mm to 10mm, PGI= 31% to 50%); +++ (zone of inhibition >11 mm, PGI = >50%); and - not showing antagonistic activity.

**Supplementary Figure S1:** Representative figures showing Antagonistic activity of *Exiguobacterium profundum* PHM11

*Bacillus aryabhattai* Sikkim2119_ 83 *Ensifer adhaerens* AS7B15 *Pseudomonas sp.* KC31 *Burkholderia gladioli* Sikkim2102_113

Sikkim2119_ 83


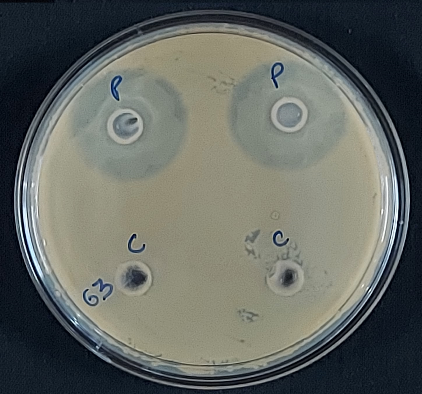

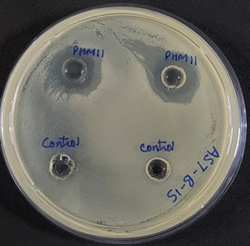

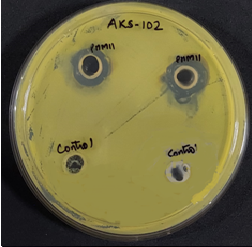

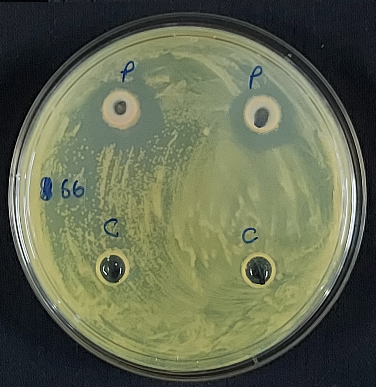

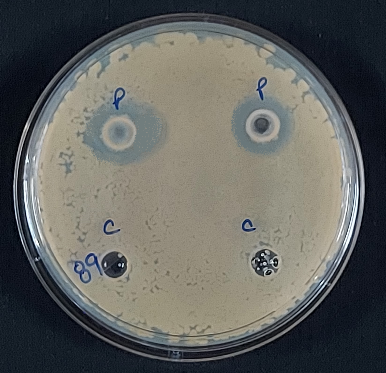

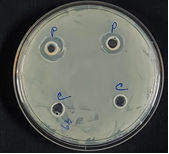

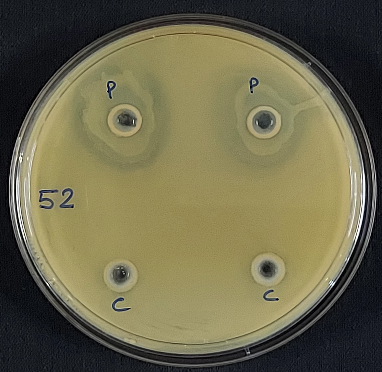

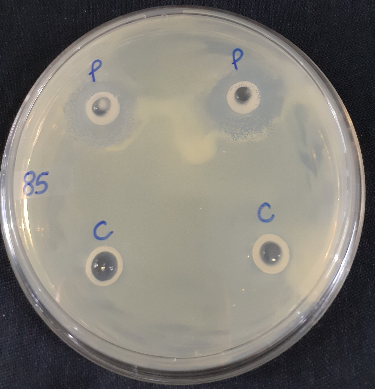


*Bacillus megaterium* Sikkim2101_95 *Alcaligenes faecalis* Sikkim2102_114 *Bacillus australimaris* Sikkim2119_43 *Microbacterium foliorum Sikkim2127_15*

Sikkim2119_ 83

*Aspergillus fumigatus* Sikkim2124_3F *Cunninghamella elegans* Sikkim2128_2F *Cordyceps memorabilis* UV1

*Fusarium sp*Sikkim2118_8F *Fusarium* solaniSikkim2119_4F *Curvularia lycopersici*TN8 (2)


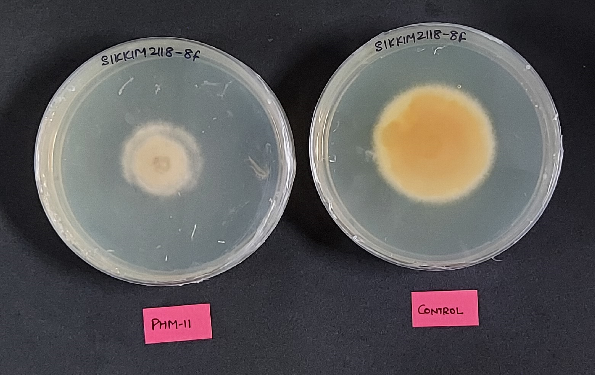

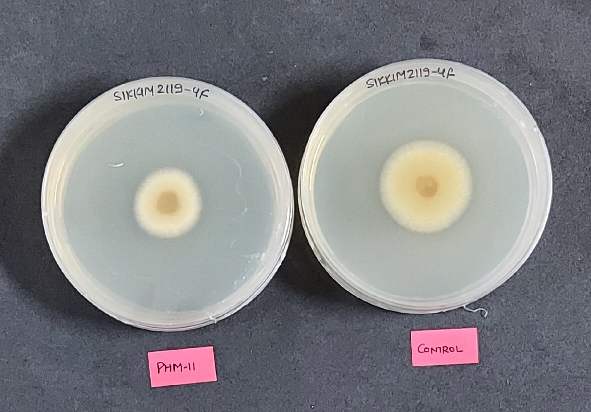

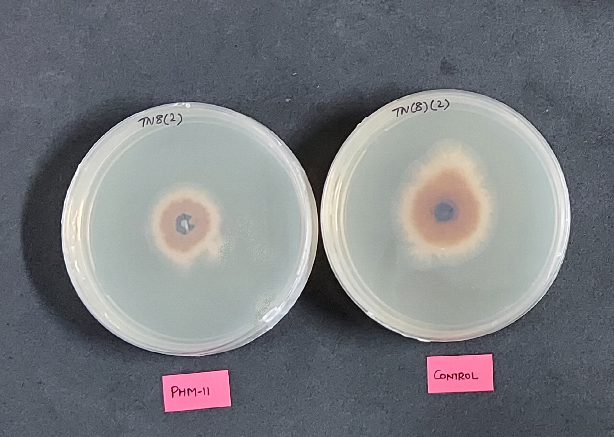

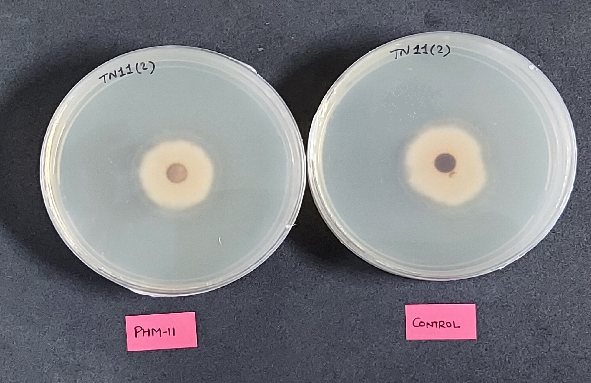

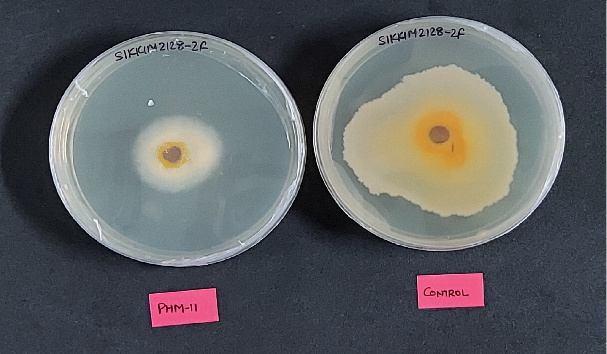

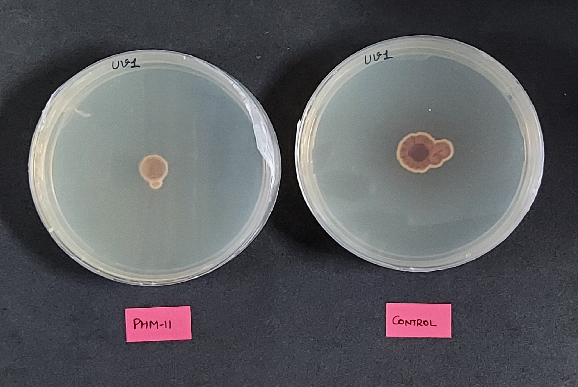


*Aspergillus fumigatus* Sikkim2124_3F *Cunninghamella elegans* Sikkim2128_2F *Cordyceps memorabilis* UV1

*Fusarium sp* Sikkim2118_8F *Fusarium* solaniSikkim2119_4F *Curvularia lycopersici*TN8 (2)


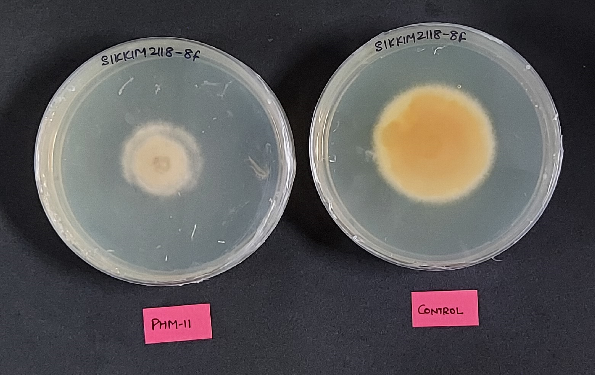

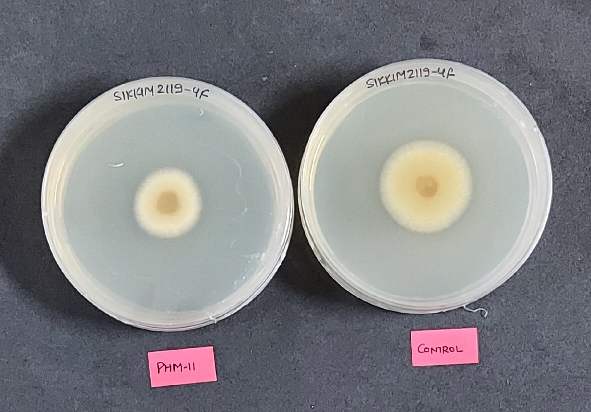

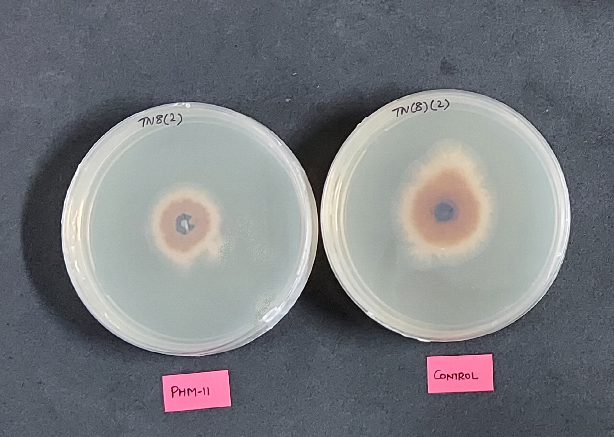

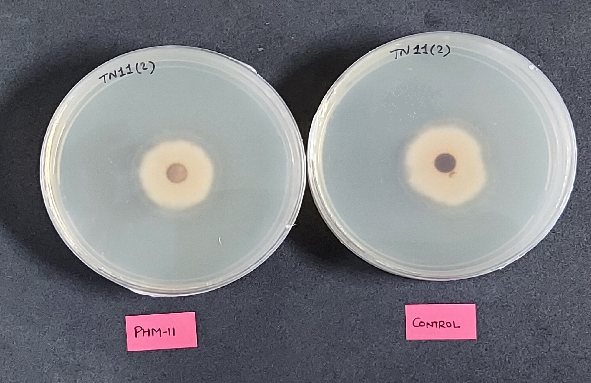

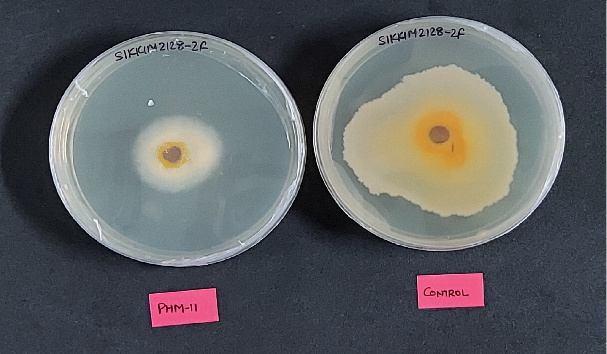

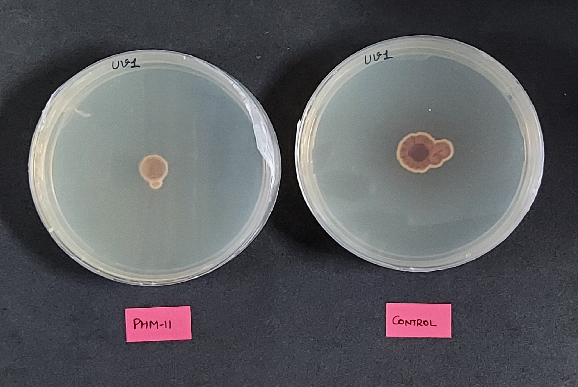

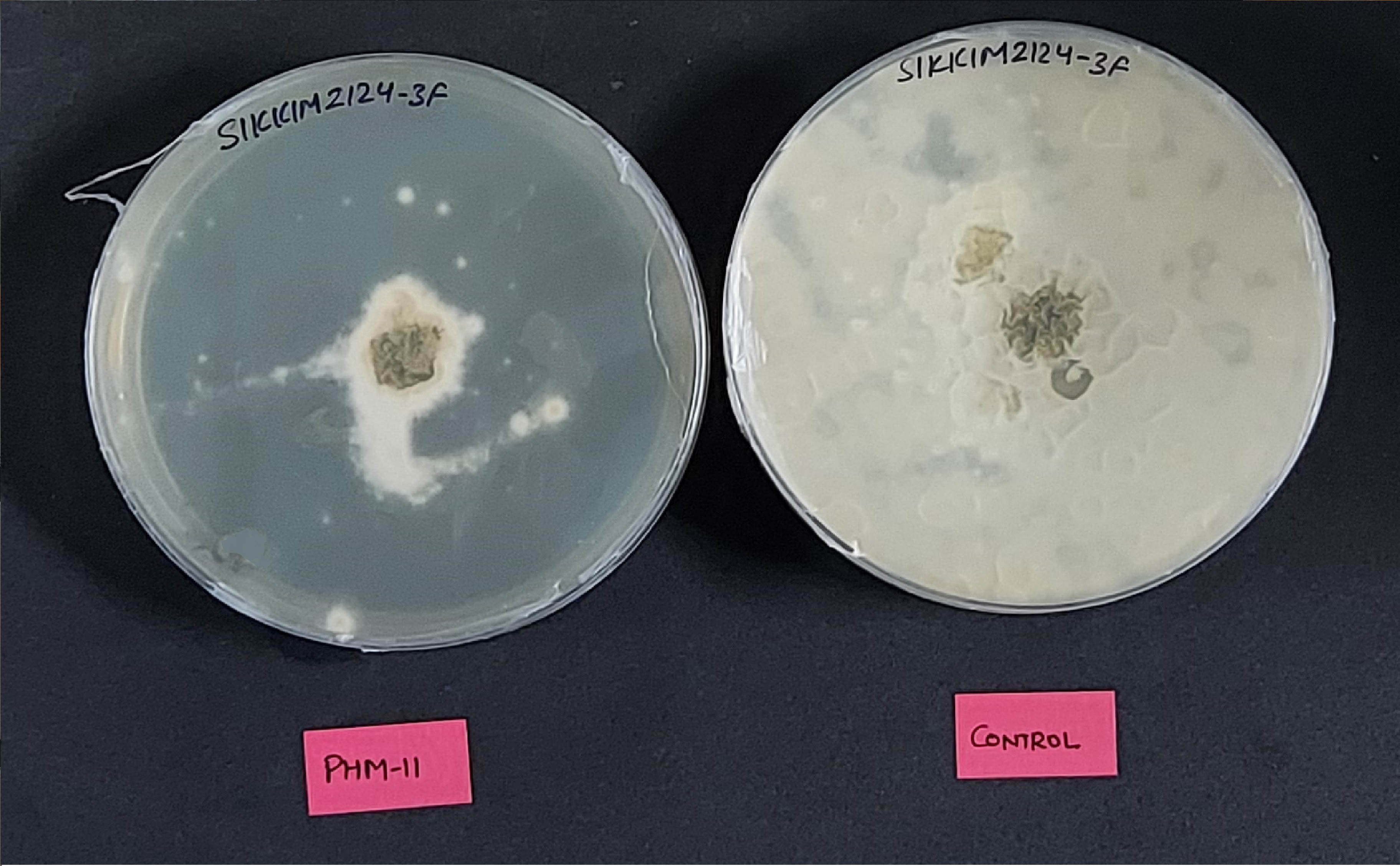


**
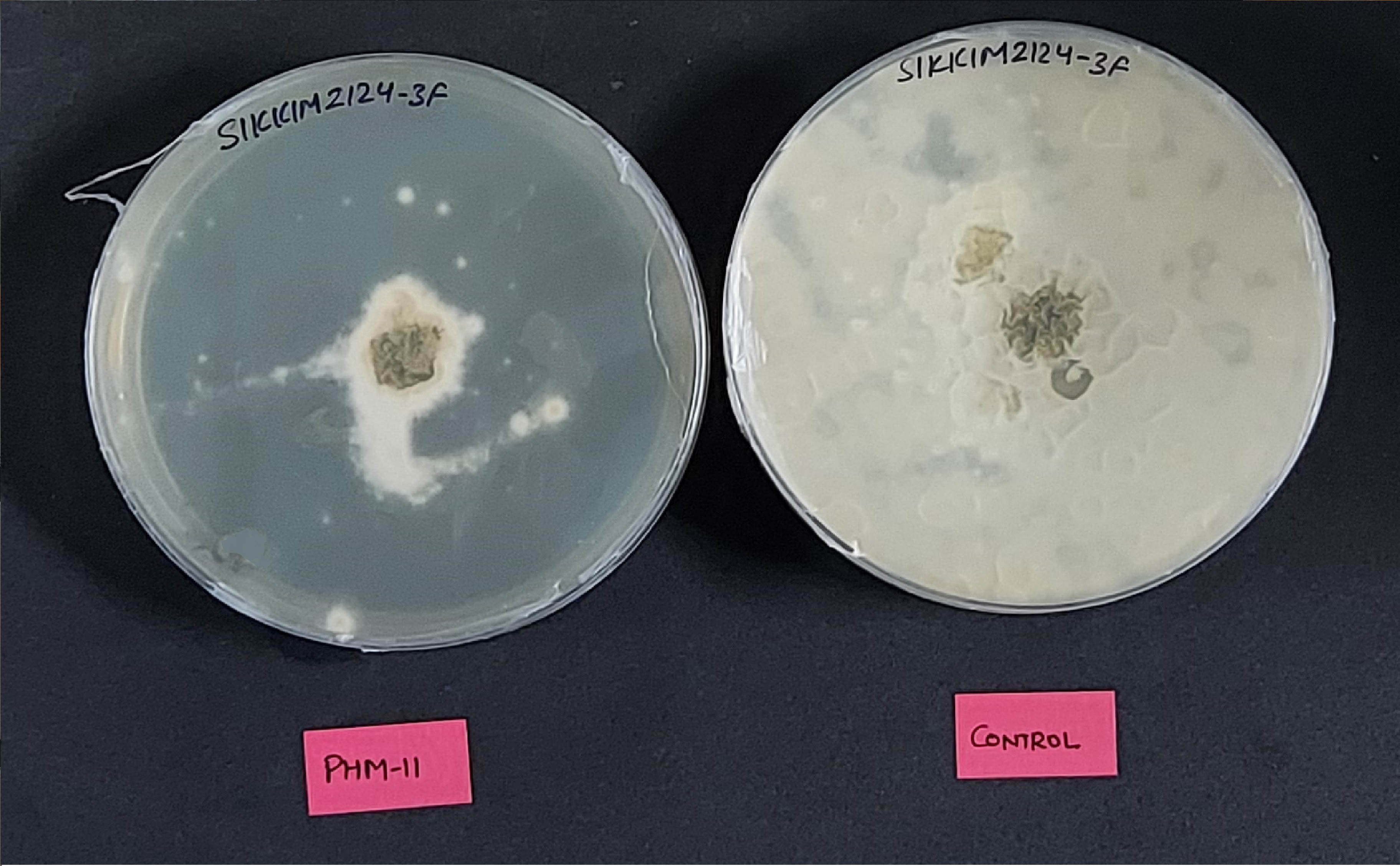
**

**Supplementary figure S2:** The transmembrane domains of amino acid carrier protein.

**
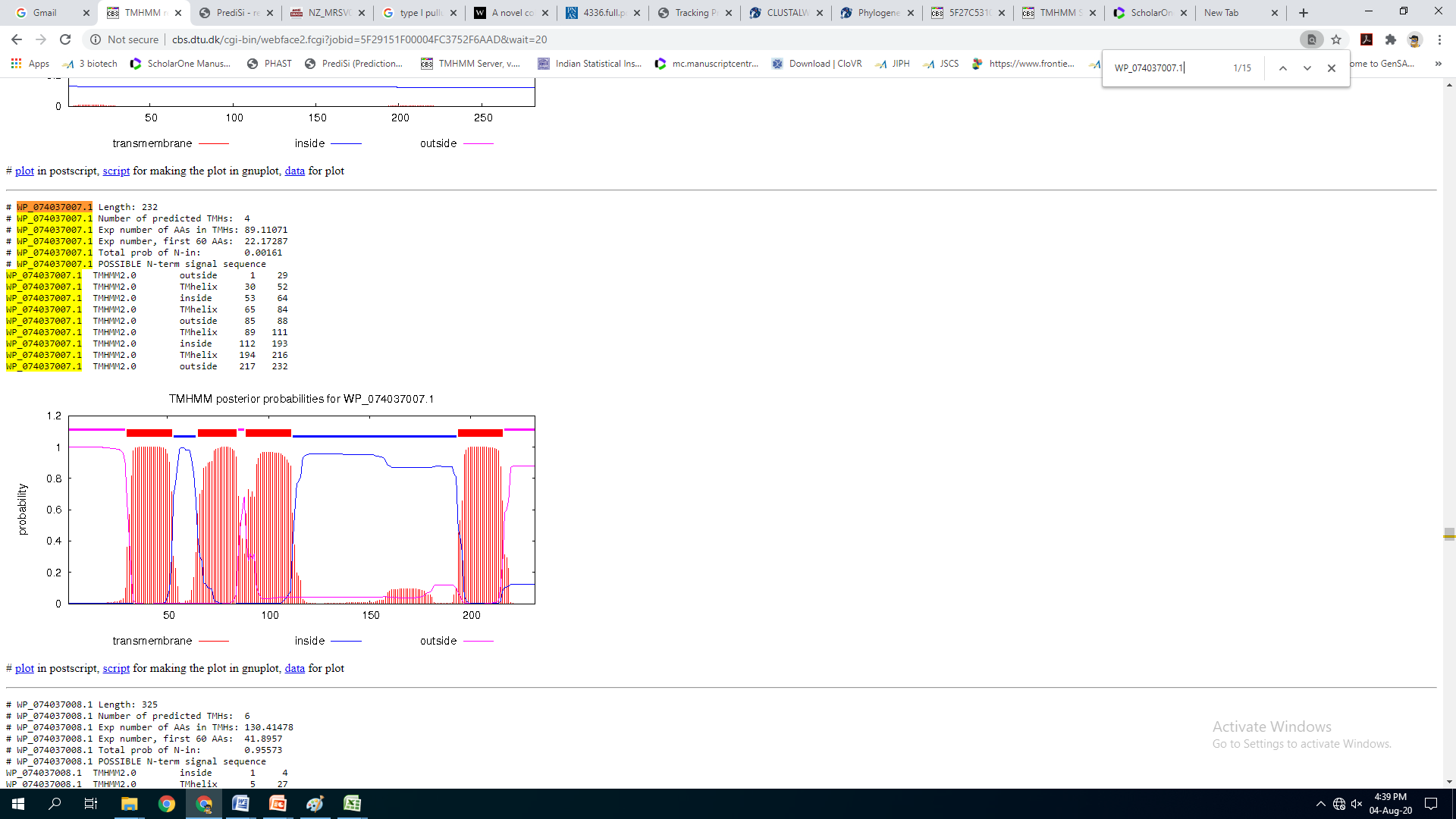
**

**Supplementary figure S3: Physical appearance of the plant (wheat) after 28 days of sowing.** (A) The left side of the figure represents the root of wheat plant grown till 28-days in normal soil (no salt) grown from the untreated seed while the right side represents the root of wheat plant grown till 28 days in normal soil (no salt) grown from the treated seed (PMH11). (B) Represent the appearance of the whole plant shoot grown till 28 days in normal soil (no salt) grown from non-treated (left side) and treated (right side) seed. Similarly, (C) the left side of the figure represents the root of wheat plant grown till 28 days in soil supplemented with 10mM NaCl grown from the untreated seed while the right side represents the root of wheat plant grown till 28 days in soil supplemented with 100 mM NaCl grown from the treated seed. (D) Represent the appearance of the whole plant shoot grown till 28 days in soil supplemented with 100 mM NaCl grown from non-treated (left side) and treated (right side) seed.

**
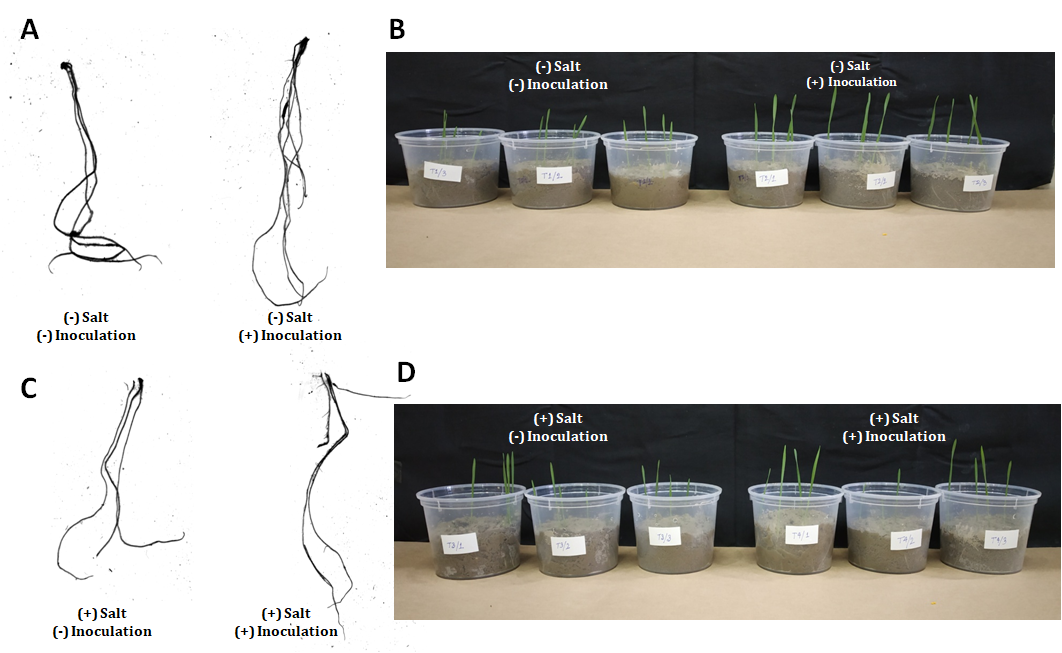
**

**Supplementary figure S4: Statistical analysis of the several parameters of the wheat.** (A) represents the root length, (B) represent the root diameters, (C) represents the projected and surface area, (D) represent the number of forks and links, (E) represent the shoot length and (F) represents the total chlorophyll content in different condition as represent in the figure like in presence (100mM NaCl) and absence of salt either treated with the PHM11 or untreated.

**
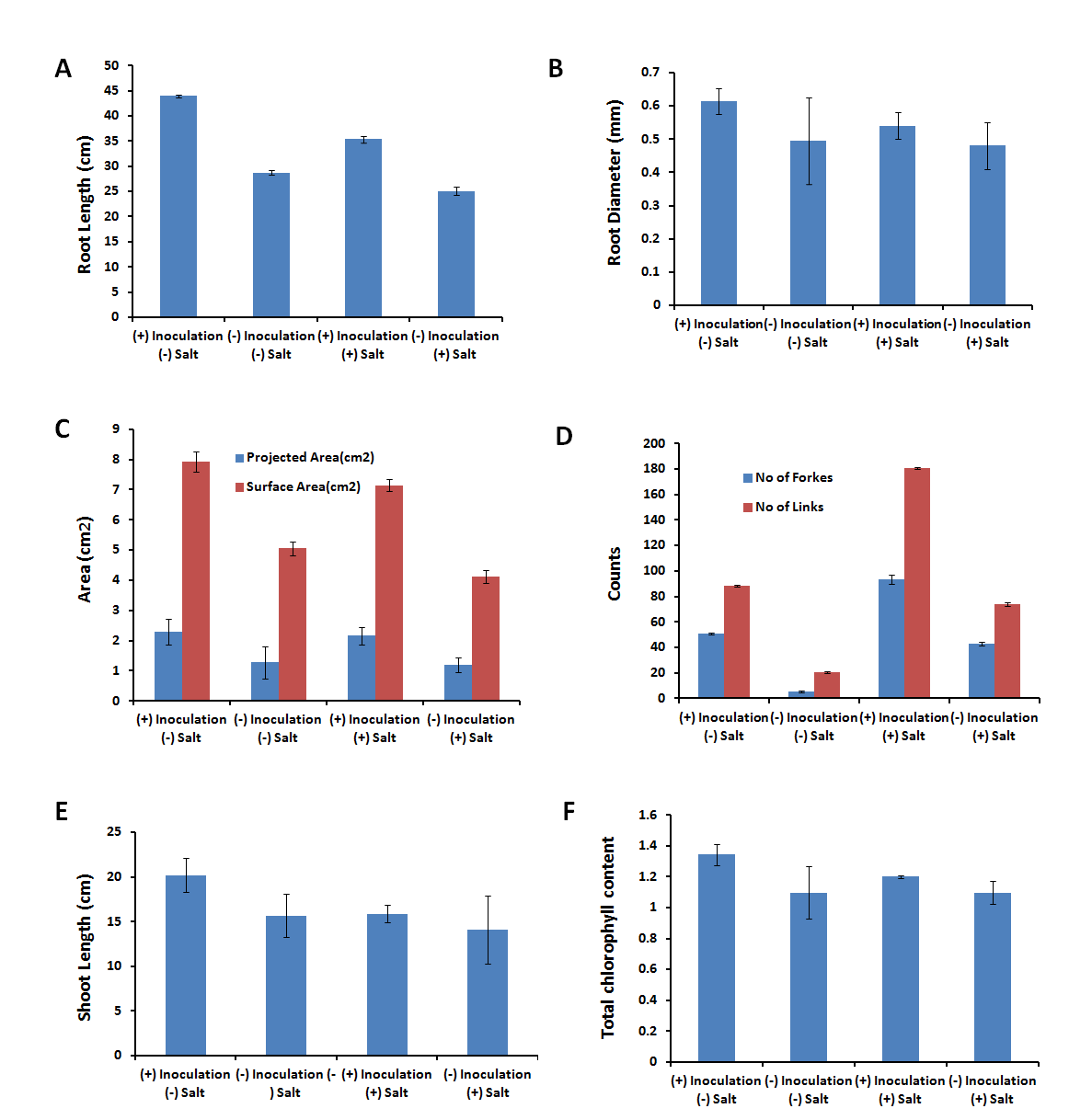
**

**Supplementary figure S5: Physical appearance of the plant (maize) after 28 days of sowing.** (A) The left side of the figure represents the root of maize plant grown till 28-days in normal soil (no salt) grown from the untreated seed while the right side represents the root of maize plant grown till 28 days in normal soil (no salt) grown from the treated seed (PMH11). (B) Represent the appearance of the whole plant shoot grown till 28 days in normal soil (no salt) grown from non-treated (left side) and treated (right side) seed. Similarly, (C) the left side of the figure represents the root of maize plant grown till 28 days in soil supplemented with 10mM NaCl grown from the untreated seed while the right side represents the root of maize plant grown till 28 days in soil supplemented with 100 mM NaCl grown from the treated seed. (D) Represent the appearance of the whole plant shoot grown till 28 days in soil supplemented with 100 mM NaCl grown from non-treated (left side) and treated (right side) seed.

**
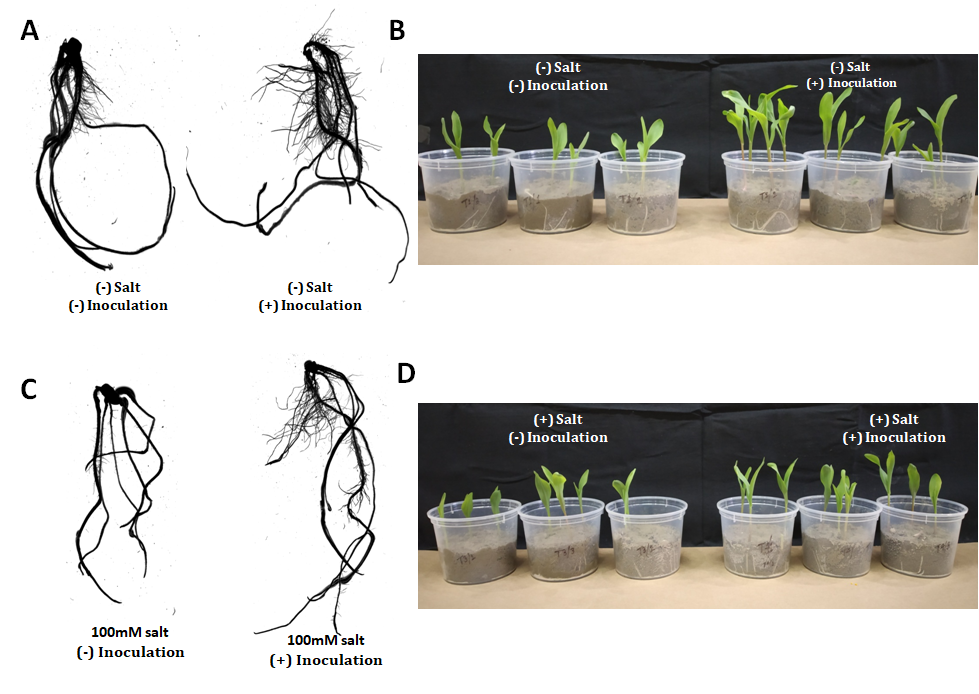
**

**Supplementary figure S6:** Statistical analysis of the several growth parameters of the maize plant.

**
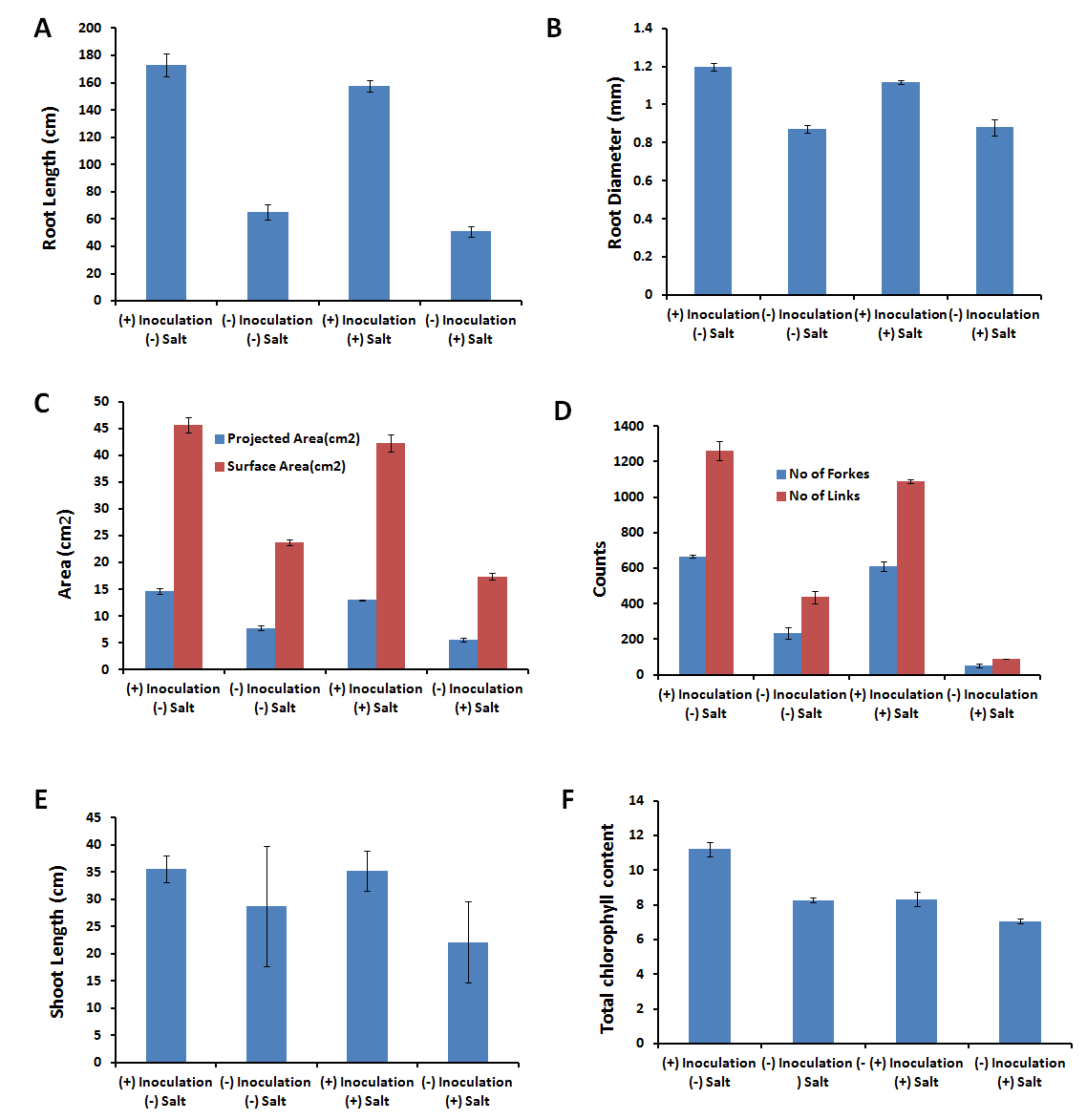
**

**Supplementary Table 2:** List of identified genes in 17 different BCGs using antiSMASH version 5.2.1.

| **Table of genes** | **Location** | | **Strand** | **Annotations of query cluster** |
| --- | --- | --- | --- | --- |
| **Region 1** | | | | |
| BBB58_RS00825 | 137875 | 138181 | - | rhodanese-like domain-containing protein |
| BBB58_RS00830 | 138458 | 138725 | + | metal-sensitive transcriptional regulator |
| BBB58_RS00835 | 138792 | 139224 | + | YbjN domain-containing protein |
| BBB58_RS00840 | 139315 | 140263 | + | DUF4003 domain-containing protein |
| BBB58_RS00845 | 140259 | 140982 | - | YwiC-like family protein |
| BBB58_RS00850 | 141090 | 141273 | - | hypothetical protein |
| BBB58_RS00855 | 141396 | 142029 | - | YitT family protein |
| BBB58_RS00860 | 142277 | 144401 | - | MMPL family transporter |
| BBB58_RS00865 | 144492 | 145293 | - | hypothetical protein |
| BBB58_RS00870 | 145423 | 145588 | + | YycC family protein |
| BBB58_RS00875 | 145602 | 147600 | - | type I pullulanase |
| BBB58_RS00880 | 147668 | 148160 | + | YajQ family cyclic di-GMP-binding protein |
| BBB58_RS00885 | 148156 | 148735 | + | metallophosphoesterase family protein |
| BBB58_RS00890 | 148778 | 150459 | - | arginine--tRNA ligase |
| BBB58_RS00895 | 150882 | 152562 | + | GGDEF domain-containing protein |
| BBB58_RS00900 | 152815 | 154108 | + | Na+/H+ antiporter NhaC family protein |
| BBB58_RS00905 | 154125 | 154770 | - | VTT domain-containing protein |
| BBB58_RS00910 | 154995 | 155937 | + | rhodanese-related sulfurtransferase |
| BBB58_RS00915 | 156055 | 156481 | + | divergent PAP2 family protein |
| **Region 2** | | | | |
| BBB58_RS02385 | 442587 | 442803 | + | S4 domain-containing protein YaaA |
| BBB58_RS02390 | 442804 | 443923 | + | DNA replication/repair protein RecF |
| BBB58_RS02395 | 443972 | 445901 | + | DNA topoisomerase (ATP-hydrolyzing) subunit B |
| BBB58_RS02400 | 445923 | 448491 | + | DNA gyrase subunit A |
| BBB58_RS02405 | 448548 | 449538 | + | metal-dependent phosphohydrolase |
| BBB58_RS02410 | 450079 | 451543 | + | IMP dehydrogenase |
| BBB58_RS02415 | 451583 | 455006 | - | Ig-like domain-containing protein |
| BBB58_RS02420 | 455146 | 455767 | - | class A sortase |
| BBB58_RS02425 | 455763 | 457167 | - | PLP-dependent aminotransferase family protein |
| BBB58_RS02430 | 457455 | 458346 | + | pyridoxal 5'-phosphate synthase lyase subunit PdxS |
| BBB58_RS02435 | 458347 | 458926 | + | pyridoxal 5'-phosphate synthase glutaminase subunit PdxT |
| BBB58_RS02440 | 459011 | 460259 | + | D-alanyl-D-alanine carboxypeptidase |
| BBB58_RS02445 | 460537 | 461821 | + | serine--tRNA ligase |
| BBB58_RS02455 | 462330 | 463002 | - | deoxynucleoside kinase |
| BBB58_RS02460 | 462998 | 463634 | - | deoxynucleoside kinase |
| BBB58_RS02465 | 463755 | 464256 | + | nucleoside deaminase |
| BBB58_RS02475 | 464671 | 466348 | + | DNA polymerase III subunit gamma/tau |
| BBB58_RS02480 | 466362 | 466680 | + | YbaB/EbfC family nucleoid-associated protein |
| BBB58_RS02485 | 466702 | 467302 | + | recombination protein RecR |
| BBB58_RS02490 | 467298 | 467523 | + | YaaL family protein |
| BBB58_RS02505 | 472896 | 474315 | + | aminotransferase class I/II-fold pyridoxal phosphate-dependent enzyme |
| BBB58_RS02510 | 474318 | 474957 | + | dTMP kinase |
| BBB58_RS02515 | 474956 | 475286 | + | cyclic-di-AMP receptor |
| BBB58_RS02520 | 475300 | 476293 | + | DNA polymerase III subunit delta' |
| BBB58_RS02525 | 476299 | 477130 | + | stage 0 sporulation family protein |
| BBB58_RS02530 | 477153 | 477489 | + | DNA replication initiation control protein YabA |
| BBB58_RS02535 | 477549 | 478287 | + | tRNA1(Val) (adenine(37)-N6)-methyltransferase |
| BBB58_RS02540 | 478279 | 478558 | + | GIY-YIG nuclease family protein |
| BBB58_RS02545 | 478529 | 479399 | + | 16S rRNA (cytidine(1402)-2'-O)-methyltransferase |
| BBB58_RS02550 | 479415 | 479709 | - | AbrB/MazE/SpoVT family DNA-binding domain-containing protein |
| BBB58_RS02555 | 480040 | 482002 | + | methionine--tRNA ligase |
| BBB58_RS02560 | 482053 | 482821 | + | TatD family hydrolase |
| BBB58_RS02565 | 482817 | 484110 | + | 3D domain-containing protein |
| **Region 3** | | | | |
| BBB58_RS03435 | 626715 | 628281 | + | diguanylate cyclase |
| BBB58_RS03440 | 628323 | 628773 | + | OsmC family protein |
| BBB58_RS03445 | 628835 | 629279 | + | GNAT family N-acetyltransferase |
| BBB58_RS03450 | 629275 | 629722 | - | DUF4385 domain-containing protein |
| BBB58_RS03455 | 629796 | 630339 | + | GNAT family N-acetyltransferase |
| BBB58_RS03460 | 630446 | 631424 | + | LacI family DNA-binding transcriptional regulator |
| BBB58_RS03465 | 631427 | 632312 | + | ribokinase |
| BBB58_RS03470 | 632311 | 632701 | + | D-ribose pyranase |
| BBB58_RS03475 | 632712 | 634191 | + | sugar ABC transporter ATP-binding protein |
| BBB58_RS03480 | 634192 | 635158 | + | ribose ABC transporter permease |
| BBB58_RS03485 | 635217 | 636093 | + | ribose ABC transporter substrate-binding protein RbsB |
| BBB58_RS03490 | 636147 | 637167 | - | cupin domain-containing protein |
| BBB58_RS03495 | 637575 | 638199 | - | arylformamidase |
| BBB58_RS03500 | 638214 | 639054 | - | tryptophan 2,3-dioxygenase |
| BBB58_RS03505 | 639067 | 640342 | - | kynureninase |
| BBB58_RS03510 | 640508 | 641357 | + | Cof-type HAD-IIB family hydrolase |
| BBB58_RS15125 | 641926 | 642091 | + | hypothetical protein |
| BBB58_RS03515 | 642116 | 643961 | - | ABC-F family ATP-binding cassette domain-containing protein |
| BBB58_RS03520 | 644289 | 645189 | + | DMT family transporter |
| BBB58_RS03525 | 645232 | 645424 | - | hypothetical protein |
| BBB58_RS03530 | 645437 | 646343 | - | Gfo/Idh/MocA family oxidoreductase |
| BBB58_RS03535 | 646406 | 647129 | - | anti-sigma factor |
| BBB58_RS03540 | 647125 | 647686 | - | sigma-70 family RNA polymerase sigma factor |
| BBB58_RS15040 | 647813 | 647999 | + | hypothetical protein |
| BBB58_RS03550 | 648084 | 648885 | + | HAD hydrolase family protein |
| BBB58_RS03555 | 648830 | 649136 | - | hypothetical protein |
| BBB58_RS03560 | 649291 | 649507 | - | hypothetical protein |
| BBB58_RS03565 | 649628 | 649862 | + | hypothetical protein |
| BBB58_RS03575 | 650210 | 651668 | + | UDP-N-acetylmuramoyl-L-alanyl-D-glutamate--2, 6-diaminopimelate ligase |
| BBB58_RS03580 | 651764 | 652109 | + | general stress protein |
| BBB58_RS03585 | 652176 | 653172 | - | oxidoreductase |
| BBB58_RS03590 | 653315 | 653738 | + | hypothetical protein |
| BBB58_RS03595 | 653756 | 653957 | - | hypothetical protein |
| BBB58_RS03600 | 654048 | 654756 | - | SDR family NAD(P)-dependent oxidoreductase |
| BBB58_RS03605 | 654767 | 655157 | - | GNAT family N-acetyltransferase |
| BBB58_RS03610 | 655153 | 656170 | - | serine hydrolase |
| BBB58_RS03615 | 656549 | 657242 | + | diguanylate cyclase |
| BBB58_RS03620 | 657262 | 659041 | - | Solute : sodium symporter family transporter |
| BBB58_RS03625 | 659155 | 660514 | - | magnesium transporter |
| BBB58_RS03630 | 660835 | 662065 | + | arginine deiminase |
| BBB58_RS03635 | 662086 | 663115 | + | ornithine carbamoyltransferase |
| BBB58_RS03640 | 663167 | 664643 | + | arginine-ornithine antiporter |
| **Region 4** | | | | |
| BBB58_RS03835 | 705264 | 706416 | + | S-methyl-5-thioribose kinase |
| BBB58_RS03840 | 706412 | 707447 | + | S-methyl-5-thioribose-1-phosphate isomerase |
| BBB58_RS03845 | 707439 | 708519 | + | sugar ABC transporter substrate-binding protein |
| BBB58_RS03850 | 708518 | 709892 | + | sugar ABC transporter ATP-binding protein |
| BBB58_RS03855 | 709893 | 710895 | + | ABC transporter permease |
| BBB58_RS03860 | 710909 | 711392 | - | GNAT family N-acetyltransferase |
| BBB58_RS03865 | 711467 | 711848 | + | VOC family protein |
| BBB58_RS03870 | 711876 | 712185 | - | helix-turn-helix transcriptional regulator |
| BBB58_RS03875 | 712303 | 712795 | + | lactoylglutathione lyase family protein |
| BBB58_RS03880 | 712797 | 713778 | + | zinc-binding dehydrogenase |
| BBB58_RS03885 | 713833 | 714892 | + | DegT/DnrJ/EryC1/StrS family aminotransferase |
| BBB58_RS03890 | 714935 | 715436 | - | SRPBCC domain-containing protein |
| BBB58_RS03895 | 715432 | 715765 | - | winged helix-turn-helix transcriptional regulator |
| BBB58_RS03900 | 715827 | 718119 | - | bifunctional glutamate--cysteine ligase GshA/glutathione synthetase GshB |
| BBB58_RS03905 | 718102 | 719431 | - | MATE family efflux transporter |
| BBB58_RS03910 | 719560 | 720028 | - | hypothetical protein |
| BBB58_RS03915 | 720024 | 720234 | - | helix-turn-helix transcriptional regulator |
| BBB58_RS03920 | 720353 | 721286 | + | ring-cleaving dioxygenase |
| BBB58_RS03925 | 721308 | 721749 | - | Rrf2 family transcriptional regulator |
| BBB58_RS03930 | 721935 | 723036 | + | oxidoreductase FAD/NAD(P)-binding domain-containing protein |
| BBB58_RS03935 | 723077 | 723494 | - | hypothetical protein |
| BBB58_RS03940 | 723704 | 724118 | - | DUF350 domain-containing protein |
| BBB58_RS03945 | 724114 | 725344 | - | glutathionylspermidine synthase family protein |
| BBB58_RS03950 | 725340 | 725709 | - | hypothetical protein |
| BBB58_RS03955 | 725705 | 726164 | - | hypothetical protein |
| BBB58_RS03960 | 726270 | 727290 | + | potassium channel family protein |
| BBB58_RS03965 | 727301 | 728429 | + | HD domain-containing protein |
| BBB58_RS03970 | 728558 | 728891 | + | PadR family transcriptional regulator |
| BBB58_RS03975 | 728887 | 729415 | + | DUF2812 domain-containing protein |
| BBB58_RS03980 | 729587 | 731132 | + | alpha-amylase |
| BBB58_RS03985 | 731333 | 732347 | + | fatty acid desaturase |
| BBB58_RS03990 | 732382 | 733213 | - | lipoate--protein ligase family protein |
| BBB58_RS03995 | 733230 | 734166 | - | lipoyl synthase |
| BBB58_RS04000 | 734454 | 736404 | + | glycoside hydrolase family 3 C-terminal domain-containing protein |
| BBB58_RS04005 | 736445 | 737291 | - | DegV family protein |
| BBB58_RS04010 | 737299 | 738142 | - | lipoate--protein ligase family protein |
| BBB58_RS04015 | 738249 | 739545 | + | HD domain-containing protein |
| **Region 5** | | | | |
| BBB58_RS04070 | 749334 | 749874 | + | TVP38/TMEM64 family protein |
| BBB58_RS04075 | 749849 | 751370 | - | cardiolipin synthase |
| BBB58_RS04080 | 751434 | 751785 | - | hypothetical protein |
| BBB58_RS04085 | 751850 | 753035 | - | chloride channel protein |
| BBB58_RS04090 | 753210 | 754149 | - | EAL domain-containing protein |
| BBB58_RS04095 | 754350 | 755190 | + | polyamine aminopropyltransferase |
| BBB58_RS04100 | 755186 | 756068 | + | agmatinase |
| BBB58_RS04105 | 756068 | 756428 | + | DUF2294 domain-containing protein |
| BBB58_RS04110 | 756646 | 757603 | + | sodium-dependent bicarbonate transport family permease |
| BBB58_RS04115 | 757659 | 758412 | - | hypothetical protein |
| BBB58_RS04120 | 758808 | 760719 | + | polysaccharide biosynthesis protein |
| BBB58_RS04125 | 760715 | 761744 | + | NAD-dependent epimerase |
| BBB58_RS04130 | 761743 | 763087 | + | phenylacetate--CoA ligase family protein |
| BBB58_RS04135 | 763104 | 764457 | + | phenylacetate--CoA ligase family protein |
| BBB58_RS04140 | 764477 | 765809 | + | nucleotide sugar dehydrogenase |
| BBB58_RS04145 | 765796 | 766927 | + | glycosyltransferase family 4 protein |
| BBB58_RS04150 | 766960 | 767596 | + | hypothetical protein |
| BBB58_RS04155 | 767714 | 768317 | + | hypothetical protein |
| BBB58_RS04160 | 768374 | 769139 | - | MerR family transcriptional regulator |
| BBB58_RS04165 | 769186 | 769651 | - | GNAT family N-acetyltransferase |
| BBB58_RS04170 | 769864 | 771568 | + | alpha-glucosidase |
| BBB58_RS04175 | 771686 | 772349 | + | Type 1 glutamine amidotransferase-like domain-containing protein |
| BBB58_RS04180 | 772383 | 772944 | + | GNAT family N-acetyltransferase |
| BBB58_RS04185 | 772995 | 773415 | + | DUF1934 domain-containing protein |
| BBB58_RS04190 | 773411 | 773837 | + | hypothetical protein |
| BBB58_RS04195 | 774007 | 774526 | + | GNAT family N-acetyltransferase |
| BBB58_RS04200 | 774533 | 775250 | + | hypothetical protein |
| BBB58_RS04205 | 775342 | 777469 | + | (Fe-S)-binding protein |
| BBB58_RS04210 | 777507 | 778806 | - | chemotaxis protein |
| BBB58_RS04215 | 778976 | 780302 | + | hypothetical protein |
| BBB58_RS04220 | 780382 | 780862 | + | DNA-directed RNA polymerase subunit delta |
| **Region 6** | | | | |
| BBB58_RS04860 | 902478 | 902865 | + | hypothetical protein |
| BBB58_RS04865 | 902984 | 903863 | - | manganese catalase family protein |
| BBB58_RS04870 | 904059 | 905586 | + | FAD-dependent oxidoreductase |
| BBB58_RS04875 | 905861 | 906218 | + | DUF2512 family protein |
| BBB58_RS04880 | 906274 | 906475 | + | hypothetical protein |
| BBB58_RS04885 | 906488 | 906785 | + | spore coat protein |
| BBB58_RS04890 | 906964 | 907822 | - | endo alpha-1,4 polygalactosaminidase |
| BBB58_RS04895 | 908018 | 909245 | + | hypothetical protein |
| BBB58_RS04900 | 909241 | 910156 | + | hypothetical protein |
| BBB58_RS04905 | 910152 | 911994 | + | DUF2194 domain-containing protein |
| BBB58_RS04910 | 911990 | 913409 | + | GT4 family glycosyltransferase PelF |
| BBB58_RS04915 | 913389 | 914850 | + | exopolysaccharide Pel transporter PelG |
| BBB58_RS04920 | 914891 | 915908 | + | UDP-glucose 4-epimerase GalE |
| BBB58_RS04925 | 916014 | 916629 | + | flavodoxin family protein |
| BBB58_RS04930 | 916666 | 917551 | - | decarboxylating 6-phosphogluconate dehydrogenase |
| BBB58_RS04935 | 917677 | 918556 | + | YihY/virulence factor BrkB family protein |
| BBB58_RS04940 | 918624 | 919548 | + | LCP family protein |
| BBB58_RS04945 | 919636 | 920842 | + | hypothetical protein |
| BBB58_RS04950 | 920848 | 921616 | + | protein tyrosine phosphatase |
| BBB58_RS04955 | 921837 | 922989 | + | ROK family transcriptional regulator |
| BBB58_RS04960 | 923155 | 923602 | + | hypothetical protein |
| BBB58_RS04965 | 923620 | 924928 | + | extracellular solute-binding protein |
| BBB58_RS04970 | 924995 | 925949 | + | sugar ABC transporter permease |
| BBB58_RS04975 | 925961 | 926747 | + | carbohydrate ABC transporter permease |
| BBB58_RS04980 | 926765 | 928754 | + | beta-galactosidase |
| BBB58_RS04985 | 928750 | 930817 | + | alpha-galactosidase |
| BBB58_RS04990 | 930854 | 932024 | + | galactokinase |
| BBB58_RS04995 | 932020 | 933025 | + | UDP-glucose 4-epimerase GalE |
| BBB58_RS05000 | 933021 | 934551 | + | UDP-glucose--hexose-1-phosphate uridylyltransferase |
| BBB58_RS05005 | 934565 | 935528 | + | galactose mutarotase |
| BBB58_RS05010 | 935587 | 937045 | - | 6-phospho-beta-glucosidase |
| BBB58_RS05015 | 937088 | 938990 | - | PTS glucose transporter subunit IIA |
| BBB58_RS05020 | 939387 | 940146 | + | hypothetical protein |
| BBB58_RS05025 | 940111 | 940810 | + | CpsD/CapB family tyrosine-protein kinase |
| BBB58_RS05030 | 940820 | 942035 | + | glycosyltransferase |
| BBB58_RS05035 | 942031 | 942958 | + | glycosyltransferase family 2 protein |
| BBB58_RS05040 | 942973 | 944020 | + | EpsG family protein |
| BBB58_RS05045 | 944038 | 945601 | + | oligosaccharide flippase family protein |
| BBB58_RS05050 | 945612 | 946764 | + | UDP-N-acetylglucosamine 2-epimerase (non-hydrolyzing) |
| BBB58_RS05055 | 946767 | 947406 | + | sugar transferase |
| BBB58_RS05060 | 947512 | 948556 | + | polysaccharide biosynthesis protein |
| BBB58_RS05065 | 948519 | 949797 | + | glycosyltransferase family 4 protein |
| BBB58_RS05070 | 949735 | 950962 | + | carbamoyl-phosphate-synthetase |
| BBB58_RS05075 | 950925 | 952038 | + | NAD-dependent epimerase/dehydratase family protein |
| BBB58_RS05080 | 952052 | 953030 | + | glycosyltransferase |
| BBB58_RS05085 | 953056 | 954178 | + | DegT/DnrJ/EryC1/StrS family aminotransferase |
| BBB58_RS05090 | 954174 | 955146 | + | glucose-1-phosphate thymidylyltransferase RfbA |
| BBB58_RS05095 | 955051 | 956104 | + | dTDP-glucose 4,6-dehydratase |
| BBB58_RS05100 | 956145 | 957126 | + | glycosyltransferase |
| BBB58_RS05105 | 957140 | 957839 | + | WbqC family protein |
| BBB58_RS05110 | 957887 | 958853 | + | glycosylase |
| BBB58_RS05115 | 958849 | 959788 | + | hypothetical protein |
| BBB58_RS05120 | 959787 | 960204 | + | GNAT family N-acetyltransferase |
| BBB58_RS15145 | 960349 | 960511 | - | hypothetical protein |
| BBB58_RS05130 | 960714 | 961956 | + | UDP-glucose/GDP-mannose dehydrogenase family protein |
| BBB58_RS05135 | 962053 | 962782 | + | DUF3784 domain-containing protein |
| BBB58_RS05140 | 962794 | 965491 | - | EAL domain-containing protein |
| **Region 7** | | | | |
| BBB58_RS05475 | 1034102 | 1034966 | - | tyrosine-type recombinase/integrase |
| BBB58_RS05480 | 1034980 | 1035424 | - | sigma-70 family RNA polymerase sigma factor |
| BBB58_RS05485 | 1035735 | 1037826 | + | alginate lyase family protein |
| BBB58_RS05490 | 1037940 | 1040031 | + | alginate lyase family protein |
| BBB58_RS05495 | 1040297 | 1042370 | + | 5'-nucleotidase C-terminal domain-containing protein |
| BBB58_RS05500 | 1042533 | 1045179 | + | glycosyltransferase family 2 protein |
| BBB58_RS05505 | 1045188 | 1045500 | + | hypothetical protein |
| BBB58_RS05510 | 1045542 | 1048608 | + | glycosyltransferase |
| BBB58_RS05515 | 1048604 | 1050788 | + | glycosyltransferase |
| BBB58_RS05520 | 1050915 | 1052205 | + | nucleotide sugar dehydrogenase |
| BBB58_RS05525 | 1052220 | 1053018 | + | ABC transporter permease |
| BBB58_RS05530 | 1053028 | 1053916 | + | teichoic acids export ABC transporter ATP-binding subunit TagH |
| BBB58_RS05535 | 1053965 | 1055066 | + | UDP-N-acetylglucosamine 2-epimerase (non-hydrolyzing) |
| BBB58_RS05540 | 1055147 | 1055624 | + | hypothetical protein |
| BBB58_RS05545 | 1055661 | 1057092 | + | MBOAT family protein |
| BBB58_RS05550 | 1057104 | 1058325 | + | hypothetical protein |
| BBB58_RS05555 | 1058365 | 1059679 | + | glycosyltransferase |
| BBB58_RS05560 | 1060192 | 1062604 | + | hypothetical protein |
| BBB58_RS05565 | 1062643 | 1062937 | - | hypothetical protein |
| BBB58_RS05570 | 1063122 | 1063632 | + | YdcF family protein |
| BBB58_RS05575 | 1063734 | 1065102 | + | zinc-ribbon domain-containing protein |
| BBB58_RS05580 | 1065113 | 1065539 | + | zinc ribbon domain-containing protein |
| BBB58_RS05585 | 1065535 | 1067233 | + | zinc ribbon domain-containing protein |
| BBB58_RS05590 | 1067272 | 1068013 | - | hypothetical protein |
| **Region 8** | | | | |
| BBB58_RS05665 | 1084529 | 1084943 | - | universal stress protein |
| BBB58_RS05670 | 1085013 | 1086486 | - | SulP family inorganic anion transporter |
| BBB58_RS05675 | 1086719 | 1087037 | + | winged helix-turn-helix transcriptional regulator |
| BBB58_RS05680 | 1087050 | 1087410 | + | arsenite efflux transporter metallochaperone ArsD |
| BBB58_RS05685 | 1087422 | 1089180 | + | arsenical pump-driving ATPase |
| BBB58_RS05690 | 1089176 | 1089455 | + | Fe-S cluster assembly protein HesB |
| BBB58_RS05695 | 1089487 | 1090774 | + | arsenic transporter |
| BBB58_RS05700 | 1090788 | 1092447 | + | FAD-dependent oxidoreductase |
| BBB58_RS05705 | 1092510 | 1093080 | + | hypothetical protein |
| BBB58_RS05710 | 1093104 | 1094241 | - | GGDEF domain-containing protein |
| BBB58_RS05715 | 1094419 | 1097341 | + | type I pullulanase |
| BBB58_RS05720 | 1097485 | 1097683 | + | hypothetical protein |
| BBB58_RS05725 | 1097757 | 1098000 | + | hypothetical protein |
| BBB58_RS05730 | 1098097 | 1099174 | + | nitric oxide synthase oxygenase |
| BBB58_RS05735 | 1099188 | 1099608 | + | RDD family protein |
| BBB58_RS05740 | 1099733 | 1100585 | + | DegV family protein |
| BBB58_RS05745 | 1100605 | 1100932 | + | thioredoxin family protein |
| BBB58_RS05750 | 1101018 | 1101420 | + | (4Fe-4S)-binding protein |
| BBB58_RS05755 | 1101431 | 1102328 | + | hypothetical protein |
| BBB58_RS05760 | 1102328 | 1102601 | + | N-acetyltransferase |
| BBB58_RS05765 | 1102616 | 1103699 | + | low temperature requirement protein A |
| BBB58_RS05770 | 1103729 | 1104455 | - | hypothetical protein |
| BBB58_RS05775 | 1104539 | 1106861 | - | FtsX-like permease family protein |
| **Region 9** | | | | |
| BBB58_RS06070 | 1161888 | 1162389 | + | PTS glucose transporter subunit IIA |
| BBB58_RS06075 | 1162479 | 1163079 | + | helix-turn-helix transcriptional regulator |
| BBB58_RS06080 | 1163145 | 1163571 | - | hypothetical protein |
| BBB58_RS06085 | 1163635 | 1164829 | - | ABC transporter permease |
| BBB58_RS06090 | 1164818 | 1165709 | - | ABC transporter ATP-binding protein |
| BBB58_RS06095 | 1165826 | 1166771 | + | VOC family protein |
| BBB58_RS06100 | 1166767 | 1167367 | + | alpha/beta hydrolase |
| BBB58_RS06105 | 1167372 | 1167861 | - | hypothetical protein |
| BBB58_RS06110 | 1168187 | 1169243 | + | acyltransferase family protein |
| BBB58_RS06115 | 1169317 | 1169653 | + | Asp23/Gls24 family envelope stress response protein |
| BBB58_RS06120 | 1169853 | 1170519 | + | beta-phosphoglucomutase |
| BBB58_RS06125 | 1170736 | 1171144 | + | hypothetical protein |
| BBB58_RS06130 | 1171266 | 1172319 | + | undecaprenyl/decaprenyl-phosphate alpha-N-acetylglucosaminyl 1-phosphate transferase |
| BBB58_RS06135 | 1172360 | 1173368 | - | LCP family protein |
| BBB58_RS06140 | 1173409 | 1174051 | - | YigZ family protein |
| BBB58_RS06145 | 1174227 | 1175355 | + | sensor histidine kinase |
| BBB58_RS06150 | 1175383 | 1176070 | + | response regulator |
| BBB58_RS06155 | 1176165 | 1177014 | + | DegV family protein |
| BBB58_RS06160 | 1177062 | 1177770 | + | trehalose operon repressor |
| BBB58_RS06165 | 1177972 | 1179421 | + | PTS system trehalose-specific EIIBC component |
| BBB58_RS06170 | 1179494 | 1181138 | + | alpha,alpha-phosphotrehalase |
| BBB58_RS06175 | 1181259 | 1182417 | + | DEAD/DEAH box helicase family protein |
| BBB58_RS06180 | 1182413 | 1183082 | + | ComF family protein |
| BBB58_RS06185 | 1183100 | 1183496 | + | flagellar protein |
| BBB58_RS06190 | 1183519 | 1183771 | + | flagellar biosynthesis anti-sigma factor FlgM |
| BBB58_RS06195 | 1183884 | 1184241 | + | flagellar export chaperone FlgN |
| BBB58_RS06200 | 1184253 | 1185744 | + | flagellar hook-associated protein FlgK |
| BBB58_RS06205 | 1185750 | 1186683 | + | flagellar hook-associated protein FlgL |
| BBB58_RS06210 | 1186689 | 1187265 | + | hypothetical protein |
| BBB58_RS06215 | 1187280 | 1187718 | + | flagellar assembly protein FliW |
| BBB58_RS06220 | 1187717 | 1187945 | + | carbon storage regulator CsrA |
| BBB58_RS06225 | 1188093 | 1189095 | + | GGDEF domain-containing protein |
| BBB58_RS06230 | 1189150 | 1190050 | - | YitT family protein |
| BBB58_RS06235 | 1190242 | 1191103 | + | YitT family protein |
| BBB58_RS06240 | 1191217 | 1191505 | + | antibiotic biosynthesis monooxygenase |
| BBB58_RS06245 | 1191607 | 1192543 | + | multidrug resistance efflux transporter family protein |
| BBB58_RS06250 | 1192579 | 1193077 | - | hypothetical protein |
| BBB58_RS06255 | 1193233 | 1193719 | + | hypothetical protein |
| BBB58_RS06260 | 1193779 | 1193971 | + | hypothetical protein |
| BBB58_RS06265 | 1194031 | 1194859 | + | GNAT family N-acetyltransferase |
| BBB58_RS06270 | 1194869 | 1195193 | + | hypothetical protein |
| BBB58_RS06275 | 1195315 | 1196608 | + | HAMP domain-containing protein |
| BBB58_RS06280 | 1196844 | 1197669 | + | flagellin |
| BBB58_RS06285 | 1197813 | 1199715 | + | DUF115 domain-containing protein |
| BBB58_RS06290 | 1199774 | 1200740 | + | UDP-N-acetylglucosamine 4,6-dehydratase (inverting) |
| BBB58_RS06295 | 1200781 | 1201954 | + | UDP-4-amino-4, 6-dideoxy-N-acetyl-beta-L-altrosamine transaminase |
| BBB58_RS06300 | 1201963 | 1202695 | + | glycosyltransferase family protein |
| BBB58_RS06305 | 1202724 | 1203696 | + | hypothetical protein |
| BBB58_RS06310 | 1203710 | 1204553 | + | methionyl-tRNA formyltransferase |
| BBB58_RS06315 | 1204582 | 1205626 | + | pseudaminic acid synthase |
| BBB58_RS15175 | 1205759 | 1206110 | + | flagellar protein |
| BBB58_RS06325 | 1206131 | 1207781 | + | flagellar filament capping protein FliD |
| BBB58_RS06330 | 1207795 | 1208170 | + | flagellar export chaperone FliS |
| BBB58_RS06335 | 1208166 | 1208520 | + | flagellar protein FliT |
| BBB58_RS06340 | 1208554 | 1208932 | - | DUF3021 family protein |
| BBB58_RS06345 | 1209091 | 1209277 | + | hypothetical protein |
| BBB58_RS06350 | 1209420 | 1210047 | + | hypothetical protein |
| BBB58_RS06355 | 1210196 | 1210670 | - | GyrI-like domain-containing protein |
| BBB58_RS06360 | 1210768 | 1211269 | - | DUF1572 family protein |
| **Region 10** | | | | |
| BBB58_RS06570 | 1254424 | 1254691 | + | glutaredoxin family protein |
| BBB58_RS06575 | 1254800 | 1255829 | + | hypothetical protein |
| BBB58_RS06580 | 1255888 | 1256896 | + | type I glyceraldehyde-3-phosphate dehydrogenase |
| BBB58_RS06585 | 1256993 | 1258178 | + | phosphoglycerate kinase |
| BBB58_RS06590 | 1258196 | 1258949 | + | triose-phosphate isomerase |
| BBB58_RS06595 | 1258949 | 1260488 | + | 2,3-bisphosphoglycerate-independent phosphoglycerate mutase |
| BBB58_RS06600 | 1260524 | 1261817 | + | phosphopyruvate hydratase |
| BBB58_RS06605 | 1262055 | 1262853 | + | aminoglycoside phosphotransferase family protein |
| BBB58_RS06610 | 1263085 | 1263967 | + | UTP--glucose-1-phosphate uridylyltransferase GalU |
| BBB58_RS06615 | 1263979 | 1265197 | + | glycosyltransferase family 4 protein |
| BBB58_RS06620 | 1265193 | 1266534 | + | glycosyltransferase |
| BBB58_RS06625 | 1266581 | 1267469 | + | YitT family protein |
| BBB58_RS06630 | 1267542 | 1267767 | + | preprotein translocase subunit SecG |
| BBB58_RS06635 | 1267869 | 1268610 | + | carboxylesterase |
| BBB58_RS06640 | 1268625 | 1271031 | + | ribonuclease R |
| BBB58_RS06645 | 1271085 | 1271556 | + | SsrA-binding protein SmpB |
| BBB58_RS06655 | 1272075 | 1272219 | + | alcohol dehydrogenase |
| BBB58_RS06660 | 1272850 | 1273135 | + | hypothetical protein |
| BBB58_RS06665 | 1273140 | 1274115 | + | hypothetical protein |
| BBB58_RS06670 | 1274117 | 1275236 | + | hypothetical protein |
| BBB58_RS06675 | 1275988 | 1276174 | + | hypothetical protein |
| **Region 11** | | | | |
| BBB58_RS07005 | 1346447 | 1347107 | + | DUF429 domain-containing protein |
| BBB58_RS07010 | 1347117 | 1348251 | - | sporulation integral membrane protein YtvI |
| BBB58_RS07015 | 1348330 | 1349110 | + | nucleotide pyrophosphohydrolase |
| BBB58_RS07020 | 1349236 | 1351150 | + | N-acetylmuramoyl-L-alanine amidase |
| BBB58_RS07025 | 1351230 | 1352271 | - | ribonucleotide-diphosphate reductase subunit beta |
| BBB58_RS07030 | 1352227 | 1352734 | - | flavodoxin domain-containing protein |
| BBB58_RS07035 | 1352783 | 1355024 | - | ribonucleoside-diphosphate reductase subunit alpha |
| BBB58_RS07040 | 1355571 | 1355802 | + | DUF1871 family protein |
| BBB58_RS07045 | 1355815 | 1356064 | + | NifU N-terminal domain-containing protein |
| BBB58_RS07050 | 1356060 | 1356861 | + | glycosyltransferase family 2 protein |
| BBB58_RS07055 | 1356863 | 1357358 | + | Lrp/AsnC family transcriptional regulator |
| BBB58_RS07060 | 1357354 | 1358539 | + | aminotransferase |
| BBB58_RS07065 | 1358597 | 1359842 | + | SidA/IucD/PvdA family monooxygenase |
| BBB58_RS07070 | 1360039 | 1360432 | + | general stress protein 13 |
| BBB58_RS07075 | 1360532 | 1361525 | + | Gfo/Idh/MocA family oxidoreductase |
| BBB58_RS07080 | 1361599 | 1362763 | + | iron-containing alcohol dehydrogenase |
| BBB58_RS07085 | 1362869 | 1364222 | + | glucose-6-phosphate isomerase |
| BBB58_RS07090 | 1364321 | 1364795 | + | nucleoside triphosphatase YtkD |
| BBB58_RS07095 | 1364775 | 1365540 | + | prolyl oligopeptidase family serine peptidase |
| BBB58_RS07100 | 1365555 | 1367127 | - | phosphoenolpyruvate carboxykinase (ATP) |
| **Region 12** | | | | |
| BBB58_RS07135 | 1372595 | 1373798 | + | MFS transporter |
| BBB58_RS07140 | 1373924 | 1374671 | + | bacteriorhodopsin |
| BBB58_RS07145 | 1374979 | 1377385 | + | leucine--tRNA ligase |
| BBB58_RS07150 | 1377496 | 1378630 | + | hypothetical protein |
| BBB58_RS07155 | 1378647 | 1379160 | + | hypothetical protein |
| BBB58_RS07160 | 1379334 | 1380312 | + | hypothetical protein |
| BBB58_RS07165 | 1380417 | 1381683 | - | NAD(P)/FAD-dependent oxidoreductase |
| BBB58_RS07170 | 1381826 | 1383524 | + | polysaccharide biosynthesis protein |
| BBB58_RS07175 | 1383520 | 1384237 | + | rRNA pseudouridine synthase |
| BBB58_RS07180 | 1384301 | 1384928 | - | DUF3885 domain-containing protein |
| BBB58_RS07185 | 1385031 | 1385253 | - | DeoR family transcriptional regulator |
| BBB58_RS07190 | 1385555 | 1386950 | + | dipeptidase PepV |
| BBB58_RS07195 | 1387009 | 1388161 | + | MFS transporter |
| BBB58_RS07200 | 1388166 | 1388742 | + | RNA 2',3'-cyclic phosphodiesterase |
| BBB58_RS07205 | 1388711 | 1389551 | + | NERD domain-containing protein |
| BBB58_RS07210 | 1389570 | 1391736 | + | type I pullulanase |
| BBB58_RS07215 | 1391797 | 1392580 | + | phosphotransferase |
| BBB58_RS07220 | 1392864 | 1393512 | + | tRNA (guanosine(46)-N7)-methyltransferase TrmB |
| BBB58_RS07225 | 1393611 | 1394127 | + | hypothetical protein |
| BBB58_RS07230 | 1394165 | 1394525 | - | PTS-dependent dihydroxyacetone kinase phosphotransferase subunit DhaM |
| BBB58_RS07235 | 1394526 | 1395117 | - | dihydroxyacetone kinase subunit L |
| BBB58_RS07240 | 1395136 | 1396114 | - | dihydroxyacetone kinase subunit DhaK |
| BBB58_RS07245 | 1396216 | 1396519 | - | PepSY domain-containing protein |
| BBB58_RS07250 | 1396657 | 1397731 | + | M42 family metallopeptidase |
| BBB58_RS07255 | 1397881 | 1398898 | + | PTS sugar transporter subunit IIC |
| BBB58_RS07260 | 1398932 | 1399232 | - | hypothetical protein |
| BBB58_RS07265 | 1399380 | 1399692 | + | thioredoxin family protein |
| BBB58_RS07270 | 1399698 | 1400493 | + | DUF1444 family protein |
| BBB58_RS07275 | 1400547 | 1401162 | + | DUF4479 domain-containing protein |
| **Region 13** | | | | |
| BBB58_RS09345 | 1800892 | 1801906 | - | D-alanyl-D-alanine carboxypeptidase |
| BBB58_RS09350 | 1802063 | 1803596 | + | FAD-dependent oxidoreductase |
| BBB58_RS09355 | 1803670 | 1805143 | + | carboxypeptidase M32 |
| BBB58_RS09360 | 1805139 | 1805847 | + | glycerophosphodiester phosphodiesterase |
| BBB58_RS09365 | 1806096 | 1806894 | + | DUF4397 domain-containing protein |
| BBB58_RS09370 | 1806967 | 1807483 | + | class F sortase |
| BBB58_RS09375 | 1807570 | 1808008 | - | MarR family transcriptional regulator |
| BBB58_RS09380 | 1808096 | 1809131 | - | (p)ppGpp synthetase |
| BBB58_RS09385 | 1809251 | 1809476 | + | hypothetical protein |
| BBB58_RS09390 | 1809555 | 1809876 | + | general stress protein |
| BBB58_RS09395 | 1809905 | 1810943 | - | SDR family oxidoreductase |
| BBB58_RS09400 | 1811132 | 1812428 | + | glutamine synthetase beta-grasp domain-containing protein |
| BBB58_RS09405 | 1812470 | 1813964 | - | peptide MFS transporter |
| BBB58_RS09410 | 1814107 | 1815352 | - | MFS transporter |
| BBB58_RS09415 | 1815620 | 1816721 | - | AI-2E family transporter |
| BBB58_RS09420 | 1817057 | 1818233 | + | aminotransferase class I/II-fold pyridoxal phosphate-dependent enzyme |
| BBB58_RS09425 | 1818229 | 1819246 | + | iron-containing alcohol dehydrogenase |
| BBB58_RS09430 | 1819465 | 1820287 | + | MetQ/NlpA family ABC transporter substrate-binding protein |
| **Region 14** | | | | |
| BBB58_RS10500 | 2025723 | 2026284 | - | hypothetical protein |
| BBB58_RS10505 | 2026364 | 2028989 | - | transglycosylase domain-containing protein |
| BBB58_RS10510 | 2029009 | 2029636 | - | Holliday junction resolvase RecU |
| BBB58_RS10515 | 2029992 | 2030844 | + | DUF3800 domain-containing protein |
| BBB58_RS10520 | 2030882 | 2031542 | - | endonuclease III |
| BBB58_RS10525 | 2031541 | 2032126 | - | DnaD domain protein |
| BBB58_RS10530 | 2032188 | 2033490 | - | asparagine--tRNA ligase |
| BBB58_RS10535 | 2033510 | 2034698 | - | pyridoxal phosphate-dependent aminotransferase |
| BBB58_RS10540 | 2034718 | 2035204 | - | hypothetical protein |
| BBB58_RS10545 | 2035200 | 2035386 | - | YpmA family protein |
| BBB58_RS10550 | 2035451 | 2038262 | - | ATP-dependent DNA helicase DinG |
| BBB58_RS10555 | 2038368 | 2038752 | - | aspartate 1-decarboxylase |
| BBB58_RS10560 | 2038757 | 2039588 | - | pantoate--beta-alanine ligase |
| BBB58_RS10565 | 2039584 | 2040421 | - | 3-methyl-2-oxobutanoate hydroxymethyltransferase |
| BBB58_RS10570 | 2040646 | 2041609 | - | biotin--[acetyl-CoA-carboxylase] ligase |
| BBB58_RS10575 | 2041581 | 2042721 | - | CCA tRNA nucleotidyltransferase |
| BBB58_RS10580 | 2042717 | 2043839 | - | N-acetyl-alpha-D-glucosaminyl L-malate synthase BshA |
| BBB58_RS10585 | 2043835 | 2044519 | - | bacillithiol biosynthesis deacetylase BshB1 |
| BBB58_RS10590 | 2044631 | 2045507 | + | YitT family protein |
| BBB58_RS10595 | 2045549 | 2046008 | - | GreA/GreB family elongation factor |
| BBB58_RS10600 | 2046072 | 2046756 | - | zinc metallopeptidase |
| BBB58_RS10605 | 2046794 | 2047412 | - | DUF1405 domain-containing protein |
| BBB58_RS10610 | 2047508 | 2048303 | - | c-type cytochrome |
| BBB58_RS10615 | 2048336 | 2049008 | - | cytochrome b6 |
| BBB58_RS10620 | 2049081 | 2049528 | - | ubiquinol-cytochrome c reductase iron-sulfur subunit |
| BBB58_RS10625 | 2049638 | 2050079 | - | DUF2487 family protein |
| BBB58_RS10630 | 2050239 | 2050773 | - | YpiB family protein |
| BBB58_RS10635 | 2050796 | 2052071 | - | tetratricopeptide repeat protein |
| BBB58_RS10640 | 2052100 | 2053351 | - | 3-phosphoshikimate 1-carboxyvinyltransferase |
| BBB58_RS10645 | 2053347 | 2054403 | - | 3-dehydroquinate synthase |
| **Region 15** | | | | |
| BBB58_RS11410 | 2205749 | 2206298 | - | DivIVA domain-containing protein |
| BBB58_RS11415 | 2206316 | 2207087 | - | RNA-binding protein |
| BBB58_RS11420 | 2207090 | 2207360 | - | YggT family protein |
| BBB58_RS11425 | 2207364 | 2207835 | - | cell division protein SepF |
| BBB58_RS11430 | 2207858 | 2208512 | - | YggS family pyridoxal phosphate-dependent enzyme |
| BBB58_RS11435 | 2208508 | 2209270 | - | peptidoglycan editing factor PgeF |
| BBB58_RS11440 | 2209321 | 2210464 | - | cell division protein FtsZ |
| BBB58_RS11445 | 2210510 | 2211797 | - | cell division protein FtsA |
| BBB58_RS11450 | 2212024 | 2212795 | - | FtsQ-type POTRA domain-containing protein |
| BBB58_RS11455 | 2212809 | 2213886 | - | undecaprenyldiphospho-muramoylpentapeptide beta-N-acetylglucosaminyltransferase |
| BBB58_RS11460 | 2213882 | 2215220 | - | UDP-N-acetylmuramoyl-L-alanine--D-glutamate ligase |
| BBB58_RS11465 | 2215233 | 2216193 | - | phospho-N-acetylmuramoyl-pentapeptide- transferase |
| BBB58_RS11470 | 2216214 | 2217519 | - | UDP-N-acetylmuramoyl-tripeptide--D-alanyl-D- alanine ligase |
| BBB58_RS11475 | 2217576 | 2219724 | - | penicillin-binding protein |
| BBB58_RS11480 | 2219723 | 2220086 | - | cell division protein FtsL |
| BBB58_RS11485 | 2220110 | 2221043 | - | 16S rRNA (cytosine(1402)-N(4))-methyltransferase RsmH |
| BBB58_RS11490 | 2221201 | 2222758 | - | bacillithiol biosynthesis cysteine-adding enzyme BshC |
| BBB58_RS11495 | 2222782 | 2223100 | - | DUF3397 family protein |
| BBB58_RS11500 | 2223135 | 2223978 | - | 2-dehydropantoate 2-reductase |
| BBB58_RS11505 | 2224094 | 2224268 | - | 50S ribosomal protein L32 |
| BBB58_RS11510 | 2224290 | 2224818 | - | DUF177 domain-containing protein |
| BBB58_RS11515 | 2224974 | 2226105 | + | nucleotidyltransferase family protein |
| **Region 16** | | | | |
| BBB58_RS11950 | 2305880 | 2306906 | - | ABC transporter permease |
| BBB58_RS11955 | 2306905 | 2307832 | - | ABC transporter permease |
| BBB58_RS11960 | 2308138 | 2309758 | - | peptide ABC transporter substrate-binding protein |
| BBB58_RS11965 | 2310027 | 2310396 | + | DUF3899 domain-containing protein |
| BBB58_RS11970 | 2310697 | 2311699 | + | tryptophan--tRNA ligase |
| BBB58_RS11975 | 2311802 | 2313458 | - | peptide ABC transporter substrate-binding protein |
| BBB58_RS11980 | 2313660 | 2315193 | - | peptide ABC transporter substrate-binding protein |
| BBB58_RS11985 | 2315259 | 2316492 | - | beta-ketoacyl-ACP synthase II |
| BBB58_RS11990 | 2316488 | 2317448 | - | ketoacyl-ACP synthase III |
| BBB58_RS11995 | 2317533 | 2318235 | - | 5'-methylthioadenosine/S-adenosylhomocysteine nucleosidase |
| BBB58_RS12000 | 2318289 | 2318763 | - | transcription elongation factor GreA |
| BBB58_RS12005 | 2318830 | 2319466 | - | uridine kinase |
| BBB58_RS12010 | 2319482 | 2320124 | - | O-methyltransferase |
| BBB58_RS12015 | 2320186 | 2321329 | - | endolytic transglycosylase MltG |
| BBB58_RS12020 | 2321397 | 2321694 | - | DUF1292 domain-containing protein |
| BBB58_RS12025 | 2321707 | 2322130 | - | Holliday junction resolvase RuvX |
| BBB58_RS12030 | 2322131 | 2322398 | - | IreB family regulatory phosphoprotein |
| BBB58_RS12035 | 2322435 | 2325078 | - | alanine--tRNA ligase |
| **Region 17** | | | | |
| BBB58_RS13015 | 2521625 | 2522738 | - | ribosome biogenesis GTPase YqeH |
| BBB58_RS13020 | 2522871 | 2523498 | - | YqeG family HAD IIIA-type phosphatase |
| BBB58_RS13025 | 2523623 | 2524403 | - | phosphatidylserine decarboxylase |
| BBB58_RS13030 | 2524399 | 2524822 | - | thioredoxin family protein |
| BBB58_RS13035 | 2524896 | 2525133 | - | DUF2929 family protein |
| BBB58_RS13040 | 2525250 | 2526066 | + | Cof-type HAD-IIB family hydrolase |
| BBB58_RS13045 | 2526115 | 2526964 | - | YitT family protein |
| BBB58_RS13050 | 2527110 | 2529408 | - | glycoside hydrolase family 65 protein |
| BBB58_RS13055 | 2529616 | 2530036 | - | general stress protein |
| BBB58_RS13060 | 2530151 | 2531210 | - | LacI family DNA-binding transcriptional regulator |
| BBB58_RS13065 | 2531265 | 2532768 | - | alpha-amylase |
| BBB58_RS13070 | 2532791 | 2533625 | - | sugar ABC transporter permease |
| BBB58_RS13075 | 2533626 | 2534979 | - | sugar ABC transporter permease |
| BBB58_RS13080 | 2535084 | 2536350 | - | extracellular solute-binding protein |
| BBB58_RS13090 | 2536720 | 2538505 | + | glycoside hydrolase family 13 protein |
| BBB58_RS13095 | 2538664 | 2540023 | + | MATE family efflux transporter |
| BBB58_RS13100 | 2540046 | 2540781 | - | enoyl-CoA hydratase/isomerase family protein |
| BBB58_RS13105 | 2540922 | 2541396 | + | Cys-tRNA(Pro) deacylase |
| BBB58_RS13110 | 2541415 | 2542114 | + | CDP-diacylglycerol--serine O-phosphatidyltransferase |
| BBB58_RS13115 | 2542203 | 2542953 | + | carotenoid biosynthesis protein |
| BBB58_RS13120 | 2542918 | 2543617 | + | 1-acyl-sn-glycerol-3-phosphate acyltransferase |
| BBB58_RS13125 | 2543613 | 2544681 | + | glycosyltransferase |
| BBB58_RS13130 | 2544707 | 2546174 | + | phytoene desaturase |
| BBB58_RS13135 | 2546616 | 2547456 | - | squalene/phytoene synthase family protein |
| BBB58_RS13140 | 2547424 | 2548903 | - | phytoene desaturase |
| BBB58_RS13145 | 2548899 | 2550393 | - | phytoene desaturase |
| BBB58_RS13150 | 2550462 | 2551494 | - | class I SAM-dependent methyltransferase |
| BBB58_RS13155 | 2551594 | 2551978 | - | YisL family protein |
| BBB58_RS13160 | 2552091 | 2554650 | - | GAF domain-containing protein |
| BBB58_RS13165 | 2554646 | 2555009 | - | response regulator |
| BBB58_RS13170 | 2555142 | 2556936 | - | ATP-binding cassette domain-containing protein |

**Supplementary Table 3:** List of protein with Signal peptide prediction using PrediSi server.

| **FASTA ID and name of the protein** | **Score** | **Cleavage Position** |
| --- | --- | --- |
| WP_049759982.1 MULTISPECIES: rod shape-determining protein MreD [Exiguobacterium] | 1 | 18 |
| WP_074035545.1 potassium/proton antiporter [Exiguobacterium profundum] | 1 | 25 |
| WP_074037638.1 alpha-amylase [Exiguobacterium profundum] | 1 | 21 |
| WP_074037759.1 nitrous oxidase accessory-like protein [Exiguobacterium profundum] | 1 | 19 |
| WP_074034815.1 iron-siderophore ABC transporter substrate-binding protein [Exiguobacterium profundum] | 0.9752 | 18 |
| WP_012726549.1 MULTISPECIES: transporter substrate-binding domain-containing protein [Exiguobacterium] | 0.9697 | 21 |
| WP_074035027.1 class F sortase [Exiguobacterium profundum] | 0.9558 | 23 |
| WP_074037894.1 hypothetical protein [Exiguobacterium profundum] | 0.9392 | 23 |
| WP_074036968.1 S8 family serine peptidase [Exiguobacterium profundum] | 0.9207 | 20 |
| WP_143180272.1 MULTISPECIES: DUF3397 family protein [Exiguobacterium] | 0.9199 | 60 |
| WP_074037757.1 ABC transporter permease subunit [Exiguobacterium profundum] | 0.9163 | 35 |
| WP_074035106.1 M15 family metallopeptidase [Exiguobacterium profundum] | 0.9069 | 21 |
| WP_035387862.1 MULTISPECIES: cardiolipin synthase [Exiguobacterium] | 0.9065 | 50 |
| WP_047794841.1 MULTISPECIES: carbohydrate ABC transporter permease [Exiguobacterium] | 0.904 | 26 |
| WP_074036684.1 MULTISPECIES: prepilin-type N-terminal cleavage/methylation domain-containing protein [Exiguobacterium] | 0.9007 | 31 |
| WP_074036678.1 DUF2626 domain-containing protein [Exiguobacterium profundum] | 0.8985 | 25 |
| WP_074036011.1 trypsin-like serine protease [Exiguobacterium profundum] | 0.8981 | 28 |
| WP_074037898.1 S8 family serine peptidase [Exiguobacterium profundum] | 0.8966 | 34 |
| WP_074034769.1 DMT family transporter [Exiguobacterium profundum] | 0.8897 | 58 |
| WP_074034931.1 MULTISPECIES: OmpA family protein [Exiguobacterium] | 0.8854 | 39 |
| WP_074037655.1 ABC transporter ATP-binding protein [Exiguobacterium profundum] | 0.8837 | 28 |
| WP_074037755.1 MFS transporter [Exiguobacterium profundum] | 0.8804 | 20 |
| WP_012726359.1 MULTISPECIES: DUF4397 domain-containing protein [Exiguobacterium] | 0.8777 | 23 |
| WP_074037689.1 penicillin-binding protein [Exiguobacterium profundum] | 0.8624 | 23 |
| WP_074036537.1 hypothetical protein [Exiguobacterium profundum] | 0.8619 | 53 |
| WP_074037854.1 MULTISPECIES: MFS transporter [Exiguobacterium] | 0.8608 | 38 |
| WP_074037891.1 YitT family protein [Exiguobacterium profundum] | 0.8601 | 44 |
| WP_074037238.1 MULTISPECIES: flagellar biosynthesis protein FliO [Exiguobacterium] | 0.8593 | 22 |
| WP_031422513.1 MULTISPECIES: ABC transporter substrate-binding protein [Exiguobacterium] | 0.859 | 27 |
| WP_074037557.1 ABC transporter permease subunit [Exiguobacterium profundum] | 0.8564 | 35 |
| WP_074035755.1 hypothetical protein [Exiguobacterium profundum] | 0.8561 | 18 |
| WP_084543015.1 HlyC/CorC family transporter [Exiguobacterium profundum] | 0.8518 | 16 |
| WP_074037484.1 MULTISPECIES: DedA family protein [Exiguobacterium] | 0.8471 | 59 |
| WP_012726454.1 MULTISPECIES: YneF family protein [Exiguobacterium] | 0.8441 | 19 |
| WP_074035061.1 molybdate ABC transporter substrate-binding protein [Exiguobacterium profundum] | 0.8423 | 22 |
| WP_074035170.1 ABC transporter substrate-binding protein [Exiguobacterium profundum] | 0.8422 | 16 |
| WP_074034733.1 PTS sugar transporter subunit IIC [Exiguobacterium profundum] | 0.8387 | 56 |
| WP_074036973.1 YihY/virulence factor BrkB family protein [Exiguobacterium profundum] | 0.8378 | 51 |
| WP_074035827.1 polysaccharide deacetylase family protein [Exiguobacterium profundum] | 0.8338 | 21 |
| WP_047795511.1 MULTISPECIES: hypothetical protein [Exiguobacterium] | 0.8311 | 22 |
| WP_074035849.1 DUF2512 family protein [Exiguobacterium profundum] | 0.8309 | 29 |
| WP_074036409.1 SH3 domain-containing protein [Exiguobacterium profundum] | 0.8296 | 26 |
| WP_074037707.1 ABC transporter ATP-binding protein [Exiguobacterium profundum] | 0.8265 | 46 |
| WP_074036750.1 DUF4430 domain-containing protein [Exiguobacterium profundum] | 0.8258 | 21 |
| WP_047795551.1 MULTISPECIES: CvpA family protein [Exiguobacterium] | 0.8226 | 17 |
| WP_074037099.1 Bax inhibitor-1/YccA family protein [Exiguobacterium profundum] | 0.8213 | 47 |
| WP_074036523.1 HAMP domain-containing sensor histidine kinase [Exiguobacterium profundum] | 0.8203 | 28 |
| WP_074037026.1 hypothetical protein [Exiguobacterium profundum] | 0.8198 | 29 |
| WP_047795254.1 MULTISPECIES: alpha/beta fold hydrolase [Exiguobacterium] | 0.8195 | 28 |
| WP_074035026.1 MULTISPECIES: copper amine oxidase [Exiguobacterium] | 0.8193 | 26 |
| WP_015880821.1 MULTISPECIES: cytochrome b5 [Exiguobacterium] | 0.8187 | 31 |
| WP_167365519.1 hypothetical protein [Exiguobacterium profundum] | 0.8187 | 22 |
| WP_167365514.1 iron chelate uptake ABC transporter family permease subunit [Exiguobacterium profundum] | 0.8184 | 29 |
| WP_074037389.1 hypothetical protein [Exiguobacterium profundum] | 0.815 | 20 |
| WP_074037511.1 hypothetical protein [Exiguobacterium profundum] | 0.8127 | 26 |
| WP_074036900.1 sulfite exporter TauE/SafE family protein [Exiguobacterium profundum] | 0.812 | 59 |
| WP_031422281.1 MULTISPECIES: DNA-directed RNA polymerase subunit beta [Exiguobacterium] | 0.8098 | 58 |
| WP_084543099.1 MULTISPECIES: endolytic transglycosylase MltG [Exiguobacterium] | 0.8094 | 37 |
| WP_074037758.1 hypothetical protein [Exiguobacterium profundum] | 0.806 | 25 |
| WP_074036498.1 SH3 domain-containing protein [Exiguobacterium profundum] | 0.8044 | 21 |
| WP_143180243.1 ribonuclease Y [Exiguobacterium profundum] | 0.8037 | 20 |
| WP_074037683.1 amino acid ABC transporter permease [Exiguobacterium profundum] | 0.8013 | 41 |
| WP_074034738.1 ABC transporter substrate-binding protein [Exiguobacterium profundum] | 0.8011 | 19 |
| WP_074036187.1 LCP family protein [Exiguobacterium profundum] | 0.7994 | 29 |
| WP_074035035.1 LrgB family protein [Exiguobacterium profundum] | 0.7993 | 48 |
| WP_074037428.1 MULTISPECIES: peptide ABC transporter substrate-binding protein [Exiguobacterium] | 0.798 | 21 |
| WP_031422912.1 MULTISPECIES: carbon starvation protein A [Exiguobacterium] | 0.7951 | 19 |
| WP_074037987.1 hypothetical protein [Exiguobacterium profundum] | 0.7936 | 38 |
| WP_074037888.1 SulP family inorganic anion transporter [Exiguobacterium profundum] | 0.7928 | 32 |
| WP_012726394.1 MULTISPECIES: hypothetical protein [Exiguobacterium] | 0.7927 | 22 |
| WP_012727536.1 MULTISPECIES: hypothetical protein [Exiguobacterium] | 0.7923 | 20 |
| WP_074037075.1 PTS sugar transporter subunit IIC [Exiguobacterium profundum] | 0.7922 | 23 |
| WP_015880358.1 MULTISPECIES: hypothetical protein [Exiguobacterium] | 0.7888 | 23 |
| WP_074037607.1 cation transporter [Exiguobacterium profundum] | 0.7884 | 37 |
| WP_012727200.1 hypothetical protein [Exiguobacterium profundum] | 0.7864 | 44 |
| WP_074037785.1 multidrug efflux SMR transporter [Exiguobacterium profundum] | 0.7862 | 22 |
| WP_074037963.1 efflux RND transporter permease subunit [Exiguobacterium profundum] | 0.7861 | 27 |
| WP_074037235.1 flagellar biosynthesis protein FlhB [Exiguobacterium profundum] | 0.7852 | 64 |
| WP_074036477.1 MULTISPECIES: DUF948 domain-containing protein [Exiguobacterium] | 0.7849 | 29 |
| WP_074037600.1 VanZ family protein [Exiguobacterium profundum] | 0.7849 | 23 |
| WP_031421006.1 MULTISPECIES: hypothetical protein [Exiguobacterium] | 0.7848 | 24 |
| WP_074034809.1 TM2 domain-containing protein [Exiguobacterium profundum] | 0.7848 | 41 |
| WP_074034958.1 MFS transporter [Exiguobacterium profundum] | 0.7819 | 39 |
| WP_074035976.1 multidrug efflux SMR transporter [Exiguobacterium profundum] | 0.7782 | 60 |
| WP_074037380.1 DUF3153 domain-containing protein [Exiguobacterium profundum] | 0.7774 | 25 |
| WP_074035627.1 sodium-dependent bicarbonate transport family permease [Exiguobacterium profundum] | 0.7768 | 22 |
| WP_143180199.1 Na+/H+ antiporter NhaC family protein [Exiguobacterium profundum] | 0.7766 | 27 |
| WP_074035585.1 hypothetical protein [Exiguobacterium profundum] | 0.7749 | 62 |
| WP_074035719.1 hypothetical protein [Exiguobacterium profundum] | 0.773 | 19 |
| WP_074036180.1 acyltransferase family protein [Exiguobacterium profundum] | 0.7709 | 28 |
| WP_074035672.1 VTT domain-containing protein [Exiguobacterium profundum] | 0.7684 | 52 |
| WP_074036149.1 hypothetical protein [Exiguobacterium profundum] | 0.7679 | 20 |
| WP_012726129.1 MULTISPECIES: hypothetical protein [Exiguobacterium] | 0.7673 | 40 |
| WP_012726902.1 MULTISPECIES: preprotein translocase subunit SecG [Exiguobacterium] | 0.764 | 29 |
| WP_074036055.1 ABC transporter permease [Exiguobacterium profundum] | 0.7633 | 40 |
| WP_051829405.1 MULTISPECIES: hemolysin III family protein [Exiguobacterium] | 0.7629 | 46 |
| WP_074037703.1 ABC transporter substrate-binding protein [Exiguobacterium profundum] | 0.7616 | 27 |
| WP_074037640.1 MULTISPECIES: extracellular solute-binding protein [Exiguobacterium] | 0.7613 | 21 |
| WP_074037150.1 sodium-dependent transporter [Exiguobacterium profundum] | 0.757 | 59 |
| WP_074035207.1 MULTISPECIES: HNH endonuclease [Exiguobacterium] | 0.7566 | 20 |
| WP_074035427.1 hypothetical protein [Exiguobacterium profundum] | 0.7555 | 29 |
| WP_074035784.1 hypothetical protein [Exiguobacterium profundum] | 0.7537 | 21 |
| WP_074037796.1 peptide ABC transporter substrate-binding protein [Exiguobacterium profundum] | 0.7537 | 22 |
| WP_074036438.1 MFS transporter [Exiguobacterium profundum] | 0.7509 | 29 |
| WP_074037708.1 MULTISPECIES: L-lactate permease [Exiguobacterium] | 0.75 | 30 |
| WP_074037718.1 MFS transporter [Exiguobacterium profundum] | 0.7497 | 21 |
| WP_074036727.1 MULTISPECIES: hypothetical protein [Exiguobacterium] | 0.749 | 64 |
| WP_031423281.1 MULTISPECIES: phosphate ABC transporter permease subunit PstC [Exiguobacterium] | 0.7468 | 47 |
| WP_015881221.1 MULTISPECIES: cardiolipin synthase [Exiguobacterium] | 0.7453 | 22 |
| WP_074037092.1 TRAP transporter large permease subunit [Exiguobacterium profundum] | 0.745 | 36 |
| WP_015881285.1 cytochrome c oxidase subunit IV [Exiguobacterium profundum] | 0.7437 | 43 |
| WP_074035566.1 sugar ABC transporter substrate-binding protein [Exiguobacterium profundum] | 0.7436 | 22 |
| WP_074034812.1 MULTISPECIES: iron ABC transporter permease [Exiguobacterium] | 0.7429 | 39 |
| WP_074037010.1 sodium:proton antiporter [Exiguobacterium profundum] | 0.7423 | 24 |
| WP_074037432.1 peptide ABC transporter substrate-binding protein [Exiguobacterium profundum] | 0.7398 | 27 |
| WP_074036262.1 M23 family metallopeptidase [Exiguobacterium profundum] | 0.7395 | 20 |
| WP_074036686.1 prepilin-type cleavage/methylation domain-containing protein [Exiguobacterium profundum] | 0.7391 | 24 |
| WP_074036026.1 alginate lyase family protein [Exiguobacterium profundum] | 0.739 | 19 |
| WP_074035761.1 PTS transporter subunit EIIC [Exiguobacterium profundum] | 0.7388 | 36 |
| WP_074035058.1 DUF3221 domain-containing protein [Exiguobacterium profundum] | 0.7367 | 23 |
| WP_084543073.1 lytic transglycosylase domain-containing protein [Exiguobacterium profundum] | 0.7365 | 26 |
| WP_074036007.1 hypothetical protein [Exiguobacterium profundum] | 0.7355 | 28 |
| WP_074035530.1 MULTISPECIES: DUF423 domain-containing protein [Exiguobacterium] | 0.7354 | 19 |
| WP_074037020.1 hypothetical protein [Exiguobacterium profundum] | 0.7348 | 22 |
| WP_074036914.1 cation:proton antiporter [Exiguobacterium profundum] | 0.7347 | 21 |
| WP_074035389.1 LTA synthase family protein [Exiguobacterium profundum] | 0.7346 | 49 |
| WP_074035907.1 sugar transferase [Exiguobacterium profundum] | 0.7341 | 32 |
| WP_074035044.1 LysM peptidoglycan-binding domain-containing protein [Exiguobacterium profundum] | 0.734 | 25 |
| WP_074035743.1 histidine kinase [Exiguobacterium profundum] | 0.7338 | 21 |
| WP_074038002.1 hypothetical protein [Exiguobacterium profundum] | 0.7332 | 24 |
| WP_074037736.1 hypothetical protein [Exiguobacterium profundum] | 0.7329 | 45 |
| WP_012727334.1 MULTISPECIES: hypothetical protein [Exiguobacterium] | 0.7327 | 22 |
| WP_074036446.1 hypothetical protein [Exiguobacterium profundum] | 0.7327 | 19 |
| WP_074035715.1 HAMP domain-containing histidine kinase [Exiguobacterium profundum] | 0.7325 | 29 |
| WP_074034881.1 DUF456 family protein [Exiguobacterium profundum] | 0.7322 | 18 |
| WP_074037884.1 C40 family peptidase [Exiguobacterium profundum] | 0.7322 | 21 |
| WP_074036376.1 MULTISPECIES: DUF975 family protein [Exiguobacterium] | 0.7275 | 32 |
| WP_074036896.1 ABC transporter ATP-binding protein [Exiguobacterium profundum] | 0.7272 | 37 |
| WP_084543070.1 arabinan endo-1,5-alpha-L-arabinosidase [Exiguobacterium profundum] | 0.726 | 22 |
| WP_084543076.1 MetQ/NlpA family ABC transporter substrate-binding protein [Exiguobacterium profundum] | 0.7232 | 28 |
| WP_074036758.1 hypothetical protein [Exiguobacterium profundum] | 0.7227 | 29 |
| WP_074034934.1 MULTISPECIES: HAMP domain-containing histidine kinase [Exiguobacterium] | 0.7216 | 25 |
| WP_074037747.1 MULTISPECIES: b(o/a)3-type cytochrome-c oxidase subunit 1 [Exiguobacterium] | 0.7213 | 47 |
| WP_074037876.1 iron-siderophore ABC transporter substrate-binding protein [Exiguobacterium profundum] | 0.7205 | 19 |
| WP_074036366.1 branched-chain amino acid transport system II carrier protein [Exiguobacterium profundum] | 0.72 | 64 |
| WP_012727017.1 MULTISPECIES: hypothetical protein [Exiguobacterium] | 0.7198 | 30 |
| WP_074036986.1 AI-2E family transporter [Exiguobacterium profundum] | 0.7198 | 26 |
| WP_074037626.1 hypothetical protein [Exiguobacterium profundum] | 0.719 | 23 |
| WP_074034966.1 TVP38/TMEM64 family protein [Exiguobacterium profundum] | 0.7178 | 23 |
| WP_015881397.1 MULTISPECIES: flagellar basal body protein FliL [Exiguobacterium] | 0.7169 | 29 |
| WP_074037009.1 cardiolipin synthase [Exiguobacterium profundum] | 0.7169 | 24 |
| WP_074036933.1 sugar ABC transporter substrate-binding protein [Exiguobacterium profundum] | 0.7161 | 28 |
| WP_074034736.1 hypothetical protein [Exiguobacterium profundum] | 0.7138 | 23 |
| WP_074036585.1 S8 family serine peptidase [Exiguobacterium profundum] | 0.7136 | 25 |
| WP_074036138.1 HAMP domain-containing histidine kinase [Exiguobacterium profundum] | 0.7131 | 50 |
| WP_074037164.1 peptidoglycan glycosyltransferase [Exiguobacterium profundum] | 0.7128 | 52 |
| WP_074035683.1 YxeA family protein [Exiguobacterium profundum] | 0.7119 | 20 |
| WP_074034923.1 MULTISPECIES: restriction endonuclease [Exiguobacterium] | 0.7116 | 41 |
| WP_074035617.1 MULTISPECIES: dicarboxylate/amino acid:cation symporter [Exiguobacterium] | 0.7076 | 22 |
| WP_074036712.1 DUF2627 domain-containing protein [Exiguobacterium profundum] | 0.7076 | 16 |
| WP_074035929.1 EAL domain-containing protein [Exiguobacterium profundum] | 0.7072 | 34 |
| WP_074034750.1 MULTISPECIES: hypothetical protein [Exiguobacterium] | 0.7066 | 54 |
| WP_074036432.1 hypothetical protein [Exiguobacterium profundum] | 0.7048 | 53 |
| WP_074035987.1 iron chelate uptake ABC transporter family permease subunit [Exiguobacterium profundum] | 0.7046 | 52 |
| WP_074036623.1 MULTISPECIES: sensor histidine kinase [Exiguobacterium] | 0.7033 | 34 |
| WP_074037719.1 MULTISPECIES: DMT family transporter [Exiguobacterium] | 0.7033 | 23 |
| WP_074036562.1 BMP family ABC transporter substrate-binding protein [Exiguobacterium profundum] | 0.7029 | 22 |
| WP_074037209.1 M15 family metallopeptidase [Exiguobacterium profundum] | 0.7004 | 21 |
| WP_074036162.1 MFS transporter [Exiguobacterium profundum] | 0.6991 | 34 |
| WP_084543133.1 peptidoglycan DD-metalloendopeptidase family protein [Exiguobacterium profundum] | 0.6988 | 44 |
| WP_074037661.1 HAMP domain-containing histidine kinase [Exiguobacterium profundum] | 0.6986 | 26 |
| WP_074034976.1 alpha/beta hydrolase [Exiguobacterium profundum] | 0.698 | 22 |
| WP_074035724.1 MULTISPECIES: F0F1 ATP synthase subunit A [Exiguobacterium] | 0.6976 | 41 |
| WP_015880639.1 MULTISPECIES: hypothetical protein [Exiguobacterium] | 0.6967 | 59 |
| WP_074037007.1 MULTISPECIES: amino acid ABC transporter permease [Exiguobacterium] | 0.6956 | 54 |
| WP_074037615.1 diguanylate cyclase [Exiguobacterium profundum] | 0.6935 | 29 |
| WP_074036920.1 MULTISPECIES: sodium:calcium antiporter [Exiguobacterium] | 0.6929 | 16 |
| WP_084543025.1 ribose ABC transporter permease [Exiguobacterium profundum] | 0.6929 | 36 |
| WP_074036784.1 ABC transporter permease subunit [Exiguobacterium profundum] | 0.6906 | 29 |
| WP_074037776.1 MULTISPECIES: isoprenylcysteine carboxylmethyltransferase family protein [Exiguobacterium] | 0.6889 | 21 |
| WP_047795258.1 MULTISPECIES: hypothetical protein [Exiguobacterium] | 0.6887 | 57 |
| WP_074035209.1 DUF3600 domain-containing protein [Exiguobacterium profundum] | 0.6874 | 59 |
| WP_074037556.1 DUF4825 domain-containing protein [Exiguobacterium profundum] | 0.6873 | 20 |
| WP_074035213.1 MBOAT family protein [Exiguobacterium profundum] | 0.6872 | 43 |
| WP_012727360.1 MULTISPECIES: F0F1 ATP synthase subunit C [Exiguobacterium] | 0.6871 | 19 |
| WP_074033432.1 MULTISPECIES: hypothetical protein [Exiguobacterium] | 0.6868 | 58 |
| WP_074036656.1 PstS family phosphate ABC transporter substrate-binding protein [Exiguobacterium profundum] | 0.6854 | 23 |
| WP_015880941.1 MULTISPECIES: ammonium transporter [Exiguobacterium] | 0.685 | 28 |
| WP_074035230.1 hypothetical protein [Exiguobacterium profundum] | 0.685 | 31 |
| WP_074035879.1 extracellular solute-binding protein [Exiguobacterium profundum] | 0.6847 | 25 |
| WP_074036081.1 type I pullulanase [Exiguobacterium profundum] | 0.684 | 29 |
| WP_074035094.1 zinc metalloprotease HtpX [Exiguobacterium profundum] | 0.6821 | 33 |
| WP_074037502.1 hypothetical protein [Exiguobacterium profundum] | 0.6817 | 24 |
| WP_074035774.1 MULTISPECIES: ABC transporter substrate-binding protein [Exiguobacterium] | 0.6807 | 25 |
| WP_074035931.1 SH3 domain-containing protein [Exiguobacterium profundum] | 0.6803 | 28 |
| WP_074037058.1 hypothetical protein [Exiguobacterium profundum] | 0.6799 | 42 |
| WP_074034817.1 hypothetical protein [Exiguobacterium profundum] | 0.6784 | 21 |
| WP_084543063.1 DUF92 domain-containing protein [Exiguobacterium profundum] | 0.6784 | 58 |
| WP_074037598.1 M23 family metallopeptidase [Exiguobacterium profundum] | 0.6781 | 26 |
| WP_074035011.1 MFS transporter [Exiguobacterium profundum] | 0.678 | 39 |
| WP_074037753.1 M48 family metalloprotease [Exiguobacterium profundum] | 0.6766 | 29 |
| WP_074034888.1 zinc ABC transporter solute-binding protein [Exiguobacterium profundum] | 0.6757 | 20 |
| WP_074034752.1 HAMP domain-containing histidine kinase [Exiguobacterium profundum] | 0.6748 | 27 |
| WP_074037115.1 peptidase M4 family protein [Exiguobacterium profundum] | 0.6739 | 22 |
| WP_074036964.1 HAMP domain-containing histidine kinase [Exiguobacterium profundum] | 0.6721 | 23 |
| WP_084543121.1 MULTISPECIES: TVP38/TMEM64 family protein [Exiguobacterium] | 0.6712 | 27 |
| WP_074034975.1 MFS transporter [Exiguobacterium profundum] | 0.6704 | 25 |
| WP_074037155.1 hypothetical protein [Exiguobacterium profundum] | 0.6697 | 26 |
| WP_074037362.1 FtsW/RodA/SpoVE family cell cycle protein [Exiguobacterium profundum] | 0.6697 | 29 |
| WP_074037133.1 transporter substrate-binding domain-containing protein [Exiguobacterium profundum] | 0.6693 | 16 |
| WP_074034886.1 alpha/beta hydrolase [Exiguobacterium profundum] | 0.6685 | 22 |
| WP_074034897.1 YwiC-like family protein [Exiguobacterium profundum] | 0.6684 | 26 |
| WP_143180265.1 MULTISPECIES: hypothetical protein [Exiguobacterium] | 0.6675 | 16 |
| WP_074035688.1 hypothetical protein [Exiguobacterium profundum] | 0.6671 | 59 |
| WP_074036220.1 HAMP domain-containing protein [Exiguobacterium profundum] | 0.6665 | 60 |
| WP_074036401.1 PTS transporter subunit EIIC [Exiguobacterium profundum] | 0.6664 | 35 |
| WP_074035379.1 DUF2975 domain-containing protein [Exiguobacterium profundum] | 0.6661 | 22 |
| WP_074035833.1 bifunctional diguanylate cyclase/phosphodiesterase [Exiguobacterium profundum] | 0.6655 | 39 |
| WP_074036694.1 rhodanese-like domain-containing protein [Exiguobacterium profundum] | 0.6651 | 33 |
| WP_012727725.1 MULTISPECIES: septum formation initiator [Exiguobacterium] | 0.6647 | 53 |
| WP_074034890.1 hypothetical protein [Exiguobacterium profundum] | 0.6645 | 45 |
| WP_074036607.1 MULTISPECIES: diacylglycerol kinase family protein [Exiguobacterium] | 0.6644 | 38 |
| WP_074037465.1 MULTISPECIES: zinc transporter ZupT [Exiguobacterium] | 0.6632 | 23 |
| WP_084543102.1 S8 family serine peptidase [Exiguobacterium profundum] | 0.6629 | 21 |
| WP_074034771.1 hypothetical protein [Exiguobacterium profundum] | 0.6628 | 37 |
| WP_074036354.1 ABC transporter substrate-binding protein [Exiguobacterium profundum] | 0.6611 | 22 |
| WP_074036113.1 methyl-accepting chemotaxis protein [Exiguobacterium profundum] | 0.6608 | 23 |
| WP_074034709.1 EAL domain-containing protein [Exiguobacterium profundum] | 0.6607 | 30 |
| WP_074036908.1 CapA family protein [Exiguobacterium profundum] | 0.6602 | 27 |
| WP_074037551.1 Na+/H+ antiporter subunit A [Exiguobacterium profundum] | 0.6592 | 54 |
| WP_012726605.1 MULTISPECIES: L-lactate dehydrogenase [Exiguobacterium] | 0.6588 | 25 |
| WP_074036822.1 cytochrome C biogenesis protein ResB [Exiguobacterium profundum] | 0.6586 | 48 |
| WP_074034703.1 SH3 domain-containing protein [Exiguobacterium profundum] | 0.6585 | 23 |
| WP_074036655.1 penicillin-binding protein 2 [Exiguobacterium profundum] | 0.6582 | 37 |
| WP_074037760.1 carbohydrate ABC transporter substrate-binding protein [Exiguobacterium profundum] | 0.6572 | 29 |
| WP_074037045.1 MFS transporter [Exiguobacterium profundum] | 0.6571 | 61 |
| WP_074037407.1 rod shape-determining protein RodA [Exiguobacterium profundum] | 0.657 | 28 |
| WP_047795099.1 MULTISPECIES: hypothetical protein [Exiguobacterium] | 0.6566 | 19 |
| WP_074036992.1 hypothetical protein [Exiguobacterium profundum] | 0.6558 | 20 |
| WP_074037824.1 LPXTG cell wall anchor domain-containing protein [Exiguobacterium profundum] | 0.6542 | 29 |
| WP_084543060.1 MULTISPECIES: DUF1002 domain-containing protein [Exiguobacterium] | 0.654 | 22 |
| WP_074036009.1 hypothetical protein [Exiguobacterium profundum] | 0.6525 | 24 |
| WP_074035603.1 glycoside hydrolase family 3 [Exiguobacterium profundum] | 0.6524 | 24 |
| WP_143180238.1 hypothetical protein [Exiguobacterium profundum] | 0.6516 | 22 |
| WP_074035190.1 family 16 glycosylhydrolase [Exiguobacterium profundum] | 0.6512 | 18 |
| WP_074037722.1 1,4-dihydroxy-2-naphthoate polyprenyltransferase [Exiguobacterium profundum] | 0.6512 | 53 |
| WP_074037237.1 MULTISPECIES: flagellar type III secretion system pore protein FliP [Exiguobacterium] | 0.6504 | 34 |
| WP_074037513.1 hypothetical protein [Exiguobacterium profundum] | 0.6491 | 29 |
| WP_074037945.1 cytochrome c oxidase subunit I [Exiguobacterium profundum] | 0.649 | 41 |
| WP_031421923.1 MULTISPECIES: hypothetical protein [Exiguobacterium] | 0.6485 | 19 |
| WP_074036766.1 DMT family transporter [Exiguobacterium profundum] | 0.6483 | 19 |
| WP_074035513.1 thiol reductant ABC exporter subunit CydD [Exiguobacterium profundum] | 0.648 | 36 |
| WP_047795500.1 MULTISPECIES: rod shape-determining protein MreC [Exiguobacterium] | 0.6469 | 25 |
| WP_074036030.1 alginate lyase family protein [Exiguobacterium profundum] | 0.6469 | 27 |
| WP_015881403.1 MULTISPECIES: flagellar biosynthesis protein FliQ [Exiguobacterium] | 0.6441 | 40 |
| WP_074036067.1 transcriptional regulator [Exiguobacterium profundum] | 0.6434 | 28 |
| WP_015881158.1 MULTISPECIES: HlyC/CorC family transporter [Exiguobacterium] | 0.6428 | 27 |
| WP_074035662.1 (Fe-S)-binding protein [Exiguobacterium profundum] | 0.6427 | 19 |
| WP_074034910.1 GGDEF domain-containing protein [Exiguobacterium profundum] | 0.6416 | 27 |
| WP_074036963.1 ABC transporter permease [Exiguobacterium profundum] | 0.6408 | 29 |
| WP_074037587.1 FtsX-like permease family protein [Exiguobacterium profundum] | 0.6396 | 39 |
| WP_074037691.1 MULTISPECIES: DUF5366 family protein [Exiguobacterium] | 0.6385 | 28 |
| WP_074036689.1 MULTISPECIES: hypothetical protein [Exiguobacterium] | 0.6376 | 25 |
| WP_074035029.1 Fe(3+) ABC transporter substrate-binding protein [Exiguobacterium profundum] | 0.6361 | 25 |
| WP_143180241.1 hypothetical protein [Exiguobacterium profundum] | 0.6356 | 16 |
| WP_047795803.1 MULTISPECIES: MFS transporter [Exiguobacterium] | 0.6353 | 55 |
| WP_074037621.1 hypothetical protein [Exiguobacterium profundum] | 0.6347 | 17 |
| WP_074036873.1 cytochrome C biogenesis protein [Exiguobacterium profundum] | 0.6341 | 19 |
| WP_012726630.1 MULTISPECIES: DUF1427 family protein [Exiguobacterium] | 0.633 | 20 |
| WP_074034744.1 glucosaminidase domain-containing protein [Exiguobacterium profundum] | 0.6325 | 23 |
| WP_031424443.1 MULTISPECIES: membrane protein [Exiguobacterium] | 0.6323 | 50 |
| WP_074034766.1 hypothetical protein [Exiguobacterium profundum] | 0.632 | 40 |
| WP_031422851.1 MULTISPECIES: cytochrome c [Exiguobacterium] | 0.6316 | 20 |
| WP_074035570.1 ABC transporter permease [Exiguobacterium profundum] | 0.6315 | 37 |
| WP_074036487.1 penicillin-binding protein [Exiguobacterium profundum] | 0.6308 | 46 |
| WP_074037060.1 MULTISPECIES: DUF817 domain-containing protein [Exiguobacterium] | 0.6289 | 58 |
| WP_074037930.1 PDZ domain-containing protein [Exiguobacterium profundum] | 0.6289 | 19 |
| WP_074035399.1 hypothetical protein [Exiguobacterium profundum] | 0.6288 | 22 |
| WP_074034913.1 VTT domain-containing protein [Exiguobacterium profundum] | 0.6283 | 49 |
| WP_074036871.1 ABC transporter ATP-binding protein [Exiguobacterium profundum] | 0.6281 | 40 |
| WP_074034977.1 purine/pyrimidine permease [Exiguobacterium profundum] | 0.6272 | 60 |
| WP_074036211.1 multidrug resistance efflux transporter family protein [Exiguobacterium profundum] | 0.627 | 51 |
| WP_031424520.1 MULTISPECIES: hypothetical protein [Exiguobacterium] | 0.6267 | 21 |
| WP_012726441.1 MULTISPECIES: hypothetical protein [Exiguobacterium] | 0.6265 | 25 |
| WP_074035141.1 hypothetical protein [Exiguobacterium profundum] | 0.6252 | 28 |
| WP_074035693.1 ABC transporter permease [Exiguobacterium profundum] | 0.6252 | 42 |
| WP_074034767.1 DMT family transporter [Exiguobacterium profundum] | 0.6251 | 50 |
| WP_074036264.1 PDZ domain-containing protein [Exiguobacterium profundum] | 0.6244 | 20 |
| WP_074036602.1 hypothetical protein [Exiguobacterium profundum] | 0.6243 | 19 |
| WP_074037548.1 MULTISPECIES: Na+/H+ antiporter subunit D [Exiguobacterium] | 0.6242 | 53 |
| WP_074035792.1 TerC family protein [Exiguobacterium profundum] | 0.624 | 16 |
| WP_074037051.1 MULTISPECIES: GGDEF domain-containing protein [Exiguobacterium] | 0.6237 | 26 |
| WP_074036927.1 glucose transporter GlcU [Exiguobacterium profundum] | 0.6233 | 18 |
| WP_074037947.1 multidrug efflux MFS transporter [Exiguobacterium profundum] | 0.6231 | 23 |
| WP_015880386.1 MULTISPECIES: MFS transporter [Exiguobacterium] | 0.623 | 30 |
| WP_084543107.1 GAF domain-containing protein [Exiguobacterium profundum] | 0.6226 | 31 |
| WP_012727458.1 MULTISPECIES: hypothetical protein [Exiguobacterium] | 0.6225 | 25 |
| WP_074035841.1 DUF2273 domain-containing protein [Exiguobacterium profundum] | 0.6224 | 33 |
| WP_074037956.1 DUF5057 domain-containing protein [Exiguobacterium profundum] | 0.6224 | 20 |
| WP_074036323.1 STAS domain-containing protein [Exiguobacterium profundum] | 0.6222 | 44 |
| WP_074037006.1 transporter substrate-binding domain-containing protein [Exiguobacterium profundum] | 0.6216 | 21 |
| WP_074034805.1 MFS transporter [Exiguobacterium profundum] | 0.6214 | 59 |
| WP_074037079.1 methyl-accepting chemotaxis protein [Exiguobacterium profundum] | 0.6211 | 28 |
| WP_074035753.1 large conductance mechanosensitive channel protein MscL [Exiguobacterium profundum] | 0.621 | 33 |
| WP_074034700.1 MULTISPECIES: hypothetical protein [Exiguobacterium] | 0.6209 | 23 |
| WP_015880836.1 MULTISPECIES: cell wall-active antibiotics response protein [Exiguobacterium] | 0.6203 | 28 |
| WP_074036737.1 amino acid ABC transporter permease [Exiguobacterium profundum] | 0.6198 | 35 |
| WP_074037604.1 metallophosphoesterase [Exiguobacterium profundum] | 0.619 | 23 |
| WP_143180200.1 MULTISPECIES: divergent PAP2 family protein [Exiguobacterium] | 0.6189 | 24 |
| WP_074034716.1 hypothetical protein [Exiguobacterium profundum] | 0.6183 | 16 |
| WP_143180248.1 MULTISPECIES: hypothetical protein [Exiguobacterium] | 0.6177 | 33 |
| WP_015880847.1 MULTISPECIES: hypothetical protein [Exiguobacterium] | 0.6173 | 49 |
| WP_074036993.1 hypothetical protein [Exiguobacterium profundum] | 0.6162 | 16 |
| WP_074036800.1 MFS transporter [Exiguobacterium profundum] | 0.6158 | 17 |
| WP_015880893.1 MULTISPECIES: metal-dependent hydrolase [Exiguobacterium] | 0.6152 | 43 |
| WP_074036260.1 ABC transporter permease [Exiguobacterium profundum] | 0.6151 | 42 |
| WP_031421243.1 MULTISPECIES: YitT family protein [Exiguobacterium] | 0.6148 | 37 |
| WP_074037889.1 hypothetical protein [Exiguobacterium profundum] | 0.6148 | 18 |
| WP_047795079.1 MULTISPECIES: aquaporin family protein [Exiguobacterium] | 0.6147 | 23 |
| WP_074035583.1 MATE family efflux transporter [Exiguobacterium profundum] | 0.6145 | 33 |
| WP_074037056.1 ABC transporter permease subunit [Exiguobacterium profundum] | 0.6144 | 27 |
| WP_074037919.1 cytochrome c biogenesis protein CcdC [Exiguobacterium profundum] | 0.6138 | 17 |
| WP_074037780.1 two pore domain potassium channel family protein [Exiguobacterium profundum] | 0.613 | 41 |
| WP_074037356.1 MULTISPECIES: protoheme IX farnesyltransferase [Exiguobacterium] | 0.6125 | 50 |
| WP_031421292.1 MULTISPECIES: hemolysin III family protein [Exiguobacterium] | 0.6124 | 59 |
| WP_074035013.1 MULTISPECIES: DoxX family membrane protein [Exiguobacterium] | 0.6121 | 33 |
| WP_074035117.1 mechanosensitive ion channel [Exiguobacterium profundum] | 0.6109 | 32 |
| WP_074037028.1 PH domain-containing protein [Exiguobacterium profundum] | 0.6108 | 53 |
| WP_074036057.1 ABC transporter permease [Exiguobacterium profundum] | 0.6106 | 42 |
| WP_074034930.1 flagellar motor stator protein MotA [Exiguobacterium profundum] | 0.61 | 19 |
| WP_074038001.1 hypothetical protein [Exiguobacterium profundum] | 0.6083 | 35 |
| WP_074035928.1 UDP-glucose/GDP-mannose dehydrogenase family protein [Exiguobacterium profundum] | 0.6079 | 22 |
| WP_074037367.1 hypothetical protein [Exiguobacterium profundum] | 0.6079 | 54 |
| WP_074035378.1 YdhK family protein [Exiguobacterium profundum] | 0.6077 | 22 |
| WP_074036559.1 NfeD family protein [Exiguobacterium profundum] | 0.6075 | 59 |
| WP_074034919.1 phosphatidate cytidylyltransferase [Exiguobacterium profundum] | 0.6074 | 61 |
| WP_074034692.1 DUF58 domain-containing protein [Exiguobacterium profundum] | 0.6065 | 21 |
| WP_074036061.1 CAP domain-containing protein [Exiguobacterium profundum] | 0.6064 | 19 |
| WP_074036052.1 hypothetical protein [Exiguobacterium profundum] | 0.6054 | 55 |
| WP_074036517.1 DUF441 domain-containing protein [Exiguobacterium profundum] | 0.6054 | 17 |
| WP_074037773.1 MULTISPECIES: hypothetical protein [Exiguobacterium] | 0.6047 | 25 |
| WP_074036400.1 MULTISPECIES: PTS transporter subunit EIIC [Exiguobacterium] | 0.6041 | 35 |
| WP_074037454.1 membrane protein [Exiguobacterium profundum] | 0.6037 | 30 |
| WP_074036912.1 DedA family protein [Exiguobacterium profundum] | 0.6034 | 64 |
| WP_015880919.1 MULTISPECIES: transporter substrate-binding domain-containing protein [Exiguobacterium] | 0.6031 | 19 |
| WP_074035515.1 thiol reductant ABC exporter subunit CydC [Exiguobacterium profundum] | 0.6019 | 46 |
| WP_074037243.1 flagellar hook-length control protein FliK [Exiguobacterium profundum] | 0.6019 | 45 |
| WP_031422139.1 MULTISPECIES: metalloprotease [Exiguobacterium] | 0.601 | 48 |
| WP_047795569.1 MULTISPECIES: disulfide bond formation protein B [Exiguobacterium] | 0.6008 | 23 |
| WP_074037874.1 hypothetical protein [Exiguobacterium profundum] | 0.5993 | 23 |
| WP_074035197.1 alkaline phosphatase [Exiguobacterium profundum] | 0.599 | 20 |
| WP_012726920.1 MULTISPECIES: hypothetical protein [Exiguobacterium] | 0.5981 | 48 |
| WP_074036364.1 L-lactate dehydrogenase [Exiguobacterium profundum] | 0.5975 | 31 |
| WP_074035726.1 MULTISPECIES: F0F1 ATP synthase subunit B [Exiguobacterium] | 0.5973 | 40 |
| WP_074034960.1 Na+/H+ antiporter NhaC [Exiguobacterium profundum] | 0.5969 | 28 |
| WP_074037467.1 MULTISPECIES: methyl-accepting chemotaxis protein [Exiguobacterium] | 0.5968 | 53 |
| WP_074035034.1 CidA/LrgA family protein [Exiguobacterium profundum] | 0.5966 | 51 |
| WP_074037301.1 hypothetical protein [Exiguobacterium profundum] | 0.5963 | 51 |
| WP_074035757.1 methyl-accepting chemotaxis protein [Exiguobacterium profundum] | 0.5954 | 31 |
| WP_074037546.1 MULTISPECIES: Na+/H+ antiporter subunit E [Exiguobacterium] | 0.5953 | 25 |
| WP_074035090.1 HAMP domain-containing histidine kinase [Exiguobacterium profundum] | 0.595 | 20 |
| WP_074037168.1 hypothetical protein [Exiguobacterium profundum] | 0.595 | 21 |
| WP_074036214.1 hypothetical protein [Exiguobacterium profundum] | 0.5946 | 22 |
| WP_143180256.1 hypothetical protein [Exiguobacterium profundum] | 0.5933 | 17 |
| WP_074035534.1 amino acid permease [Exiguobacterium profundum] | 0.593 | 62 |
| WP_074035945.1 YxeA family protein [Exiguobacterium profundum] | 0.593 | 19 |
| WP_074037495.1 hypothetical protein [Exiguobacterium profundum] | 0.593 | 40 |
| WP_074036959.1 CPBP family intramembrane metalloprotease [Exiguobacterium profundum] | 0.5929 | 63 |
| WP_074037734.1 MULTISPECIES: sensor histidine kinase [Exiguobacterium] | 0.5923 | 30 |
| WP_015880600.1 MULTISPECIES: YdiK family protein [Exiguobacterium] | 0.5915 | 23 |
| WP_074035047.1 thiamine biosynthesis protein ThiF [Exiguobacterium profundum] | 0.5914 | 43 |
| WP_074035973.1 MULTISPECIES: iron export ABC transporter permease subunit FetB [Exiguobacterium] | 0.5913 | 24 |
| WP_074035666.1 hypothetical protein [Exiguobacterium profundum] | 0.5899 | 20 |
| WP_074035599.1 MULTISPECIES: fatty acid desaturase [Exiguobacterium] | 0.589 | 61 |
| WP_074037057.1 ABC transporter permease subunit [Exiguobacterium profundum] | 0.589 | 30 |
| WP_012726919.1 MULTISPECIES: DUF2179 domain-containing protein [Exiguobacterium] | 0.5889 | 19 |
| WP_074036598.1 nfeD-like family protein [Exiguobacterium profundum] | 0.5888 | 41 |
| WP_084543039.1 HAMP domain-containing histidine kinase [Exiguobacterium profundum] | 0.5888 | 31 |
| WP_074037681.1 SLC13 family permease [Exiguobacterium profundum] | 0.5886 | 37 |
| WP_074037946.1 cytochrome c oxidase subunit II [Exiguobacterium profundum] | 0.5884 | 17 |
| WP_074035072.1 hypothetical protein [Exiguobacterium profundum] | 0.5883 | 47 |
| WP_074035517.1 hypothetical protein [Exiguobacterium profundum] | 0.5877 | 17 |
| WP_074035364.1 MULTISPECIES: energy-coupling factor transporter transmembrane protein EcfT [Exiguobacterium] | 0.5876 | 57 |
| WP_074036005.1 S8 family serine peptidase [Exiguobacterium profundum] | 0.5863 | 28 |
| WP_074035219.1 DUF4230 domain-containing protein [Exiguobacterium profundum] | 0.5853 | 22 |
| WP_074036391.1 MULTISPECIES: NupC/NupG family nucleoside CNT transporter [Exiguobacterium] | 0.5844 | 51 |
| WP_074035113.1 MULTISPECIES: branched-chain amino acid transporter [Exiguobacterium] | 0.5842 | 19 |
| WP_074036956.1 thiamine permease [Exiguobacterium profundum] | 0.5841 | 35 |
| WP_074035694.1 DUF421 domain-containing protein [Exiguobacterium profundum] | 0.5838 | 57 |
| WP_074035870.1 LytR family transcriptional regulator [Exiguobacterium profundum] | 0.5836 | 30 |
| WP_074034751.1 ABC transporter permease [Exiguobacterium profundum] | 0.5834 | 40 |
| WP_074035797.1 potassium-transporting ATPase subunit C [Exiguobacterium profundum] | 0.5824 | 40 |
| WP_074035401.1 hypothetical protein [Exiguobacterium profundum] | 0.5816 | 25 |
| WP_074037510.1 prepilin-type N-terminal cleavage/methylation domain-containing protein [Exiguobacterium profundum] | 0.5813 | 37 |
| WP_074034801.1 hypothetical protein [Exiguobacterium profundum] | 0.5811 | 27 |
| WP_074036076.1 FAD-dependent oxidoreductase [Exiguobacterium profundum] | 0.581 | 16 |
| WP_074037152.1 MULTISPECIES: ABC transporter permease/substrate-binding protein [Exiguobacterium] | 0.5806 | 38 |
| WP_074036333.1 bifunctional 2',3'-cyclic-nucleotide 2'-phosphodiesterase/3'-nucleotidase [Exiguobacterium profundum] | 0.5803 | 20 |
| WP_074035045.1 hypothetical protein [Exiguobacterium profundum] | 0.58 | 22 |
| WP_074034826.1 hypothetical protein [Exiguobacterium profundum] | 0.5799 | 54 |
| WP_074035031.1 efflux RND transporter permease subunit [Exiguobacterium profundum] | 0.5797 | 29 |
| WP_074037165.1 MULTISPECIES: endonuclease III [Exiguobacterium] | 0.5793 | 46 |
| WP_074035796.1 potassium-transporting ATPase subunit KdpB [Exiguobacterium profundum] | 0.5792 | 52 |
| WP_012726320.1 MULTISPECIES: DUF58 domain-containing protein [Exiguobacterium] | 0.5791 | 28 |
| WP_074036884.1 M15 family metallopeptidase [Exiguobacterium profundum] | 0.5791 | 22 |
| WP_074036102.1 MULTISPECIES: undecaprenyl-diphosphate phosphatase [Exiguobacterium] | 0.5783 | 17 |
| WP_074035985.1 iron chelate uptake ABC transporter family permease subunit [Exiguobacterium profundum] | 0.5779 | 46 |
| WP_074036032.1 bifunctional metallophosphatase/5'-nucleotidase [Exiguobacterium profundum] | 0.5776 | 24 |
| WP_074037916.1 MULTISPECIES: HAMP domain-containing protein [Exiguobacterium] | 0.5776 | 30 |
| WP_074037720.1 cadmium-translocating P-type ATPase [Exiguobacterium profundum] | 0.5775 | 45 |
| WP_143180236.1 MULTISPECIES: HDIG domain-containing protein [Exiguobacterium] | 0.5753 | 37 |
| WP_031422116.1 MULTISPECIES: potassium channel family protein [Exiguobacterium] | 0.5749 | 34 |
| WP_031423441.1 MULTISPECIES: c-type cytochrome biogenesis protein CcsB [Exiguobacterium] | 0.5746 | 25 |
| WP_074037041.1 hypothetical protein [Exiguobacterium profundum] | 0.5746 | 32 |
| WP_074037693.1 hypothetical protein [Exiguobacterium profundum] | 0.5738 | 30 |
| WP_074035597.1 alpha-amylase [Exiguobacterium profundum] | 0.5734 | 22 |
| WP_074038005.1 hypothetical protein [Exiguobacterium profundum] | 0.5722 | 40 |
| WP_074037971.1 sugar ABC transporter permease [Exiguobacterium profundum] | 0.572 | 22 |
| WP_074034821.1 twin-arginine translocase subunit TatC [Exiguobacterium profundum] | 0.5717 | 38 |
| WP_031424332.1 MULTISPECIES: iron ABC transporter permease [Exiguobacterium] | 0.5713 | 27 |
| WP_074037038.1 hypothetical protein [Exiguobacterium profundum] | 0.5713 | 20 |
| WP_012726857.1 MULTISPECIES: ABC transporter permease [Exiguobacterium] | 0.5712 | 36 |
| WP_074037912.1 lysozyme [Exiguobacterium profundum] | 0.5708 | 19 |
| WP_074037625.1 TlpA family protein disulfide reductase [Exiguobacterium profundum] | 0.5705 | 28 |
| WP_074037224.1 phosphatidate cytidylyltransferase [Exiguobacterium profundum] | 0.5703 | 64 |
| WP_012390467.1 MULTISPECIES: hypothetical protein [Exiguobacterium] | 0.5695 | 25 |
| WP_074035556.1 TerC family protein [Exiguobacterium profundum] | 0.5694 | 24 |
| WP_074036478.1 YtxH domain-containing protein [Exiguobacterium profundum] | 0.5692 | 32 |
| WP_074037353.1 SCO family protein [Exiguobacterium profundum] | 0.5688 | 22 |
| WP_074036600.1 flotillin-like protein FloA [Exiguobacterium profundum] | 0.568 | 31 |
| WP_074037650.1 phytoene desaturase [Exiguobacterium profundum] | 0.568 | 21 |
| WP_159562944.1 MULTISPECIES: hypothetical protein [Exiguobacterium] | 0.568 | 31 |
| WP_074037613.1 MBL fold metallo-hydrolase [Exiguobacterium profundum] | 0.5675 | 20 |
| WP_074036890.1 DUF975 family protein [Exiguobacterium profundum] | 0.5674 | 33 |
| WP_074034779.1 hypothetical protein [Exiguobacterium profundum] | 0.5671 | 54 |
| WP_012727703.1 MULTISPECIES: PIN/TRAM domain-containing protein [Exiguobacterium] | 0.5669 | 24 |
| WP_074037713.1 MULTISPECIES: hypothetical protein [Exiguobacterium] | 0.5668 | 25 |
| WP_031424263.1 MULTISPECIES: HlyC/CorC family transporter [Exiguobacterium] | 0.5666 | 26 |
| WP_074036298.1 triose-phosphate isomerase [Exiguobacterium profundum] | 0.5661 | 53 |
| WP_074035133.1 hypothetical protein [Exiguobacterium profundum] | 0.5653 | 25 |
| WP_074036931.1 DUF624 domain-containing protein [Exiguobacterium profundum] | 0.5653 | 43 |
| WP_167365517.1 hypothetical protein [Exiguobacterium profundum] | 0.5652 | 36 |
| WP_015880545.1 MULTISPECIES: hypothetical protein [Exiguobacterium] | 0.565 | 20 |
| WP_074037507.1 prepilin-type N-terminal cleavage/methylation domain-containing protein [Exiguobacterium profundum] | 0.5648 | 45 |
| WP_074035384.1 MMPL family transporter [Exiguobacterium profundum] | 0.5644 | 26 |
| WP_074036348.1 PTS sugar transporter subunit IIC [Exiguobacterium profundum] | 0.5642 | 34 |
| WP_074037425.1 ABC transporter permease [Exiguobacterium profundum] | 0.5633 | 64 |
| WP_074037334.1 penicillin-binding protein [Exiguobacterium profundum] | 0.5629 | 38 |
| WP_143180279.1 type IV secretory system conjugative DNA transfer family protein [Exiguobacterium profundum] | 0.5627 | 27 |
| WP_074035778.1 diguanylate cyclase [Exiguobacterium profundum] | 0.5624 | 57 |
| WP_074037358.1 heme A synthase [Exiguobacterium profundum] | 0.561 | 31 |
| WP_074035012.1 DUF3817 domain-containing protein [Exiguobacterium profundum] | 0.5606 | 32 |
| WP_074037398.1 MULTISPECIES: PTS ascorbate transporter subunit IIC [Exiguobacterium] | 0.5596 | 28 |
| WP_074034900.1 MMPL family transporter [Exiguobacterium profundum] | 0.5586 | 30 |
| WP_074035206.1 MULTISPECIES: hypothetical protein [Exiguobacterium] | 0.5586 | 24 |
| WP_143180275.1 MULTISPECIES: AI-2E family transporter [Exiguobacterium] | 0.5584 | 24 |
| WP_074036564.1 competence protein ComEA [Exiguobacterium profundum] | 0.5582 | 22 |
| WP_074037570.1 hypothetical protein [Exiguobacterium profundum] | 0.5577 | 21 |
| WP_074035302.1 YhgE/Pip domain-containing protein [Exiguobacterium profundum] | 0.5575 | 35 |
| WP_074036786.1 ABC transporter permease subunit [Exiguobacterium profundum] | 0.5575 | 63 |
| WP_084543074.1 D-alanyl-D-alanine carboxypeptidase [Exiguobacterium profundum] | 0.5571 | 36 |
| WP_074034725.1 hypothetical protein [Exiguobacterium profundum] | 0.5568 | 37 |
| WP_074037731.1 MULTISPECIES: hypothetical protein [Exiguobacterium] | 0.5563 | 16 |
| WP_074035857.1 hypothetical protein [Exiguobacterium profundum] | 0.5561 | 17 |
| WP_074036112.1 sodium:alanine symporter family protein [Exiguobacterium profundum] | 0.5558 | 35 |
| WP_074034734.1 hypothetical protein [Exiguobacterium profundum] | 0.5555 | 46 |
| WP_012727503.1 MULTISPECIES: trimeric intracellular cation channel family protein [Exiguobacterium] | 0.5552 | 46 |
| WP_074035740.1 hypothetical protein [Exiguobacterium profundum] | 0.5551 | 23 |
| WP_015880407.1 MULTISPECIES: FixH family protein [Exiguobacterium] | 0.5548 | 24 |
| WP_001679885.1 MULTISPECIES: major outer membrane lipoprotein [Bacteria] | 0.5542 | 21 |
| WP_074036003.1 hypothetical protein [Exiguobacterium profundum] | 0.5542 | 22 |
| WP_047795765.1 MULTISPECIES: hypothetical protein [Exiguobacterium] | 0.554 | 32 |
| WP_074037031.1 MULTISPECIES: hypothetical protein [Exiguobacterium] | 0.554 | 23 |
| WP_047795606.1 MULTISPECIES: GlsB/YeaQ/YmgE family stress response membrane protein [Exiguobacterium] | 0.5536 | 22 |
| WP_074035795.1 potassium-transporting ATPase subunit A [Exiguobacterium profundum] | 0.552 | 20 |
| WP_074036563.1 methyl-accepting chemotaxis protein [Exiguobacterium profundum] | 0.5514 | 27 |
| WP_074037820.1 TIGR04053 family radical SAM/SPASM domain-containing protein [Exiguobacterium profundum] | 0.5512 | 32 |
| WP_074036826.1 insulinase family protein [Exiguobacterium profundum] | 0.5511 | 36 |
| WP_074037383.1 MULTISPECIES: cytochrome ubiquinol oxidase subunit I [Exiguobacterium] | 0.551 | 27 |
| WP_015880865.1 MULTISPECIES: hypothetical protein [Exiguobacterium] | 0.5507 | 27 |
| WP_074035380.1 DUF975 family protein [Exiguobacterium profundum] | 0.5507 | 35 |
| WP_026825360.1 MULTISPECIES: hypothetical protein [Exiguobacterium] | 0.5488 | 19 |
| WP_074036407.1 MULTISPECIES: sporulation integral membrane protein YtvI [Exiguobacterium] | 0.5488 | 52 |
| WP_074037829.1 TrkH family potassium uptake protein [Exiguobacterium profundum] | 0.5488 | 39 |
| WP_074037674.1 hypothetical protein [Exiguobacterium profundum] | 0.5485 | 28 |
| WP_012726599.1 MULTISPECIES: DUF2759 domain-containing protein [Exiguobacterium] | 0.5483 | 45 |
| WP_084543114.1 MULTISPECIES: hypothetical protein [Exiguobacterium] | 0.5474 | 27 |
| WP_074037196.1 intramembrane metalloprotease PrsW [Exiguobacterium profundum] | 0.5472 | 16 |
| WP_074037442.1 hypothetical protein [Exiguobacterium profundum] | 0.5472 | 63 |
| WP_047795513.1 MULTISPECIES: prepilin-type N-terminal cleavage/methylation domain-containing protein [Exiguobacterium] | 0.5469 | 34 |
| WP_074034762.1 hypothetical protein [Exiguobacterium profundum] | 0.5462 | 39 |
| WP_074037612.1 MFS transporter [Exiguobacterium profundum] | 0.5457 | 60 |
| WP_074037614.1 MULTISPECIES: DsbA family protein [Exiguobacterium] | 0.5455 | 25 |
| WP_074037870.1 hypothetical protein [Exiguobacterium profundum] | 0.5454 | 29 |
| WP_074036289.1 hypothetical protein [Exiguobacterium profundum] | 0.5451 | 54 |
| WP_074037620.1 ABC transporter permease subunit [Exiguobacterium profundum] | 0.5449 | 41 |
| WP_074037059.1 MULTISPECIES: hypothetical protein [Exiguobacterium] | 0.5442 | 50 |
| WP_074037116.1 MULTISPECIES: G-D-S-L family lipolytic protein [Exiguobacterium] | 0.5439 | 23 |
| WP_074035855.1 MULTISPECIES: hypothetical protein [Exiguobacterium] | 0.5437 | 56 |
| WP_074035967.1 AI-2E family transporter [Exiguobacterium profundum] | 0.543 | 50 |
| WP_074036770.1 MULTISPECIES: YtzI protein [Exiguobacterium] | 0.5429 | 28 |
| WP_074035828.1 hypothetical protein [Exiguobacterium profundum] | 0.5426 | 25 |
| WP_074035518.1 GGDEF domain-containing protein [Exiguobacterium profundum] | 0.5425 | 64 |
| WP_074037234.1 flagellar biosynthesis protein FlhA [Exiguobacterium profundum] | 0.5422 | 54 |
| WP_074036658.1 phosphate ABC transporter permease PstA [Exiguobacterium profundum] | 0.5421 | 53 |
| WP_074036832.1 MULTISPECIES: CDP-diacylglycerol--glycerol-3-phosphate 3-phosphatidyltransferase [Exiguobacterium] | 0.5409 | 29 |
| WP_074035105.1 MULTISPECIES: hypothetical protein [Exiguobacterium] | 0.54 | 25 |
| WP_074036593.1 MULTISPECIES: EamA family transporter RarD [Exiguobacterium] | 0.5397 | 54 |
| WP_012726344.1 MULTISPECIES: ABC transporter permease [Exiguobacterium] | 0.5391 | 34 |
| WP_012727687.1 MULTISPECIES: 50S ribosomal protein L7/L12 [Exiguobacterium] | 0.5389 | 50 |
| WP_074037208.1 DNA translocase FtsK [Exiguobacterium profundum] | 0.5377 | 48 |
| WP_074035172.1 ABC transporter permease [Exiguobacterium profundum] | 0.5367 | 41 |
| WP_074034892.1 alanine:cation symporter family protein [Exiguobacterium profundum] | 0.5363 | 36 |
| WP_074036869.1 ATP-binding cassette domain-containing protein [Exiguobacterium profundum] | 0.5361 | 30 |
| WP_159562038.1 MULTISPECIES: DUF5325 family protein [Exiguobacterium] | 0.5358 | 18 |
| WP_074037113.1 AmiS/UreI transporter [Exiguobacterium profundum] | 0.5357 | 53 |
| WP_074036440.1 bacteriorhodopsin [Exiguobacterium profundum] | 0.5355 | 56 |
| WP_074037549.1 Na(+)/H(+) antiporter subunit C [Exiguobacterium profundum] | 0.5351 | 53 |
| WP_074037915.1 PadR family transcriptional regulator [Exiguobacterium profundum] | 0.5351 | 18 |
| WP_074036444.1 hypothetical protein [Exiguobacterium profundum] | 0.5349 | 23 |
| WP_074035503.1 solute:sodium symporter family transporter [Exiguobacterium profundum] | 0.5347 | 61 |
| WP_074037457.1 ABC transporter ATP-binding protein [Exiguobacterium profundum] | 0.5345 | 36 |
| WP_074035234.1 MULTISPECIES: cell wall metabolism sensor histidine kinase WalK [Exiguobacterium] | 0.5343 | 30 |
| WP_074036238.1 DUF3021 family protein [Exiguobacterium profundum] | 0.534 | 28 |
| WP_074036040.1 MBOAT family protein [Exiguobacterium profundum] | 0.5338 | 27 |
| WP_074034928.1 hypothetical protein [Exiguobacterium profundum] | 0.5332 | 42 |
| WP_074037623.1 rhodanese-like domain-containing protein [Exiguobacterium profundum] | 0.533 | 24 |
| WP_074036849.1 MFS transporter [Exiguobacterium profundum] | 0.5328 | 35 |
| WP_084543022.1 D-alanyl-D-alanine carboxypeptidase [Exiguobacterium profundum] | 0.5321 | 26 |
| WP_074037993.1 MBL fold metallo-hydrolase [Exiguobacterium profundum] | 0.5319 | 25 |
| WP_047795512.1 MULTISPECIES: hypothetical protein [Exiguobacterium] | 0.5313 | 24 |
| WP_074034783.1 hypothetical protein [Exiguobacterium profundum] | 0.5311 | 33 |
| WP_074036317.1 hypothetical protein [Exiguobacterium profundum] | 0.5308 | 27 |
| WP_143180254.1 MULTISPECIES: DUF2929 family protein [Exiguobacterium] | 0.5302 | 30 |
| WP_047795807.1 MULTISPECIES: LlsX family protein [Exiguobacterium] | 0.5301 | 38 |
| WP_074035258.1 membrane protein insertase YidC [Exiguobacterium profundum] | 0.5297 | 31 |
| WP_074038007.1 hypothetical protein [Exiguobacterium profundum] | 0.5291 | 20 |
| WP_012727651.1 MULTISPECIES: DNA-directed RNA polymerase subunit alpha [Exiguobacterium] | 0.527 | 55 |
| WP_074035717.1 hypothetical protein [Exiguobacterium profundum] | 0.5266 | 21 |
| WP_074036266.1 hypothetical protein [Exiguobacterium profundum] | 0.5264 | 22 |
| WP_074037676.1 foldase [Exiguobacterium profundum] | 0.5251 | 38 |
| WP_074037157.1 MULTISPECIES: hypothetical protein [Exiguobacterium] | 0.5249 | 19 |
| WP_074036624.1 substrate-binding domain-containing protein [Exiguobacterium profundum] | 0.5247 | 24 |
| WP_026826530.1 MULTISPECIES: Na(+)/H(+) antiporter subunit F1 [Exiguobacterium] | 0.5242 | 28 |
| WP_074035722.1 MULTISPECIES: F0F1 ATP synthase assembly protein I [Exiguobacterium] | 0.5242 | 53 |
| WP_143180212.1 hypothetical protein [Exiguobacterium profundum] | 0.5237 | 56 |
| WP_012727692.1 MULTISPECIES: preprotein translocase subunit SecE [Exiguobacterium] | 0.5232 | 45 |
| WP_074036946.1 DM13 domain-containing protein [Exiguobacterium profundum] | 0.5229 | 22 |
| WP_074034707.1 CPBP family intramembrane metalloprotease [Exiguobacterium profundum] | 0.5225 | 41 |
| WP_074035239.1 hypothetical protein [Exiguobacterium profundum] | 0.5204 | 39 |
| WP_074035786.1 HAMP domain-containing histidine kinase [Exiguobacterium profundum] | 0.5203 | 25 |
| WP_074036974.1 MULTISPECIES: hypothetical protein [Exiguobacterium] | 0.5197 | 42 |
| WP_074034773.1 MFS transporter [Exiguobacterium profundum] | 0.5179 | 22 |
| WP_074035621.1 cardiolipin synthase [Exiguobacterium profundum] | 0.5177 | 30 |
| WP_074037178.1 DUF1405 domain-containing protein [Exiguobacterium profundum] | 0.5177 | 30 |
| WP_143180218.1 hypothetical protein [Exiguobacterium profundum] | 0.5177 | 29 |
| WP_074037742.1 hypothetical protein [Exiguobacterium profundum] | 0.5155 | 22 |
| WP_160300237.1 MULTISPECIES: hypothetical protein [Exiguobacterium] | 0.515 | 45 |
| WP_143180250.1 MULTISPECIES: protein translocase subunit SecD [Exiguobacterium] | 0.5141 | 24 |
| WP_074037332.1 MULTISPECIES: phospho-N-acetylmuramoyl-pentapeptide-transferase [Exiguobacterium] | 0.514 | 20 |
| WP_074037343.1 PDZ domain-containing protein [Exiguobacterium profundum] | 0.5129 | 23 |
| WP_074035423.1 M23 family metallopeptidase [Exiguobacterium profundum] | 0.5123 | 23 |
| WP_012727460.1 MULTISPECIES: DUF350 domain-containing protein [Exiguobacterium] | 0.512 | 33 |
| WP_015880934.1 MULTISPECIES: hypothetical protein [Exiguobacterium] | 0.5117 | 24 |
| WP_074035368.1 MULTISPECIES: hypothetical protein [Exiguobacterium] | 0.5117 | 21 |
| WP_074037244.1 hypothetical protein [Exiguobacterium profundum] | 0.5113 | 27 |
| WP_074035414.1 hypothetical protein [Exiguobacterium profundum] | 0.5108 | 21 |
| WP_012726551.1 MULTISPECIES: BrxA/BrxB family bacilliredoxin [Exiguobacterium] | 0.5101 | 57 |
| WP_074036791.1 hypothetical protein [Exiguobacterium profundum] | 0.51 | 23 |
| WP_074037429.1 DUF3899 domain-containing protein [Exiguobacterium profundum] | 0.5097 | 22 |
| WP_074035974.1 short chain dehydrogenase [Exiguobacterium profundum] | 0.5082 | 17 |
| WP_074036178.1 hypothetical protein [Exiguobacterium profundum] | 0.507 | 39 |
| WP_015881463.1 MULTISPECIES: DUF2768 domain-containing protein [Exiguobacterium] | 0.5059 | 30 |
| WP_143180268.1 methyl-accepting chemotaxis protein [Exiguobacterium profundum] | 0.5058 | 46 |
| WP_074035450.1 two pore domain potassium channel family protein [Exiguobacterium profundum] | 0.5054 | 58 |
| WP_012726942.1 MULTISPECIES: YitT family protein [Exiguobacterium] | 0.5052 | 34 |
| WP_074035099.1 DMT family transporter [Exiguobacterium profundum] | 0.5043 | 19 |
| WP_074035893.1 hypothetical protein [Exiguobacterium profundum] | 0.5042 | 39 |
| WP_074037046.1 MFS transporter [Exiguobacterium profundum] | 0.5038 | 57 |
| WP_074037520.1 hypothetical protein [Exiguobacterium profundum] | 0.5038 | 19 |
| WP_074036553.1 MULTISPECIES: PTS sugar transporter subunit IIC [Exiguobacterium] | 0.5036 | 37 |
| WP_074035713.1 hypothetical protein [Exiguobacterium profundum] | 0.5034 | 23 |
| WP_074036064.1 cell wall-binding protein [Exiguobacterium profundum] | 0.5032 | 25 |
| WP_074035691.1 UDP-N-acetylglucosamine 1-carboxyvinyltransferase [Exiguobacterium profundum] | 0.5021 | 40 |
| WP_074035458.1 diguanylate cyclase [Exiguobacterium profundum] | 0.5014 | 19 |
| WP_074037847.1 CPBP family intramembrane metalloprotease [Exiguobacterium profundum] | 0.5013 | 27 |
| WP_074037793.1 fluoride efflux transporter CrcB [Exiguobacterium profundum] | 0.5012 | 16 |
| WP_074037721.1 MULTISPECIES: YihY/virulence factor BrkB family protein [Exiguobacterium] | 0.5011 | 62 |
| WP_074036062.1 DUF1343 domain-containing protein [Exiguobacterium profundum] | 0.5009 | 25 |
| WP_074034721.1 hypothetical protein [Exiguobacterium profundum] | 0.5004 | 39 |
| WP_074036272.1 excinuclease ABC subunit UvrA [Exiguobacterium profundum] | 0.5 | 45 |

**Supplementary Table 4:** Gene ontology annotation of upregulated genes

|  | **Gene Name** | **Gene Id** | **Protein Name** | **GO** | **Biological Process** | **Molecular Function** | **Cellular Component** |
| --- | --- | --- | --- | --- | --- | --- | --- |
| 1 | EAT1b_0795 | C4L4Y1 | Amino acid carrier protein | GO:0015655 |  | alanine:sodium symporter activity [GO:0015655] |  |
| 2 | azoR EAT1b_2007 | C4L0W8 | FMN-dependent NADH-azoreductase; Azo-dye reductase; FMN-dependent NADH-azo compound oxidoreductase | GO:0009055;GO:0010181;GO:0016652;  GO:0016655 |  | electron transfer activity[GO:0009055];  FMN binding [GO:0010181]; oxidoreductase activity, acting on NAD(P)H, NAD(P) as acceptor [GO:0016652];  oxidoreductase activity, acting on NAD(P)H, quinone or similar compound as acceptor [GO:0016655] |  |
| 3 | CO1 COX1 | A0A0F7BHZ3 | Cytochrome c oxidase subunit 1 | GO:0016021;GO:0005743;GO:0045277;  GO:0004129;GO:0020037;GO:0005506;  GO:0009060;GO:0006119 | aerobic respiration [GO:0009060];oxidative phosphorylation [GO:0006119] | cytochrome-c oxidase activity [GO:0004129];heme binding [GO:0020037];iron ion binding [GO:0005506] | integral component of membrane [GO:0016021];mitochondrial inner membrane [GO:0005743];respiratory chain complex IV [GO:0045277] |
| 4 | EAT1b_1455 | C4KZ69 | Peptidase M23 | - | - | - | - |
| 5 | MT-CO2 COII |  | - | - | - | - | - |
| 6 | EAT1b_2177 | C4L1R8 | Drug resistance transporter | GO:0022857; |  | transmembrane transporter activity [GO:0022857] |  |
| 7 | topA EAT1b_2905 | C4L613 | DNA topoisomerase 1; DNA topoisomerase I | GO:0003677;GO:0003917;  GO:0046872;GO:0006265 | DNA topological change [GO:0006265] | DNA binding [GO:0003677];DNA topoisomerase type I (single strand cut, ATP-independent) activity [GO:0003917];metal ion binding [GO:0046872] |  |
| 8 | EAT1b_1809 | C4L072 | Alkyl hydroperoxide reductase | GO:0008785;GO:0050660;GO:0051287;  GO:0000302 | response to reactive oxygen species[GO:0000302] | alkyl hydroperoxide reductase activity [GO:0008785];flavin adenine dinucleotide binding [GO:0050660];NAD binding [GO:0051287] |  |
| 9 | EAT1b_1679 | C4KZU2 | YhgE/Pip N-terminal domain protein | GO:0016021;GO:0015297 |  | antiporter activity [GO:0015297] | integral component of membrane [GO:0016021] |
| 10 | EAT1b_1994 | C4L0V5 | Na+/H+ antiporter NhaC | GO:0016021;GO:0015297 |  | antiporter activity [GO:0015297] | integral component of membrane [GO:0016021] |
| 11 | alb | I6U3L8 | Albumin | GO:0005615 |  |  | extracellular space [GO:0005615] |
| 12 | EAT1b_1982 | C4L0U3 | Major facilitator superfamily MFS_1 | GO:0016021;GO:0055085 | transmembrane transport [GO:0055085] |  | integral component of membrane [GO:0016021] |
| 13 | glpK EAT1b_0887 | C4L5K1 | Glycerol kinase; ATP:glycerol 3-phosphotransferase; Glycerokinase; GK | GO:0005524;GO:0004370;GO:0019563;  GO:0006072 | glycerol catabolic process [GO:0019563];glycerol-3-phosphate metabolic process [GO:0006072] | ATP binding [GO:0005524];glycerol kinase activity [GO:0004370] |  |
| 14 | apoVLDL-II Anapl_05886 | Q5QFI1 | Apovitellenin-1; Apovitellenin I | GO:0042627;GO:0034361;GO:0004857;  GO:0045735;GO:0006629 | lipid metabolic process [GO:0006629] | enzyme inhibitor activity [GO:0004857];nutrient reservoir activity [GO:0045735] | chylomicron [GO:0042627];very-low-density lipoprotein particle [GO:0034361] |
| 15 | EAT1b_0780 | C4L4W6 | Peptidase S8 and S53 subtilisin kexin sedolisin | GO:0004866;GO:0004252 |  | endopeptidase inhibitor activity [GO:0004866];serine-type endopeptidase activity [GO:0004252] |  |
| 16 | rplA EAT1b_1645 | C4KZQ8 | 50S ribosomal protein L1 | GO:0015934;GO:0019843;GO:0003735;  GO:0000049;GO:0006417;GO:0006412 | regulation of translation [GO:0006417];translation [GO:0006412] | rRNA binding [GO:0019843];structural constituent of ribosome [GO:0003735];tRNA binding [GO:0000049] | large ribosomal subunit [GO:0015934] |
| 17 | EAT1b_0568 | C4L3K2 | Na/Pi-cotransporter II-related protein | GO:0016021;GO:0015321 |  | sodium-dependent phosphate transmembrane transporter activity [GO:0015321] | integral component of membrane [GO:0016021] |
| 18 | EAT1b_1263 | C4KYM7 | Multicopper oxidase type 3 | GO:0016021;GO:0005507;GO:0016491 |  | copper ion binding [GO:0005507];oxidoreductase activity [GO:0016491] | integral component of membrane [GO:0016021] |
| 19 | EAT1b_2477 | C4L3Q3 | Acriflavin resistance protein | GO:0022857 |  | transmembrane transporter activity [GO:0022857] |  |
| 20 | EAT1b_2566 | C4L4C1 | Peptidase M23 | - | - | - | - |
| 21 | rpoC EAT1b_1640 | C4KZQ3 | DNA-directed RNA polymerase subunit beta'; RNAP subunit beta'; RNA polymerase subunit beta'; Transcriptase subunit beta' | GO:0003677;GO:0003899;GO:0000287;  GO:0008270;GO:0006351 | transcription, DNA-templated [GO:0006351] | DNA binding[GO:0003677]; DNA-directed 5'-3' RNA polymerase activity[GO:0003899]; magnesium ion binding[GO:0000287]; zinc ion binding [GO:0008270] |  |
| 22 | lutB EAT1b_1960 | C4L0S1 | Lactate utilization protein B | GO:0051539;GO:0046872;GO:0019516 | lactate oxidation [GO:0019516] | 4 iron, 4 sulfur cluster binding[GO:0051539];metal ion binding [GO:0046872] |  |
| 23 | FTH1 | L7XWS8 | Ferritin | GO:0005623;GO:0008199;GO:0006879;  GO:0006826 | cellular iron ion homeostasis [GO:0006879];iron ion transport [GO:0006826] | ferric iron binding [GO:0008199] | cell [GO:0005623] |
| 24 | purH EAT1b_2233 | C4L2A3 | Bifunctional purine biosynthesis protein PurH; IMP cyclohydrolase; ATIC; Inosinicase; IMP synthase; Phosphoribosylaminoimidazolecarboxamide formyltransferase; AICAR transformylase | GO:0003937;GO:0004643;GO:0006189 | de novo' IMP biosynthetic process [GO:0006189] | IMP cyclohydrolase activity [GO:0003937];phosphoribosylaminoimidazolecarboxamide formyltransferase activity [GO:0004643] |  |
| 25 | bshC EAT1b_2830 | C4L5T8 | Putative cysteine ligase BshC | GO:0016874 |  | Ligase activity [GO:0016874] |  |
| 26 | EAT1b_1066 | C4L6G3 | Uncharacterized protein | - | - | - | - |
| 27 | EAT1b_2108 | C4L1J9 | Transport system permease protein | GO:0016021;GO:0005886;GO:0005215 |  | transporter activity [GO:0005215] | integral component of membrane [GO:0016021];plasma membrane [GO:0005886] |
| 28 | EAT1b_2487 | C4L3R3 | Histidine kinase | GO:0016021;GO:0005622;GO:0000155 |  | phosphorelay sensor kinase activity [GO:0000155] | integral component of membrane [GO:0016021];intracellular [GO:0005622] |
| 29 | EAT1b_2050 | C4L110 | Amino acid carrier protein | GO:0016021;GO:0005886;GO:0015655 |  | alanine [GO:0015655] | integral component of membrane [GO:0016021];plasma membrane [GO:0005886] |
| 30 | EAT1b_0307 | C4L266 | LPXTG-motif cell wall anchor domain protein | GO:0016021 |  |  | integral component of membrane [GO:0016021] |
| 31 | prfC EAT1b_0394 | C4L2R8 | Peptide chain release factor 3; RF-3 | GO:0003924;GO:0005525;GO:0016149;GO:0006449 | regulation of translational termination [GO:0006449] | GTPase activity [GO:0003924];GTP binding [GO:0005525];translation release factor activity, codon specific [GO:0016149] |  |
| 32 | EAT1b_0565 | C4L3J9 | Penicillin-binding protein transpeptidase | GO:0016021;GO:0008658 |  | penicillin binding [GO:0008658] | integral component of membrane [GO:0016021] |
| 33 | EAT1b_1319 | C4KYT3 | Uncharacterized protein | - | - | - | - |

**Supplementary Table 5: FPKM (Fragments Per Kilobase of transcript per Million mapped reads) and reads count of downregulated and upregulated genes**

| **Downregulated Gene** | **0mM salt**  **Read Count** | **0mM salt FPKM** | **100mM salt Read Count** | **100mM salt FPKM** |
| --- | --- | --- | --- | --- |
| Uncharacterized protein (A6395_05075) | 84424 | 1791.24 | 4181 | 124.5903 |
| Extracellular solute-binding protein family 5 (EAT1b_2734) | 16476 | 642.0715 | 764 | 41.81587 |
| Uncharacterized protein | 126 | 22.45766 | 3 | 0.750985 |
| Uncharacterized protein (RBCS-A Os12g0291100) | 35 | 3.55552 | 0 | 0 |
| Uncharacterized protein (JS80_02470) | 2772 | 394.236 | 174 | 34.7559 |
| Uncharacterized protein (psbD) | 96 | 3.56778 | 2 | 0.104393 |
| Uncharacterized protein (psbA) | 1727 | 99.02197 | 17 | 1.369003 |
| Uncharacterized protein | 75 | 17.53655 | 0 | 0 |
| Uncharacterized protein (EAT1b_0843) | 4131 | 337.6118 | 130 | 14.92182 |
| Uncharacterized protein (BN1080_00055) | 146699 | 42876.57 | 8606 | 3532.729 |
| Uncharacterized protein (BN1080_00055) | 38544 | 10634.61 | 1911 | 740.5283 |
| Excalibur domain protein (EAT1b_0257) | 1133 | 224.572 | 46 | 12.80559 |
| uncharacterized protein LOC108265481 | 30 | 2.639429 | 0 | 0 |
| Uncharacterized protein (rbcL) | 81 | 5.392996 | 0 | 0 |
| Uncharacterized protein (OsI_34215) | 54 | 12.54129 | 0 | 0 |
| Uncharacterized protein | 42 | 9.689089 | 0 | 0 |
| Integral membrane protein TerC (EAT1b_1254) | 3830 | 93.78141 | 213 | 7.325103 |
| Flagellin (AWH56_17980) | 85799 | 6126.465 | 1630 | 163.4675 |
| apolipoprotein A-I-2-like (LOC108280792) | 40 | 2.650416 | 0 | 0 |
| Uncharacterized protein | 41 | 9.39555 | 0 | 0 |
| Uncharacterized protein | 54 | 12.7125 | 0 | 0 |

| **Upregulated Gene** | **0mM salt Read Count** | **0mM salt FPKM** | **100 mM salt Read Count** | **100mM salt FPKM** |
| --- | --- | --- | --- | --- |
| Amino acid carrier protein | 293 | 6.933203 | 2084 | 69.25963 |
| FMN-dependent NADH-azoreductase; Azo-dye reductase; FMN-dependent NADH-azo compound | 30 | 3.025311 | 260 | 36.82462 |
| Cytochrome c oxidase subunit 1 | 0 | 0 | 24 | 4.107862 |
| Peptidase M23 | 129 | 5.315439 | 4304 | 249.0794 |
| Uncharacterized protein CO1 COX1 | 0 | 0 | 17 | 3.167135 |
| Drug resistance transporter | 992 | 44.00339 | 9998 | 622.8791 |
| DNA topoisomerase 1; DNA topoisomerase I | 1311 | 20.56606 | 9365 | 206.3347 |
| Alkyl hydroperoxide reductase | 849 | 22.61064 | 16024 | 599.3661 |
| YhgE/Pip N-terminal domain protein | 207 | 3.923676 | 1420 | 37.80309 |
| Na+/H+ antiporter NhaC | 100 | 4.67008 | 15841 | 1039.018 |
| Albumin | 0 | 0 | 84 | 7.779803 |
| Major facilitator superfamily MFS_1 | 45 | 2.507245 | 1467 | 114.797 |
| Glycerol kinase; ATP:glycerol 3-phosphotransferase; Glycerokinase; GK | 8388 | 69.53248 | 68662 | 799.3959 |
| Apovitellenin-1; Apovitellenin I | 0 | 0 | 80 | 10.77909 |
| Peptidase S8 and S53 subtilisin kexin sedolisin | 3218 | 37.19307 | 21634 | 351.1794 |
| 50S ribosomal protein L1 | 5078 | 74.77917 | 39037 | 807.3848 |
| Na/Pi-cotransporter II-related protein | 377 | 9.959541 | 2327 | 86.33969 |
| Multicopper oxidase type 3 | 303 | 7.196989 | 2858 | 95.34252 |
| Acriflavin resistance protein | 222 | 4.072583 | 1751 | 45.11483 |
| Peptidase M23 | 160 | 4.817256 | 3023 | 127.8304 |
| DNA-directed RNA polymerase subunit beta'; RNAP subunit beta'; RNA polymerase subunit beta'; Transcriptase subunit beta' | 52742 | 52.08736 | 354426 | 491.6063 |
| Lactate utilization protein B | 1427 | 34.09432 | 9771 | 327.8787 |
| Ferritin | 0 | 0 | 17 | 2.141626 |
| Bifunctional purine biosynthesis protein PurH; IMP cyclohydrolase; ATIC; Inosinicase; IMP synthase; Phosphoribosylaminoimidazolecarboxamide formyltransferase; AICAR transformylase | 225 | 3.489174 | 1480 | 32.23428 |
| Putative cysteine ligase BshC | 144 | 3.385378 | 900 | 29.71689 |
| Uncharacterized protein | 4284 | 17.05714 | 39300 | 219.7685 |
| Transport system permease protein | 185 | 3.23795 | 4037 | 99.23693 |
| Histidine kinase | 1430 | 28.01398 | 11585 | 318.7506 |
| Amino acid carrier protein | 74 | 3.297354 | 727 | 45.49718 |
| LPXTG-motif cell wall anchor domain protein | 1623 | 69.96863 | 10561 | 639.4492 |
| Peptide chain release factor 3; RF-3 | 266 | 7.774536 | 1868 | 76.68066 |
| Penicillin-binding protein transpeptidase | 303 | 9.604806 | 5048 | 224.7406 |
| Uncharacterized protein | 325 | 11.27077 | 2144 | 104.4266 |
